# Antimicrobial Properties of Flavonoid Derivatives with Bromine, Chlorine, and Nitro Group Obtained by Chemical Synthesis and Biotransformation Studies

**DOI:** 10.3390/ijms25105540

**Published:** 2024-05-19

**Authors:** Martyna Perz, Daria Szymanowska, Tomasz Janeczko, Edyta Kostrzewa-Susłow

**Affiliations:** 1 Department of Food Chemistry and Biocatalysis, Faculty of Biotechnology and Food Science, Wrocław University of Environmental and Life Sciences, 50-375 Wrocław, Poland; tomasz.janeczko@upwr.edu.pl; 2 Department of Biotechnology and Food Microbiology, Faculty of Food Science and Nutrition, Poznań University of Life Sciences, 60-627 Poznań, Poland; daria.szymanowska@up.poznan.pl; 3 Department of Pharmacognosy and Biomaterials, Faculty of Pharmacy, Poznań University of Medical Sciences, 60-806 Poznań, Poland

**Keywords:** biotransformations, entomopathogenic filamentous fungi, *Beauveria bassiana*, bromine, chlorine, nitro group, antimicrobial activity, pathogenic bacteria, probiotic bacteria

## Abstract

The search for new substances of natural origin, such as flavonoids, is necessary in the fight against the growing number of diseases and bacterial resistance to antibiotics. In our research, we wanted to check the influence of flavonoids with chlorine or bromine atoms and a nitro group on pathogenic and probiotic bacteria. We synthesized flavonoids using Claisen–Schmidt condensation and its modifications, and through biotransformation via entomopathogenic filamentous fungi, we obtained their glycoside derivatives. Biotransformation yielded two new flavonoid glycosides: 8-amino-6-chloroflavone 4′-*O*-*β*-D-(4″-*O*-methyl)-glucopyranoside and 6-bromo-8-nitroflavone 4′-*O*-*β*-D-(4″-*O*-methyl)-glucopyranoside. Subsequently, we checked the antimicrobial properties of the aforementioned aglycon flavonoid compounds against pathogenic and probiotic bacteria and yeast. Our studies revealed that flavones have superior inhibitory effects compared to chalcones and flavanones. Notably, 6-chloro-8-nitroflavone showed potent inhibitory activity against pathogenic bacteria. Conversely, flavanones 6-chloro-8-nitroflavanone and 6-bromo-8-nitroflavanone stimulated the growth of probiotic bacteria (*Lactobacillus acidophilus* and *Pediococcus pentosaceus*). Our research has shown that the presence of chlorine, bromine, and nitro groups has a significant effect on their antimicrobial properties.

## 1. Introduction

Flavonoids are a class of low-molecular-weight polyphenolic compounds. In terms of chemical composition, the basic structure of flavonoids is a C6-C3-C6 skeleton, usually characterized by a cyclic system with oxygen in a three-carbon bridge [1,2]. Flavonoids are known for their anti-inflammatory, antioxidant, and antimicrobial properties [1]. However, due to their low water solubility, orally administered flavonoids are poorly absorbed, and therefore, their therapeutic potential is limited [3]. Glycosylation has proven to be an effective way to increase the water solubility and bioavailability of flavonoids [4]. It is worth adding that the chemical attachment of a glucose molecule to a flavonoid compound is very challenging and often even impossible [5]. Utilizing biotransformation through entomopathogenic strains of filamentous fungi is an alternative to multi-stage traditional methods [4,6]. This method does not require toxic reaction reagents and highly specialized equipment [7,8]. Many studies report that the use of entomopathogenic strains of filamentous fungi is an effective method of obtaining flavonoid glycosides [8,9,10,11].

Numerous studies suggest that flavonoids exhibit both stimulatory and inhibitory effects on different species of microbial growth [12,13,14,15]. These significant biological activities characteristic of flavonoids with bromine, chlorine atoms, and nitro groups hold implications for their practical use. Studies report that flavonoids containing bromine or chlorine atoms in their structure have a significant effect on bacteria. Hurtová et al. proved that all flavonolignans derivatives inhibited bacterial quorum sensing (AI-2 type) and adhesion to the surface of *Staphyloccocus aureus* and *Pseudomonas aeruginosa*, preventing biofilm formation [16]. Also, in the case of quercetin, or its derivative containing a chlorine atom or atoms, had more potent antioxidant properties [17]. Similarly, sulfur and nitrogen atoms, in addition to substituents at certain positions, enhanced the antibacterial activity of flavone derivatives [18]. Mughal et al. discovered that the substituents of synthesized flavones, 4-thioflavones, and 4-iminoflavones are responsible for the enhancement of the antibacterial activity. They observed that the percentage of inhibition rises with the increasing electronegativity of the halogen atom in the A-ring. Moreover, sulfur and nitrogen atoms, along with certain positions of substituents, contribute to enhancing the antibacterial properties of flavone derivatives. The activities demonstrated by thioflavones and iminoflavones may stem from the combined impact of heteroatoms and substituents within the system [19].

Furthermore, halogenated flavonoids can not only inhibit bacterial growth but also increase the binding affinity to human protein kinase CK2 (hCK2α) [20], anticancer properties [21], or anti-inflammatory effects [22,23]. These days, diseases caused by bacteria and the growing resistance of bacteria to antibiotics pose a significant threat to human health. It is necessary to search for new substances of natural origin in the fight against chronic diseases.

The purpose of this study was a biotransformation of flavonoids with nitro group and bromine or chlorine atoms to obtain glycosidic derivatives. Due to that fact, we created a library on new halogenated derivatives of flavonoids aglycones and glycosides. The second aim was to investigate the effects of produced flavonoids on probiotic and pathogenic bacteria and yeast.

## 2. Results

### 2.1. Chemical Synthesis

**Figure 1:**
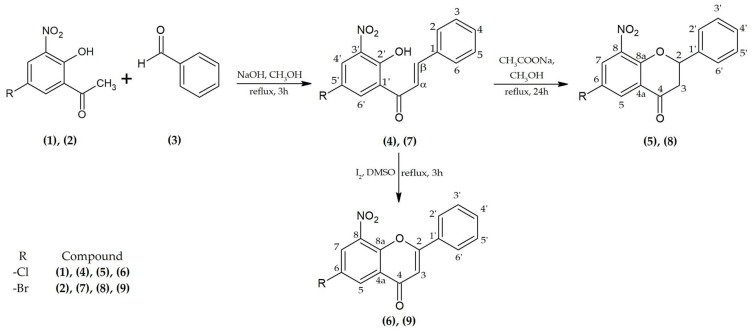
Scheme of the synthesis of flavonoid compounds (**4**–**9**).

As a result of chemical synthesis of flavonoids (**4**–**9**) carried out via the synthesis shown in Figure 1, all the compounds were obtained. Obtained products were analyzed structurally with the use of nuclear magnetic resonance (NMR), and masses were confirmed by liquid chromatography–mass spectrometry (LC-MS).

According to the synthesis of 6-chloro-8-nitroflavanone (**5**) and 6-bromo-8-nitroflavanone (**8**), the process had low efficiency. As a result, a mixture of chalcone and flavanone was obtained. It was impossible to separate due to the very similar retention time of the compounds (shown below in data). Therefore, we decided to carry out only biological tests on these compounds and resign from biotransformation.

5′-Chloro-2′-hydroxy-3′-nitrochalcone (**4**). C_15_H_10_ClNO_4_, mp: 151–167 °C; HPLC Rt: 17.9 min; **^1^H NMR** (600 MHz, acetone-d6) δ (ppm): 8.45 (1H, s, H-6′), 8.20 (1H, d, *J* = 2.6 Hz, H-4′), 8.09 (1H, d, *J* = 15.5 Hz, H-α), 7.94 (1H, d, *J* = 15.5 Hz, H-β), 7.89–7.86 (2H, m, H-2, H-6), 7.52–7.45 (3H, m, H-3, H-4, H-5); **^13^C NMR** (acetone-d6) δ (ppm): 193.23 (C=O), 158.15 (C-2′), 147.08 (C-β), 140.10 (C-3′), 136.23 (C-6′), 135.64 (C-1), 132.08 (C-4), 131.21 (C-4′), 130.13 (C-2, C-6), 129.91 (C-3, C-5), 127.57 (C-1′), 122.88 (C-α), 121.36 (C-5′).

6-Chloro-8-nitroflavanone (**5**). C_15_H_10_ClNO_4_, mp: 135–153 °C; HPLC Rt: 17.163 min; [α]_D_ = −0.188 (0.5 *w*/*v* % in acetone); **^1^H NMR** (600 MHz, acetone-d6) δ (ppm): 8.27–8.25 (1H, m, H-7), 8.09–8.07 (1H, m, H-5), 7.64–7.59 (2H, m, H-2′, H-6′), 7.51–7.39 (3H, m, H-3′, H-4′, H-5′), 5.96 (1H, dd, *J* = 12.9, 3.0 Hz, H-2), 3.39 (1H, dd, *J* = 17.0, 12.9 Hz, H-3eq), 3.13 (1H, dd, *J* = 17.0, 3.0 Hz, H-3ax); **^13^C NMR** (acetone-d6) δ (ppm): 189.27 (C-4), 153.74 (C-8a), 141.46 (C-8), 138.90 (C-1′), 131.45 (C-5), 131.26 (C-7), 129.74 (C-4′), 129.63 (C-3′, C-5′), 127.16 (C-2′, C-6′), 126.25 (C-6), 125.38 (C-4a), 81.85 (C-2), 44.04 (C-3).

6-Chloro-8-nitroflavone (**6**). C_15_H_8_ClNO_4_, mp: 178–186 °C; HPLC Rt: 17.273 min; **^1^H NMR** (600 MHz, acetone-d6) δ (ppm): 8.55 (1H, d, *J* = 2.7 Hz, H-7), 8.36 (1H, d, *J* = 2.7 Hz, H-5), 8.17–8.13 (2H, m, H-2′, H-6′), 7.69–7.62 (3H, m, H-3′, H-4′, H-5′), 7.09 (1H, s, H-3); **^13^C NMR** (acetone-d6) δ (ppm): 175.17 (C-4), 164.40 (C-2), 148.18 (C-8a), 140.63 (C-8), 133.30 (C-4′), 131.57 (C-1′), 130.97 (C-5), 130.76 (C-7), 130.48 (C-6), 130.19 (C-3′, C-5′), 127.74 (C-4a), 127.57 (C-2′, C-6′), 108.06 (C-3).

5′-Bromo-2′-hydroxy-3′-nitrochalcone (**7**). C_15_H_10_BrNO_4_, mp: 173–175 °C; HPLC Rt: 18.073 min; **^1^H NMR** (600 MHz, acetone-d6) δ (ppm): 8.08 (1H, d, *J* = 3.1 Hz, H-4′), 8.06 (1H, d, *J* = 15.8 Hz, H-α), 7.83 (1H, d, *J* = 3.1 Hz, H-6′), 7.69–7.66 (2H, m, H-2, H-6), 7.59 (1H, d, *J* = 15.8 Hz, H-β), 7.41–7.36 (3H, m, H-3, H-4, H-5); **^13^C NMR** (acetone-d6) δ (ppm): 191.08 (C=O), 167.80 (C-2′), 141.27 (C-3′), 140.94 (C-β), 140.13 (C-6′), 137.42 (C-1′), 136.84 (C-1), 133.82 (C-4′), 130.49 (C-4), 129.66 (C-3, C-5), 129.21 (C-2, C-6), 128.30 (C-α), 98.00 (C-5′).

6-Bromo-8-nitroflavanone (**8**). C_15_H_10_BrNO_4_, mp: 133–140 °C; HPLC Rt: 17.310 min; [α]_D_ = −4.576 (0.715 *w*/*v* % in acetone); **^1^H NMR** (600 MHz, acetone-d6) δ (ppm): 8.36 (1H, d, *J* = 2.6 Hz, H-7), 8.20 (1H, d, *J* = 2.6 Hz, H-5), 7.63–7.60 (2H, m, H-2′, H-6′), 7.50–7.46 (2H, m, H-3′, H-5′), 7.44–7.40 (1H, m, H-4′), 5.96 (1H, dd, *J* = 12.9, 3.0 Hz, H-2), 3.38 (1H, dd, *J* = 17.0, 12.9 Hz, H-3eq), 3.13 (1H, dd, *J* = 17.0, 3.0 Hz, H-3ax); **^13^C NMR** (acetone-d6) δ (ppm): 189.18 (C-4), 154.15 (C-8a), 141.66 (C-8), 138.88 (C-1′), 134.47 (C-5), 133.96 (C-7), 129.75 (C-4′), 129.64 (C-3′, C-5′), 127.16 (C-2′, C-6′), 125.71 (C-4a), 112.75 (C-6), 81.85 (C-2), 44.01 (C-3).

6-Bromo-8-nitroflavone (**9**). C_15_H_8_BrNO_4_, mp: 198 °C; HPLC Rt: 17.480 min; **^1^H NMR** (600 MHz, acetone-d6) δ (ppm): 8.66 (1H, d, *J* = 2.5 Hz, H-7), 8.50 (1H, d, *J* = 2.5 Hz, H-5), 8.16–8.13 (2H, m, H-2′, H-6′), 7.69–7.62 (3H, m, H-3′, H-4′, H-5′), 7.09 (1H, s, H-3); **^13^C NMR** (acetone-d6) δ (ppm): 175.05 (C-4), 164.38 (C-2), 148.57 (C-8a), 140.65 (C-8), 134.12 (C-5), 133.40 (C-7), 133.30 (C-4′), 131.56 (C-1′), 130.18 (C-3′, C-5′), 127.97 (C-4a), 127.57 (C-2′, C-6′), 117.32 (C-6), 108.14 (C-3).

The coupling constants, multiplicity, and chemical shifts of proton and carbon atom signals clearly confirm that the planned compounds (**4**–**9**) have been obtained. The LC-MS and NMR spectra and their detailed analysis with the assignment of carbon atoms and protons to the signals of individual compounds were presented in the Supplementary Materials for compound (**4**) p. 1–7 Figures S1–S13; (**5**) p. 8–14, Figures S14–S27; (**6**) p. 15–21, Figures S28–S41; (**7**) p. 29–35, Figures S33–S45; (**8**) p. 35–43, Figures S46–S61; and (**9**) p. 43–50, Figures S62–S75.

### 2.2. Biotransformation

Following the biotransformation of substrates (**4**), (**7**), (**6**), and (**9**) in the cultures of *B. bassiana* KCH J1.5, *I. fumosorosea* KCH J2, and *I. farinosa* KCH J2.6, glycoside derivatives of flavonoids were successfully obtained from compounds (**6**) and (**9**). The resulting products were isolated from the fungal cultures and subjected to purification through preparative thin-layer chromatography (pTLC). Structural analyses of the obtained products were conducted using nuclear magnetic resonance (NMR), while mass confirmations were accomplished through liquid chromatography–mass spectrometry (LC-MS) (Supplementary Materials).

Due to the low efficiency according to the synthesis of compounds (**5**) and (**8**), they were not subjected to biotransformation. Chalcones (**4**) and (**7**) were not successfully biotransformed, and a large amount of substrates remained unreacted in the cultures of selected entomopathogenic filamentous fungi, resulting in the absence of flavonoid glycoside formation.

8-amino-6-chloroflavone 4′-*O-β*-D-(4″-*O*-methyl)-glucopyranoside (**6a**). C_22_H_22_ClNO_8_, mp: 59–63 °C; HPLC Rt = 5,3 min; [α]_D_ = −5.575 (0.1 *w*/*v* % in acetone); **^1^H NMR** (600 MHz, acetone-d6) δ (ppm): 8.13–8.10 (2H, m, H-2′, H-6′), 7.24 (1H, d, *J* = 2.5 Hz, H-7), 7.23–7.20 (2H, m, H-3′, H-5′), 7.10 (1H, d, *J* = 2.5 Hz, H-5), 6.77 (1H, s, H-3), 5.66 (2H, s, -NH_2_), 5.08 (1H, d, *J* = 7.8 Hz, H-1″), 4.76 (1H, d, *J* = 4.3 Hz, 2″-OH), 4.49 (1H, d, *J* = 4.1 Hz, 3″-OH), 3.87–3.84 (2H, m, H-6″, 6″-OH), 3.73–3.68 (1H, m, H-6″), 3.65 (1H, td, *J* = 9.1, 4.0 Hz, H-3″), 3.57 (3H, s, 4″-O-CH_3_), 3.54 (1H, ddd, *J* = 9.8, 5.0, 1.9 Hz, H-5″), 3.52–3.50 (1H, m, H-2″), 3.25–3.20 (1H, m, H-4″); **^13^C NMR** (acetone-d6) δ (ppm): 185.96 (C-4), 170.66 (C-2), 157.68 (C-4′), 155.53 (C-8a), 141.71 (C-8), 138.12 (C-1′), 130.01 (C-4a), 129.08 (C-2′, C-6′), 128.34 (C-6), 117.59 (C-3′, C-5′), 116.81 (C-5), 111.13 (C-7), 106.67 (C-3), 101.24 (C-1″), 80.08 (C-4″), 77.94 (C-3″), 77.22 (C-5″), 74.86 (C-2″), 62.05 (C-6″), 60.59 (4″-O-CH_3_)

6-bromo-8-nitroflavone 4′-*O-β*-D-(4″-*O*-methyl)-glucopyranoside (**9a**). C_22_H_20_BrNO_10_, mp: 125–127 °C; HPLC Rt = 8.857 min; [α]_D_ = −12.276 (0.5 *w*/*v* % in acetone); **^1^H NMR** (600 MHz, acetone-d6) δ (ppm): 8.64 (1H, d, *J* = 2.5 Hz, H-7), 8.50 (1H, d, *J* = 2.5 Hz, H-5), 8.11–8.08 (2H, m, H-2′, H-6′), 7.30–7.27 (2H, m, H-3′, H-5′), 7.00 (1H, s, H-3), 5.12 (1H, d, *J* = 7.8 Hz, H-1″), 4.79 (1H, d, *J* = 3.3 Hz, 2″-OH), 4.50 (1H, d, *J* = 3.5 Hz, 3″-OH), 3.89–3.87 (1H, m, H-6″), 3.86–3.85 (1H, m, 6″-OH), 3.72–3.68 (1H, m, H-6″), 3.67–3.64 (1H, m, H-3″), 3.57 (3H, s, -4″-O-CH_3_), 3.57–3.56 (1H, m, H-5″), 3.52–3.51 (1H, m, H-2″), 3.26–3.22 (1H, m, H-4″); **^13^C NMR** (acetone-d6) δ (ppm): 174.92 (C-4), 164.28 (C-2), 162.04 (C-4′), 148.51 (C-8a), 140.58 (C-8), 134.10 (C-5), 133.25 (C-7), 129.35 (C-2′, C-6′), 127.97 (C-4a), 124.88 (C-1′), 117.84 (C-3′, C-5′), 117.18 (C-6), 106.85 (C-3), 101.12 (C-1″), 80.02 (C-4″), 77.95 (C-3″), 77.20 (C-5″), 74.84 (C-2″), 62.01 (C-6″), 60.59 (4″-O-CH_3_).

#### 2.2.1. Microbial Transformation of 6-Chloro-8-Nitroflavone (**6**) in the Culture of *Beauveria bassiana* KCH J1.5

**Figure 2:**
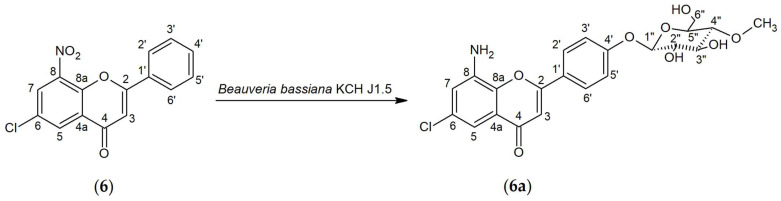
The biotransformation process of 6-chloro-8-nitroflavone (**6**).

The biotransformation of 6-chloro-8-nitroflavone (**6**) was conducted using the *Beauveria bassiana* KCH J1.5 strain. The result of the reaction was the addition of an *O*-methylated glycosidic unit at the C-4′ position and the reduction of the nitro group C-8-NO_2_ to the amino group C-8-NH_2_, which gave the product 8-amino-6-chloroflavone 4′-*O-β*-D-(4″-*O*-methyl)-glucopyranoside (**6a**) (Figure 2). The biotransformation process lasted 7 days, and 4.6 mg of product was obtained (yield 6.02%).

The structure of 8-amino-6-chloroflavone 4′-*O-β*-D-(4″-*O*-methyl)-glucopyranoside (**6a**) was determined based on NMR spectroscopy and LC-MS analysis (Supplementary Materials Figures S42–S55). The presence of a signal from carbon C-1″ at δ = 101.24 ppm in ^13^C NMR spectrum coupled (HMQC) with the characteristic doublet coming from proton H-1″ in the ^1^H NMR spectrum at δ = 5.08 ppm with the coupling constant *J* = 7.8 Hz points to a β-anomer of the attached glucose molecule (Supplementary Materials Figures S45 and S48). In the ^1^H NMR, the characteristic AA’BB’ coupling system is visible with the signals from protons at carbons C-2′ and C-6′ and signals from protons at carbons C-3′ and C-5′, which confirms the para substitution in ring B of the glycosidic molecule (Supplementary Materials Figure S50). Moreover, the glucose is *O*-methylated in position C-4″ (Supplementary Materials Figure S45). A signal from the three protons from the -O-CH_3_ group located at δ = 3.57 ppm (^1^H NMR) correlates with the proton from carbon C-4″ (δ = 3.22 ppm, ^1^H NMR) in the COSY spectrum (Supplementary Materials Figure S51) The carbonyl group remained intact, evidencing the characteristic signal at the ^13^C NMR at δ = 185.96 ppm (Supplementary Materials Figure S47). The nitro group located at carbon C-8 was converted to an amino group. Such a transformation is evidenced by shifts of protons at carbons C-5 (from 8.36 ppm, *J* = 2.7 Hz to 7.10 ppm, *J* = 2.5 HZ) and C-7 (from δ = 8.55 ppm, *J* = 2.7 Hz to δ = 7.24 ppm *J* = 2.5 Hz) relative to the aglycone (chemical shifts characteristic of the presence of an amino group). Also, in the ^1^H NMR spectrum (Supplementary Materials Figure S45), a singlet coming from two protons of the amino group at 5.66 ppm is visible. The mass of the obtained compound was confirmed by LC-MS analysis, which is consistent with the presented structure of the compound containing an amino group (in the C-8 position) and a 4-*O*-methylglucose unit (in the C-4 position).

#### 2.2.2. Microbial Transformation of 6-Bromo-8-Nitroflavone (**9**) in the Culture of *Beauveria bassiana* KCH J1.5

**Figure 3:**
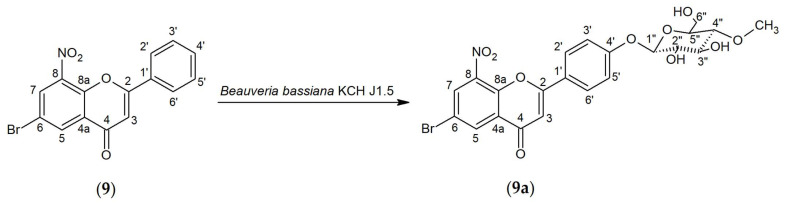
The biotransformation process of 6-bromo-8-nitroflavone (**9**).

The biotransformation of 6-bromo-8-nitroflavone (**9**) was carried out using the *Beauveria bassiana* KCH J1.5 strain. The reaction resulted in the addition of a methylated glycosidic unit at the C-4′ position, which gave the product 6-bromo-8-nitroflavone 4′-*O-β*-D-(4″-*O*-methyl)-glucopyranoside (**9a**) (Figure 3). The biotransformation process lasted 4 days, and 4.3 mg of product was obtained (yield 5.53%).

The obtained product (**9a**) was an *O*-methylglycosylated 6-bromo-8-nitroflavone derivative. The performed NMR and LC-MS analyses confirm the structure and mass of the obtained product, as presented in Supplementary Materials Figures S100–S114. The characteristic signals from the attached glucose molecule were shown in the ^1^H NMR and ^13^C NMR spectra (Supplementary Materials Figures S102 and S105). Confirmation of the glucose linkage to 6-bromo-8-nitroflavone (**9**) was established by the presence of a doublet (proton H-1″) at the anomeric carbon C-1″ at δ = 5.12 ppm in the ^1^H NMR spectrum, with a characteristic coupling constant *J* = 7.8 Hz (Supplementary Materials Figure S102). A singlet coming from three protons at δ = 3.57 ppm in the ^1^H NMR and the signal at δ = 60.59 ppm in the ^13^C NMR spectrum proves the presence of a -O-CH_3_ group. Moreover, this signal correlated in the HMBC spectrum with the C-4″ signal at δ = 80.02 ppm, which proves the *O*-methylation at the carbon C-4″ hydroxyl group of the glucose (Supplementary Materials Figure S114). The presence of the characteristic signals AA’BB’ coupling system in the flavonoid B ring from protons at carbons C-2′ and C-6′ and from the carbons C-3′ and C-5′ confirms the substitution at carbon C-4′ (Supplementary Materials Figure S107). The HMBC spectrum shows the coupling of the signal from a proton H-1″ (δ = 5.12 ppm) derived from sugar, with the signal coming from carbon C-4′ (δ = 162.04 ppm) coming from the flavonoid B-ring, proving substitution with the glucose molecule at this position (Supplementary Materials Figure S113). The multiplicity and chemical shifts of the signals from protons H-5 and H-7 in the ^1^H NMR spectrum (Supplementary Materials Figure S101) and chemical shifts of the signals in the ^13^C NMR from A-ring carbons confirm the preservation of the arrangement of substituents at the C-6 and C-8 position as in the substrate (**9**).

### 2.3. Antimicrobial Analysis of Compounds (**4**–**9**)

**Figure 4:**
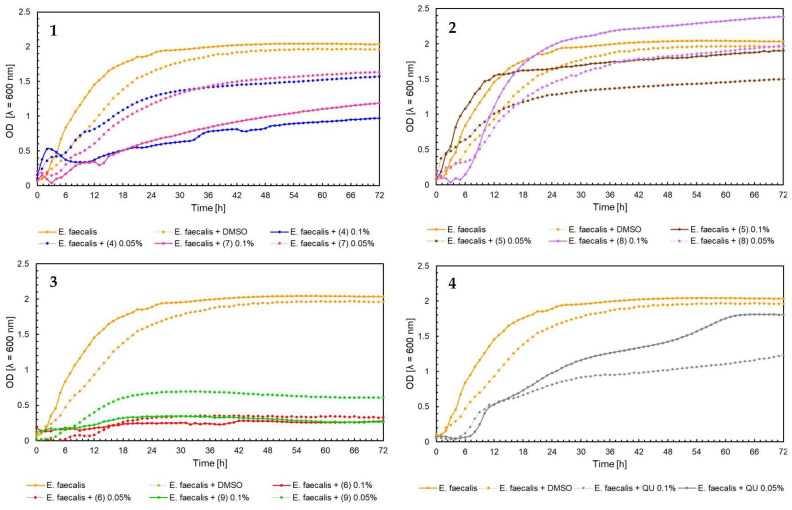
The growth kinetics of *E. faecalis* ATCC 19433 under the effects of flavonoids (**4**–**9**) and quercetin (**QU**), charts (**1**–**4**).

**Figure 5:**
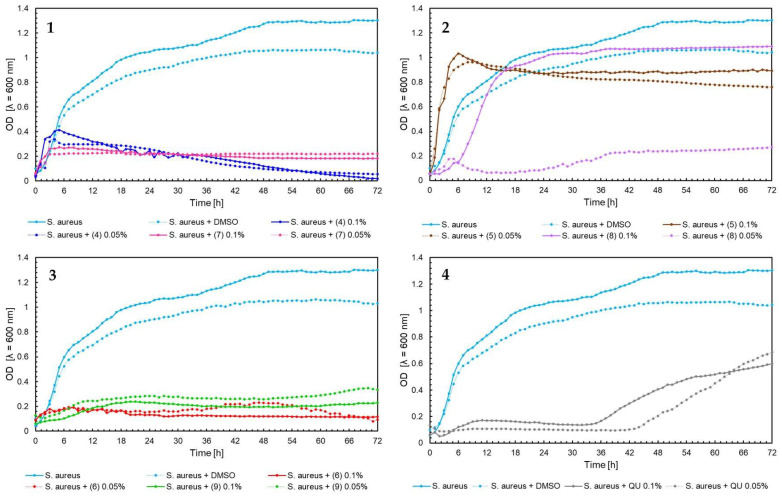
The growth kinetics of *S. aureus* ATCC 29213 under the effects of flavonoids (**4**–**9**) and quercetin (**QU**), charts (**1**–**4**).

**Figure 6:**
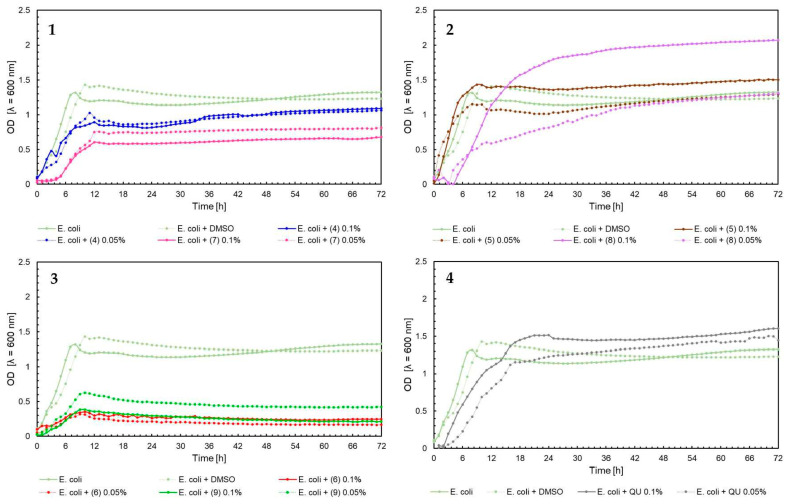
The growth kinetics of *E. coli* ATCC 25922 under the effects of flavonoids (**4**–**9**) and quercetin (**QU**), charts (**1**–**4**).

**Figure 7:**
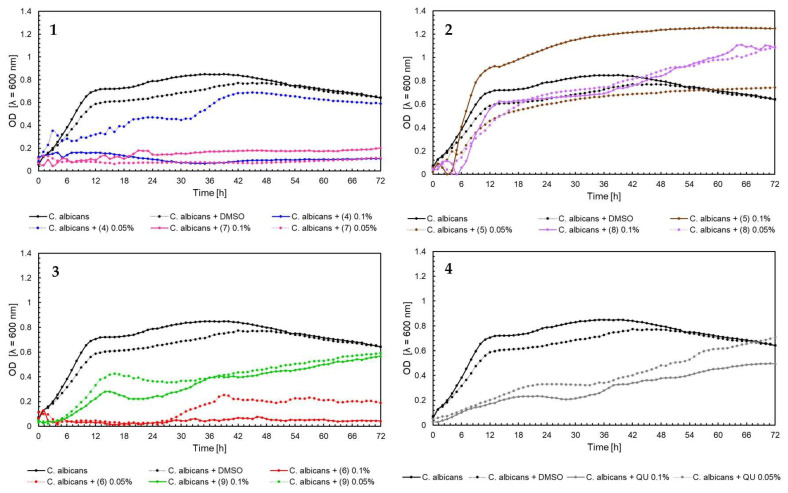
The growth kinetics of *C. albicans* ATCC 10231 under the effects of flavonoids (**4**–**9**) and quercetin (**QU**), charts (**1**–**4**).

**Figure 8:**
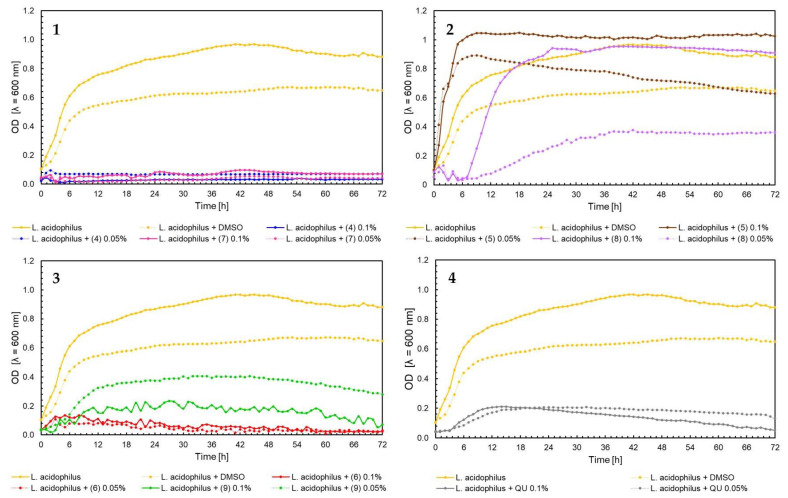
The growth kinetics of *L. acidophilus* ATCC 4356under the effects of flavonoids (**4**–**9**) and quercetin (**QU**), charts (**1**–**4**).

**Figure 9:**
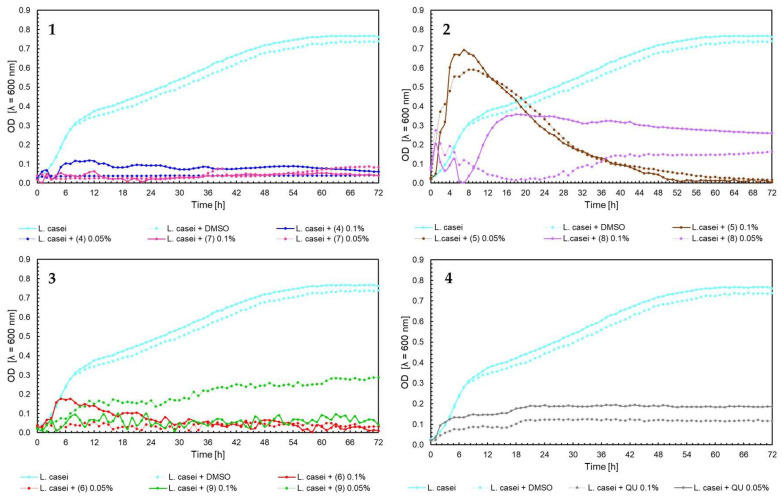
The growth kinetics of *L. casei* ATCC 393 under the effects of flavonoids (**4**–**9**) and quercetin (**QU**), charts (**1**–**4**).

**Figure 10:**
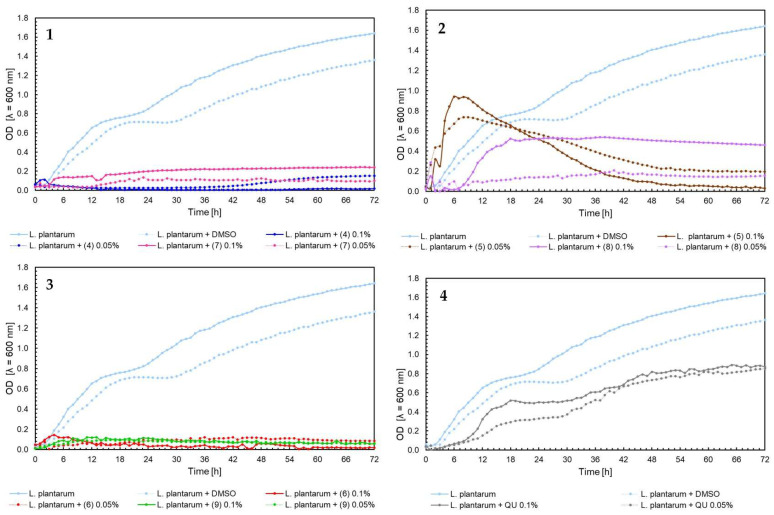
The growth kinetics *L. plantarum* ATCC 14917 under the effects of flavonoids (**4**–**9**) and quercetin (**QU**), charts (**1**–**4**).

**Figure 11:**
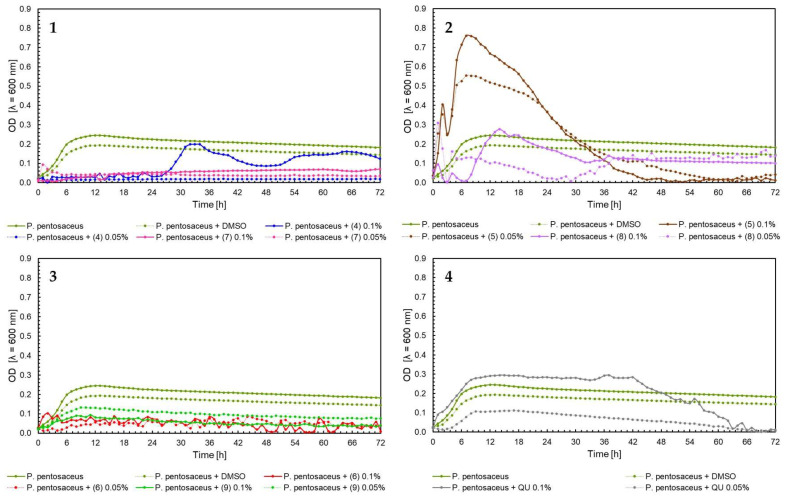
The growth kinetics *P. pentosaceus* ATCC 33316 under the effects of flavonoids (**4**–**9**) and quercetin (**QU**), charts (**1**–**4**).

The effect of 5′-chloro-2′-hydroxy-3′-nitrochalcone (**4**), 5′-bromo-2′-hydroxy-3′-nitrochalcone (**7**), 6-chloro-8-nitroflavanone (**5**), 6-bromo-8-nitroflavanone (**8**), 6-chloro-8-nitroflavone (**6**), 6-bromo-8-nitroflavone (**9**)**,** and quercetin (**QU**) at two concentrations (0.05% and 0.1%) against *E. faecalis* ATCC 19433, *S. aureus* ATCC 29213, *E. coli* ATCC 25922, and *C. albicans* ATCC 10231 and four probiotic *L. acidophilus* ATCC 4356, *L. casei* ATCC 393, *L. plantarum* ATCC 14917, and *P. pentosaceus* ATCC 33316 was tested. Quercetin (**QU**) was used in this research to compare the synthesized new flavonoids with a compound available and used in industry. The obtained results are presented in Figure 4, Figure 5, Figure 6, Figure 7, Figure 8, Figure 9, Figure 10 and Figure 11. The kinetics of changes in optical density, which express the amount of cellular biomass of microorganisms, were assessed over 72 h. All control samples were characterized by growth kinetics typical of a given species. The growth of microorganisms was not disturbed by the presence of undesirable substances/chemical compounds.

The kinetics of optical density changes analysis of *E. faecalis* ATCC 19433 showed that the strongest inhibitory effect on this bacterium had the 6-chloro-8-nitroflavone (**6**) in concentration 0.05% and 0.1%, and 6-bromo-8-nitroflavone (**9**) in concentration 0.1% (Figure 4, chart 3). For these flavonoids, the optical density does not exceed 0.4 value. In contrast, for flavanones (**5**) and (**8**), optical density reaches a value close to 2 (Figure 4, chart 2). It is also worth noting that in the case of chalcones (**4**) and (**7**), a higher concentration of the compound of 0.1% inhibited the growth of *E. faecalis* more than a lower concentration of 0.05% (Figure 4, chart 1). It can also be noted that the addition of quercetin (**QU**) would not significantly affect *E. faecalis* growth (Figure 4, chart 4).

In the case of *S. aureus* ATCC 29213, flavone (6) in concentrations of 0.05% and 0.1% and flavone (9) in 0.1% concentration (Figure 5, chart 3) observed a similar result to that for *E. faecalis*. However, the changes in the optical density of the *S. aureus* strain in the presence of other compounds were also interesting. For chalcone (4) in both concentrations, initially, for 3 h, the growth of microorganisms was observed to approximately 0.4 optical density. Then, a linear reduction in the density of cell biomass was noticed, which was probably the effect of cell autolysis (Figure 5, chart 1). In the case of quercetin (QU), no microbial growth was observed for the first 36 h. After this time, the increasing optical density of microorganism biomass was observed (OD = 0.5–0.7). The optical density growth lasted until the end of the experiment, up to 72 h (Figure 5, chart 4). This state could be related to the prolonged adaptation phase of microorganisms in the medium containing quercetin (**QU**).

In the case of *E. coli* ATCC 25922, the kinetics of optical density changes were different compared to the examples described above for *S. aureus* and *E. faecalis*, while in the case of compounds (6) 0.05%, (6) 0.1%, and (9) 0.1%, an inhibition of the growth of *E. coli* was observed (Figure 6, chart 3). In the case of flavanones (5) 0.1%, (8) 0.1%, and quercetin (**QU**) 0.1% (Figure 6, charts 2 and 4), stimulation of the growth of microorganisms was observed, which was confirmed by a higher optical density of the tested flavonoids compared to bacterial control. It can also be noted that (**QU**) would not significantly affect the *E. coli* growth. Similar relationships were observed for the yeast *C. albicans* ATCC 10231 (Figure 7, charts 1–4). For compound (5) 0.1%, the maximum optical density was 1.6 compared to the yeast control, in which the maximum OD averaged 0.8. A strong inhibitory effect on *C. albicans* was observed for compounds (4) 0.1%, (6) 0.05%, (6) 0.1%, (7) 0.05% and (7) 0.1%. In these cases, the optical density was equal to or lower than the baseline level (time 0 h—start of the experiment).

In the case of probiotic bacterial species, the nature of microbial growth can be very diverse. For the *L. acidophilus* ATCC 4356 strain, strong growth-inhibiting properties were demonstrated for flavone (**6**) at both 0.05% and 0.1% concentrations (Figure 8, chart 3), as well as for chalcones (**4**), (**7**), and quercetin (**QU**) (Figure 8, charts 1 and 4). Stimulation of the growth of the probiotic strain was demonstrated for flavanones (**5**) and (**8**) at concentration of 0.1% (Figure 8, chart 2).

The kinetics of optical density changes for *L. casei* ATCC 393 (Figure 9, charts 1–4) were similar to those in the case of *L. acidophilus*. Particularly, a strong inhibitory effect on *L. casei* bacteria was caused by chalcones (**4**) and (**7**), flavone (**6**) and quercetin (**QU**) in both concentrations 0.1% and 0.05%, and for flavone (**9**) in concentration 0.1%. The wide range of changes in bacteria growth were observed for 6-chloro-8-nitroflavanone (**5**) in both concentrations. At the beginning of the experiment, a high optical density (0.7) was observed; next, after 8 h of the experiment, the optical density of bacteria fell significantly to 0 in 48 h.

In the case of *L. plantarum* ATCC 14917 strain, the growth of bacteria was not observed under any of tested flavonoids (Figure 10, charts 1–4). However, the strongest antimicrobial properties were observed for chalcones (**4**), (**7**), and flavones (**6**), (**9**) in both concentrations 0.05% and 0.1%. The weaker inhibitory effect showed quercetin (**QU**), which achieved optical density for *L. plantarum* around a value of 0.8, in contrary to the native bacteria, for which maximum growth was 1.6.

In the case of *P. pentosaceus* ATCC 33316, bacteria growth inhibition was observed for chalcones (7) and (4) in 0.05% and 0.1% and flavones (6) and (9) in the same concentrations (Figure 11, charts 1 and 3). Contrary to the growth of *P. pentosaceus* with the addition of flavanones, for the first 7 h of the experiment, the optical density of bacteria with 6-chloro-8-nitroflavanone (5) grew and reached a peak of over 0.7 for the concentration 0.1%, and over 0.5 for the concertation 0.05%. After this, there was a sudden decrease to 0–0.1 OD value in the 48 h of the experiment. In comparison to the 6-bromo-8-nitroflavanone (8) in both concentrations, the data fluctuated, and after 48 h of the experiment, the data remained stable at 0.1–0.13 OD value (Figure 11, chart 2). In Figure 9 chart 2, Figure 10 chart 2, and Figure 11 chart 2, a similar bacterial growth pattern in the presence of flavanone (**5**) can be noted. Initially, there is an upsurge in bacterial optical density for approximately the first 8 h of the experiment, followed by a decline until the 48 h mark, with values nearing zero in the last 24 h. We propose that, in this scenario, the formation of toxic or detrimental compounds, potentially biotransformation products created by these bacteria after approximately 8 h of the experiment, could be responsible. This rapid biotransformation process of flavanone may also lead to a faster reduction in nutrients in the medium, including depletion of the carbon source. However, confirming these hypotheses would necessitate further comprehensive investigation. In the case of quercetin (**QU**), at up to 42 h of the experiment, the optical density of *P. pentosaceus* was around 0.3; these values went down and reached 0 within 72 h of the test (Figure 11, chart 4).

## 3. Summary and Discussion

The main aim of the research was to assess the antimicrobial activity of flavonoids with bromine or chlorine and nitro group against pathogenic and probiotic bacteria occurring in the human body (in the intestines). The research aimed to discover the properties of flavonoids that had not been previously described in the literature and to select the most active compound. The next goal was to create flavonoid glycosides using entomopathogenic filamentous fungi in the biotransformation process. Therefore, we have expanded the library with new glycoside derivatives of flavonoids with -Br, -Cl and -NO_2_.

Referring to the first stage of research, which was the biotransformation of previously synthesized flavonoid compounds, entomopathogenic filamentous fungi were able to transform just flavones (**6**) and (**9**). In both cases, only *B. bassiana* KCH J1.5 was able to conduct microbial transformation. In the case of 6-chloro-8-nitroflavone (**6**), the glycosidic unit was attached at the C-4′ position, and the -NO_2_ group was reduced to -NH_2_. As for 6-bromo-8-nitroflavone (**9**), only the glucose molecule was attached at the C-4′ position. In previous work of our team, we also noted the ability of filamentous fungi to the microbial transformation of flavones [11,24,25]. We noted that, in the case of 6-methyl-8-nitroflavon, only *B. bassiana* KCH J1.5 was able to glycosylate the compound in the position C-4′. However, in the case of 8-bromo-6-chloroflavone, glycosylation occurred only in the culture of the *I. farinosa* KCH J2.6 strain also in the position C-4′ [26].

Focusing on glycosylation in the *B. bassiana* strain culture, there are many papers describing this ability. Zeng et al. reported the first glycosylation of curcumin by the *B. bassiana* strain ATCC 7159 as the whole-cell biocatalyst. Thanks to this transformation, the compound showed increased solubility in water and higher antibacterial activity against *S. aureus* and *E. coli* [27]. Other scientists proved that *B. bassiana* AM278 can attach 4-*O*-methyl glucopyranose to quercetin in the C-7 position with a highly regioselective reaction. The obtained quercetin 7-*O*-β-D-(4″-*O*-methyl)glucopyranoside showed much more limited interaction with the lipid membrane since the sugar moiety abolished hydrophobicity and rendered this molecule larger. They also showed that quercetin glucoside may be responsible for inhibiting lipid peroxidation by hindering the diffusion of free radicals into the membrane and reducing the efficiency of their reaction [26]. Biotransformation studies in cultures of *B. bassiana* KCH J1.5 and BBT strains have shown various possibilities in flavonoid glycosylation. Scientists have proven that these strains can biotransform flavokawain B into four different glycoside derivatives. They also tested another strain of *Beauveria bassiana* KCH J1, which, unlike the previous two, was not able to transform flavokawain B into glycoside derivatives [28].

Summarizing our investigations, it can be noticed that in both these studies and those described in our previous work [29], glycosylation of flavones only occurs at the C-4′ position, regardless of the type of substituents in the A ring. This may be because the B ring of the flavonoid is not substituted with additional groups, which may result in less electronic hindrance and the attachment of the glycosidic unit. Our research confirms the ability of the *B. bassiana* KCH J.5 strain to glycosylation of flavonoids, in particular flavones with halogen atoms and nitro or methyl groups. This proves the high “flexibility” of the enzymatic center of the entomopathogenic filamentous fungus to accept various structures of flavonoid compounds with different substituents. Our research shows great potential for using *B. bassiana* to obtain new glycoside derivatives of flavonoids, which in the future can be used in the food, pharmaceutical, and cosmetic industries.

In the case of research on the properties of obtained chalcones, flavanones and flavones, our investigations showed that most of the tested compounds exhibited moderate to high antibacterial activity against selected microorganisms. Flavones and, to a great extent, chalcones were found to induce the strongest inhibitory effect of all bacterial and yeast growth compared to flavanones.

In the case of pathogenic bacteria *E. faecalis* ATCC 19433, *S. aureus* ATCC 29213, *E. coli* ATCC 25922, and yeast *C. albicans* ATCC 10231, the strongest inhibitory effect had 6-chloro-8-nitroflavone (**6**) in both concentrations 0.05% and 0.1%. The 6-bromo-8-nitroflavone (**9**) in both concentrations also inhibited the growth of microorganisms. Similar effects for chalcones (**4**) and (**7**) were observed. A stronger inhibitory effect was shown in 5′-chloro-2′-hydroxy-3′-nitrochalcone (**4**), except against *E. coli*, where chalcone (**7**) with a bromine atom performed better. For *C. albicans*, the 0.05% concentration of compound (**4**) did not significantly influence yeast growth. In contrary, flavanones (**5**) and (**8**) did not substantially affect the growth of pathogenic microorganisms.

Moving on to the influence of flavonoids on probiotic bacteria *L. acidophilus* ATCC 4356, *L. casei* ATCC 393, *L. plantarum* ATCC 14917, *P. pentosaceus* ATCC 33316, the best results were for flavanones (**5**) and (**8**). The compounds showed even the induction of the growth of *L. acidophilus* and *P. pentosaceus* (Figure 8 and Figure 11). They were also less harmful than quercetin (**QU**), which is used commercially. In relation to chalcones, the inhibition of the growth of probiotic bacteria in both concentrations was noticed. Flavones also reduced the growth of tested bacteria. It can be concluded that flavanones have the potential for further use as promising ingredients in the pharmaceutical industry. They do not negatively affect the tested strains of probiotic bacteria found in the human digestive system. This is extremely important if the given compounds are to be used as substances that help rebuild the intestinal microflora. This research is intended to serve as a basic screening of the selected flavonoids, complement the literature about them, and allow the research community to plan future experiments. The presented properties of flavonoids also allow focus on several of the most active substances and the extension of biological research. Of course, further studies and knowledge of more properties of these compounds are required to determine their exact effects and use.

Our studies showed that halogenated compounds are more active molecules against bacteria and yeast than non-halogenated ones like quercetin. The research presented by Thebti et al. also agrees with this thesis. Moreover, scientists claimed that chalcones had a higher inhibitory effect on bacterial growth [12]. In our study, we observed similar relationships. The highest inhibition of bacterial growth showed flavones and chalcones, and the lowest showed flavanones. Other studies presented by Liu et al. showed that the most effective inhibitors for the influenza virus from various classes of flavonoids were aurones > flavon(ol)es > isoflavones > flavanon(ol)es and flavan(ol)es. They also confirm that the presence of 4′-OH, 7-OH, C4=O, and C2=C3 functionalities were essential for the inhibition of the virus [30].

Many studies report that antimicrobial activity is strongly associated with substituents in the B ring of flavonoid compounds. These substituents affected the activity of the compound regardless of how the A ring was substituted [15,31,32]. So far, we can find many mechanisms in the literature suggesting the antibacterial effect of flavonoids [33]. The most common is damage to the cytoplasmic membrane caused by perforation [34] or decreased membrane fluidity [35], inhibition of nucleic acid synthesis [36], and inhibition of energy metabolism caused by inhibition of NADH-cytochrome C reductase [37]. Moreover, the number of studies on flavonoids is constantly increasing to such an extent that the activity of some compounds, including quercetin [38,39,40], has been repeatedly studied, and numerous mechanisms have been attributed to it. Other studies suggest that the cell membrane is the main site of flavonoids acting on Gram-positive bacteria, probably related to damage of phospholipid bilayers, the inhibition of the respiratory chain, or ATP synthesis [41]. More research focusing on the structure–activity relationship is needed to determine a mechanism for the antibacterial action of flavonoids.

## 4. Materials and Methods

### 4.1. Substrates

2′-hydroxy-5′-methyl-3′-nitroacetophenone (**1**), 3′-bromo-5′-chloro-2′-hydroxyacetophenone (**2**), and benzaldehyde (**3**) were purchased from Sigma-Aldrich (St. Louis, MO, USA).

The substrates 5′-chloro-2′-hydroxy-3′-nitrochalcone (**4**), 5′-bromo-2′-hydroxy-3′-nitrochalcone (**7**), 6-chloro-8-nitroflavanone (**5**), 6-bromo-8-nitroflavanone (**8**), 6-chloro-8-nitroflavone (**6**), and 6-bromo-8-nitroflavone (**9**) were synthesized according to the reaction presented in Figure 1.

The initial stage involved synthesizing chalcones (**4**) and (**7**) through the Claisen–Schmidt condensation, as illustrated in Figure 1. The reaction of appropriately substituted acetophenone (**1**) and (**2**) with benzaldehyde (**3**) resulted in the production of 5′-chloro-2′-hydroxy-3′-nitrochalcone (**4**) (83.02% yield) and 5′-bromo-2′-hydroxy-3′-nitrochalcone (**7**) (85.92% yield). The substrates for the synthesis were dissolved in methanol under alkaline conditions (NaOH) with the addition of water. The reaction proceeded for 3 h at the boiling point of the reactants under reflux. The next synthesis involved cyclizing chalcones (**4**) and (**7**) into flavanones: 6-chloro-8-nitroflavanone (**5**) (0.55% yield) and 6-bromo-8-nitroflavanone (**8**) (0.83% yield) using sodium acetate dissolved in methanol under reflux for 24 h (Figure 1). The final reaction aimed to obtain flavones (**6**) (53.06% yield) and (**9**) (97.74% yield) by reacting appropriate chalcones with iodine (I_2_) dissolved in dimethyl sulfoxide (DMSO) for 3 h under reflux at 125 °C in an oil bath (Figure 1).

Obtained products were analyzed structurally with the use of nuclear magnetic resonance (NMR), and masses were confirmed by liquid chromatography–mass spectrometry (LC-MS); detailed data are presented in the Supplementary Materials.

### 4.2. Microorganisms

In the biotransformation procedure, three strains of entomopathogenic filamentous fungi, namely *Beauveria bassiana* KCH J1.5, *Isaria fumosorosea* KCH J2, and *I farinosa* KCH J2.6, were applied. These microorganisms are affiliated with the Department of Food Chemistry and Biocatalysis at the Wrocław University of Environmental and Life Sciences in Poland. The techniques for isolating entomopathogenic filamentous fungi, their reproduction, and genetic identification have been detailed in our earlier publications [42,43,44].

In the antimicrobial activity studies, four pathogenic *Enterococcus faecalis* ATCC 19433, *Staphylococcus aureus* ATCC 29213, *Escherichia coli* ATCC 25922, and *Candida albicans* ATCC 10231 and four probiotic *Lactobacillus acidophilus* ATCC 4356, *Lactobacillus casei* ATCC 393, *Lactobacillus plantarum* ATCC 14917, and *Pediococcus pentosaceus* ATCC 33316 microorganisms were used. These microorganisms were purchased from American Type Culture Collection from United States. Species of microorganisms were selected for the study, including both pathogenic and probiotic strains that are closely related to mammals. This choice fits into the broader context of our team’s research, especially in relation to the potential applications of the studied groups of compounds.

### 4.3. Chemical Analysis

Analytical and preparative thin-layer chromatography (TLC) was used to evaluate the progression of synthesis, biotransformation, and the isolation of products. Analytical TLC was utilized to track the course of chemical syntheses and biotransformation. Specifically, TLC Silica gel 60/Kieselguhr F254 aluminum sheets measuring 20 cm × 20 cm with a thickness of 0.2 mm (Merck, Darmstadt, Germany) were used. For chemical synthesis analysis, the eluent consisted of a 9:1 and 4:1 *v*/*v* ratio of cyclohexane (Chempur, Piekary Śląskie, Poland) to ethyl acetate (Chempur, Piekary Śląskie, Poland). Meanwhile, a mixture of chloroform (Chempur, Piekary Śląskie, Poland) to methanol (Chempur, Piekary Śląskie, Poland) in a 9:1 *v*/*v* ratio was used for monitoring biotransformation progress. In both cases, observations were conducted under a UV lamp with wavelengths of λ = 254 nm and λ = 365 nm. Preparative TLC was utilized for the separation of the product mixture during scale-up biotransformation. Preparative TLC Silica plates from Analtech, Gehrden, Germany (with thicknesses of 0.5, 1, and 2 mm) were used with an eluent composed of chloroform (Chempur, Piekary Śląskie, Poland) and methanol (Chempur, Piekary Śląskie, Poland) in a 9:1 *v*/*v* ratio. The products were observed under a UV lamp with wavelengths of λ = 254 nm and λ = 365 nm. Subsequently, the products underwent extraction thrice using 15 mL of ethyl acetate (Chempur, Piekary Śląskie, Poland), followed by filtration and evaporation in a vacuum evaporator.

HPLC chromatography was conducted to assess the progression of the biotransformation and determine the retention times of both substrates and products. The analysis utilized a Dionex Ultimate 3000 instrument (Thermo Fisher Scientific, Waltham, MA, USA) with a DAD-3000 diode array detector using an analytical octadecylsilica (ODS) 2 column (4.6 mm × 250 mm, Waters, Milford, MA, USA) and a pre-column. The eluent consisted of a mixture of 0.1% aqueous formic acid *v*/*v* (A) and acetonitrile (B). The gradient program encompassed the following steps: initial conditions—32.5% B in A, 4 min—40% B in A, 8 min—40% B in A, 10 min—45% B in A, 15 min—95% B in A, 18 min—95% B in A, 19 min—32.5% B in A, and 23 min—32.5% B in A. The flow rate was 1 mL/min, with an injection volume of 5 µL, and detection at a wavelength of 280 nm [45]. Data were collected using Chromeleon software version 7.2 (Thermo Fisher Scientific, Waltham, MA, USA).

Nuclear Magnetic Resonance (NMR), including ^1^H NMR, ^13^C NMR, COSY, HSQC, and HMBC, was used to analyze the structure of obtained compounds. This was accomplished utilizing the DRX Avance^TM^ 600 MHz NMR spectrometer (Bruker, Billerica, MA, USA). All samples were dissolved in 0.7 mL of deuterated acetone.

The determination of the mass for the obtained biotransformation substrates and products was verified through LC-MS analysis, using an LC-MS 8045 SHIMADZU Triple Quadrupole Liquid Chromatograph Mass Spectrometer equipped with electrospray ionization (ESI) source (Shimadzu, Kyoto, Japan). The analyses employed the “product ion scan” method. Each sample containing a pure compound underwent a search for a specific ion with a known molecular mass, determined through prior NMR analysis. Separation was achieved on a Kinetex column (2.6 µm C18 100 Å, 100 mm × 3 mm, Phenomenex, Torrance, CA, USA) operated at 30 °C. The mobile phase constituted a mixture of 0.1% aqueous formic acid *v*/*v* (A) and acetonitrile (B), with a flow rate of 0.4 mL/min and an injection volume of 5 µL. The gradient program included initial conditions—80% B in A, 6.5 min—100% B, and 7 min—80% B in A. Key operational parameters for the LC-MS were set as follows: nebulizing gas flow: 3 L/min, heating gas flow: 10 L/min, interface temperature: 300 °C, drying gas flow: 10 L/min, data acquisition range: *m*/*z* 100–1000 Da, positive ionization mode. Data were collected using LabSolutions version 5.97 (Shimadzu, Kyoto, Japan).

Optical rotation was measured using digital polarimeter P-2000-Na (ABL&E-JASCO, Kraków, Poland).

### 4.4. Small-Scale Biofransformtion

Small-scale biotransformation utilized three entomopathogenic filamentous fungi: *B. bassiana* KCH J1.5, *I. fumosorosea* KCH J2, and *I. farinosa* KCH J2.6. These fungi have been chosen for their known ability to transform flavonoid compounds, specifically in producing glycoside derivatives. This selection was based on prior screening studies conducted by our team in the Department of Food Chemistry and Biocatalysis [46,47]. This segment of the research aimed to identify the suitable microorganism and optimal biotransformation duration for substrates (**4**), (**6**), (**7**), and (**9**) in preparation for subsequent scale-up studies.

Flavanones (**5**) and (**8**) were not biotransformed on small-scale and scale-up biotransformation, because the low chemical synthesis efficiency does not allow for their use in the biotransformation processes.

The small-scale biotransformation was conducted in 300 mL flat-bottomed conical flasks (Erlenmeyer flasks) containing 100 mL of modified Sabouraud medium (1% aminobac, 3% sucrose per 1 L of water). Each flask was inoculated with approximately 1 mL of an entomopathogenic filamentous fungi culture and shaken for 72 h at 140 rpm and 25 °C. Next, 10 mg of the substrate was added into each flask, and the mixture was shaken again at 140 rpm at 25 °C. Sampling occurred after 3, 7, and 10 days of the experiment. After collection, the samples underwent extraction with ethyl acetate in a 1:1 ratio of ethyl acetate to medium, and the extracts were collected in separate flasks. The extracts were dried using magnesium sulfate (MgSO_4_), filtered, and concentrated in a vacuum evaporator. Samples for HPLC analysis were prepared from the obtained extracts. The dried extracts were dissolved in 1 mL of acetonitrile and subjected to HPLC analysis. The outcomes of this analysis allowed us to identify the optimal biotransformation time and strain for scale-up experiment, aiming for maximum efficiency in the process.

### 4.5. Scale-Up Biotransformation

The upscaling of biotransformation was carried out in a 2 L flask containing 500 mL of modified Sabouraud’s medium (consistent with the formulation used in small-scale experiments). The objective of the scale-up biotransformation was to obtain a larger quantity of products for further analysis.

To the flask containing the sterile medium, 1 mL of a preincubation culture of entomopathogenic filamentous fungi was added. Next, the flask underwent incubation for 72 h at 25 °C with shaking at 145 rpm. After this incubation period, 50 mg of substrate, dissolved in 2 mL of DMSO, was added to the flask. The duration of the scale-up biotransformation was determined based on prior small-scale studies involving the specific microorganism and substrate. The subsequent step involved extracting the obtained products through three cycles using 350 mL of ethyl acetate every time. The combined extracts were then dried with MgSO_4_, filtered, and evaporated under vacuum. The resulting samples were subjected to separation using preparative TLC plates. The extract was dissolved in approximately 2 mL of THF, and the substance application on the plate occurred using a semi-automatic sample dispenser (Linomat 5, CAMAG, Muttenz, Switzerland). The sample was sprayed on bandwise under pressure using a dedicated dosing syringe with a volume of 500 μL. After that, the TLC plates were put in a chromatography chamber, and product separation occurred using a chloroform to methanol mixture (9:1, *v*/*v*) as the eluent. Visualization of the TLC plates was carried out under a UV lamp (254 nm and 365 nm), and separated fractions were isolated from the plate. These fractions were extracted thrice with 15 mL of ethyl acetate for 30 min each. The resulting extracts were filtered and evaporated to dryness using a vacuum evaporator. Then, the prepared fractions were dissolved in 0.7 mL of deuterated acetone and subjected to NMR analysis.

NMR analyses are presented in Supplementary Materials.

### 4.6. Antimicrobial Studies

Antimicrobial tests consisted of checking the activity of flavonoid compounds (**4**–**9**) on the impact of the growth of selected microorganisms. The research aimed to find out the antimicrobial properties of compounds undescribed in the literature and to select the most active substance.

For antimicrobial analysis, in 50 mL conical tubes, *E. faecalis* ATCC 19433, *S. aureus* ATCC 29213, *E. coli* ATCC 25922, *C. albicans* ATCC 10231, *L. acidophilus* ATCC 4356, *L. casei* ATCC 393, *L. plantarum* ATCC 14917, and *P. pentosaceus* ATCC 33316 were prepared. Each tube contained 25 mL of Mueller–Hinton broth. Conical tubes were inoculated with approximately 1 mL of microorganisms and shaken for 48 h at 120 rpm at 37 °C, except for *C. albicans*—30 °C.

Flavonoid compounds (**4**–**9**) were dissolved in DMSO at concentrations of 0.1% and 0.05%. The analysis was conducted on a Synergy H1 microplate reader (BioTek Instruments, Winooski, VT, USA). The tests were carried out on a 96-well microplate continuous assay for 72 h, with absorbance measurements (OD600) every 1 h and shaking breaks before each measurement (10 s, 282 cpm). The working volume in each well was 300 µL, which included Mueller–Hinton broth, 50 µL of the microorganism, and 10 µL of dissolved flavonoid at the appropriate concentration. Imager Software Gen5 version 3.11 was used for data collection and analysis.

## 5. Conclusions

Synthesis of flavonoids via Claisen–Schmidt condensation and their further biotransformation using entomopathogenic filamentous fungi yielded two novel glycoside derivatives, namely 8-amino-6-chloroflavone 4′-*O-β*-D-(4″-*O*-methyl)-glucopyranoside (**6a**) and 6-bromo-8-nitroflavone 4′-*O*-*β*-D-(4″-*O*-methyl)-glucopyranoside (**9a**).

Investigations of the microbial activity of the synthesized flavonoids revealed that flavones, as well as chalcones, exhibited superior inhibitory effects compared flavanones against pathogenic and probiotic bacteria. In particular, 6-chloro-8-nitroflavone (**6**) demonstrated potent inhibitory activity against pathogenic bacteria, highlighting its potential as a therapeutic agent.

Interestingly, flavanones such as 6-chloro-8-nitroflavanone (**5**) and 6-bromo-8-nitroflavanone (**8**) stimulated the growth of some probiotic bacteria, specifically *L. acidophilus* and *P. pentosaceus*. This finding suggests a potential role for flavanones in promoting beneficial microbial growth in the human body.

Overall, the study contributes to the understanding of the antimicrobial properties of flavonoids and highlights their potential as candidates for the development of novel therapeutic agents to combat bacterial infections and promote human health. Further investigations are needed to explore the broader spectrum of their biological activities and therapeutic applications.

## Data Availability

Samples of the compounds (**1**–**9**), (**6a**), and (**9a**) are available from the authors.

## Supplementary Materials

The following supporting information can be downloaded at: https://www.mdpi.com/article/10.3390/ijms25105540/s1.
