# Supplementary material for: Antimicrobial Properties of Flavonoid Derivatives with Bromine, Chlorine, and Nitro Group Obtained by Chemical Synthesis and Biotransformation Studies
Source: Int J Mol Sci. 2024 May 19;25(10):5540. doi: 10.3390/ijms25105540 (PMC11122099; doi:10.3390/ijms25105540)
Supplement: Supplementary file 1 [file ijms-25-05540-s001.zip › ijms-2989965-supplementary.pdf]

Supplementary Materials

Antimicrobial Properties of Flavonoid Derivatives with Bromine, Chlorine, and Nitro Group Obtained by Chemical Synthesis and Biotransformation Studies

Martyna Perz[1,*], Daria Szymanowska[2,3], Tomasz Janeczko[1], and Edyta Kostrzewa-Susłow[1,*]

[1] Department of Food Chemistry and Biocatalysis, Faculty of Biotechnology and Food Science, Wrocław University of Environmental and Life Sciences, 50-375 Wrocław, Poland;

[2] Department of Biotechnology and Food Microbiology, Faculty of Food Science and Nutrition, Poznań University of Life Sciences, 60-627 Poznan, Poland;

[3] Department of Pharmacognosy and Biomaterials, Faculty of Pharmacy, Poznań University of Medical Sciences, Rokietnicka 3, 60-806 Poznań, Poland;

* Correspondence: martyna.perz@upwr.edu.pl (M.P.); edyta.kostrzewa-suslow@upwr.edu.pl (E.K.-S.)

Compound name: 5′-chloro-2′-hydroxy-3′-nitrochalcone
Molecular Formula: $C_{15}H_{10}ClNO_4$
Formula Weight: 303.697
Ionization mode: positive
Precursor $[M + H]^+$: 304.029
Monoisotopic Mass: 303.029
Collision energy (CE): -15.0

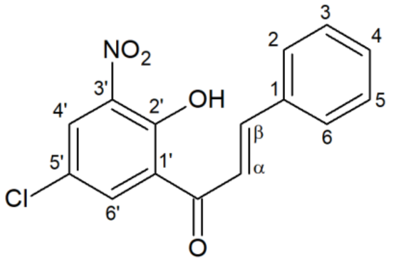

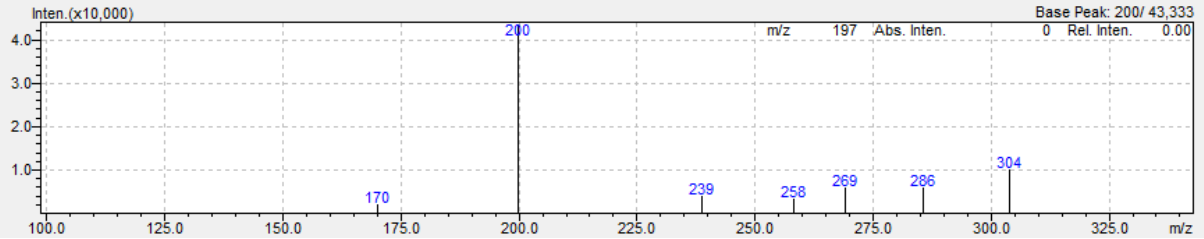

Figure S1. LC-MS spectrum of 5′-chloro-2′-hydroxy-3′-nitrochalcone (4).

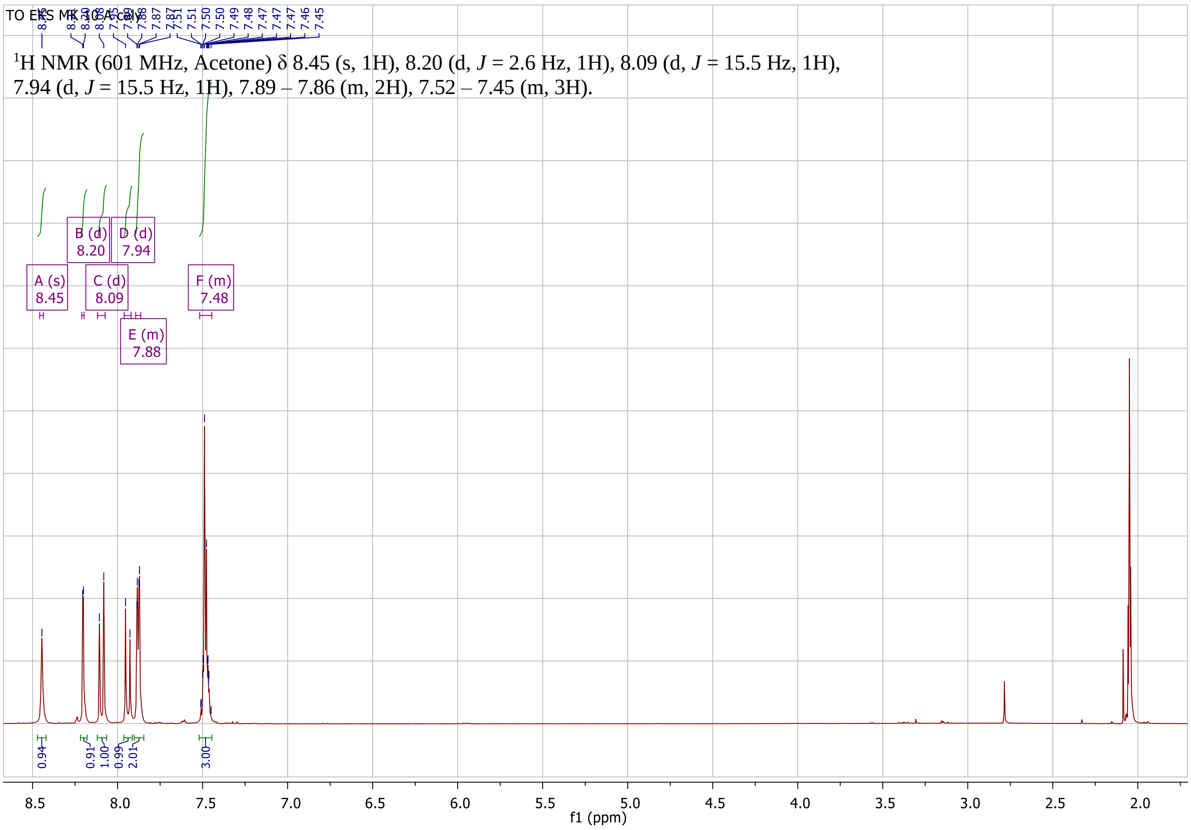

Figure S2. ¹H NMR spectrum of 5′-chloro-2′-hydroxy-3′-nitrochalcone (4).

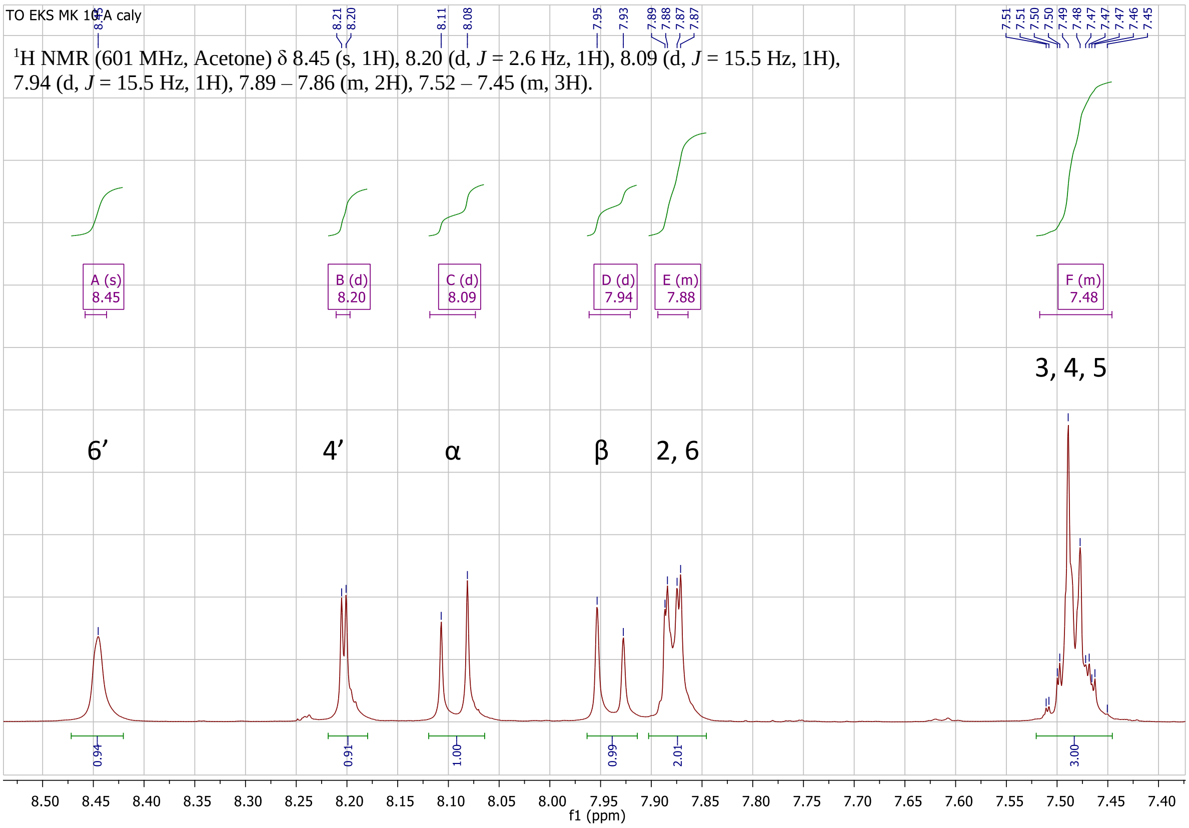

Figure S3. ¹H NMR spectrum expansion of 5′-chloro-2′-hydroxy-3′-nitrochalcone (4).

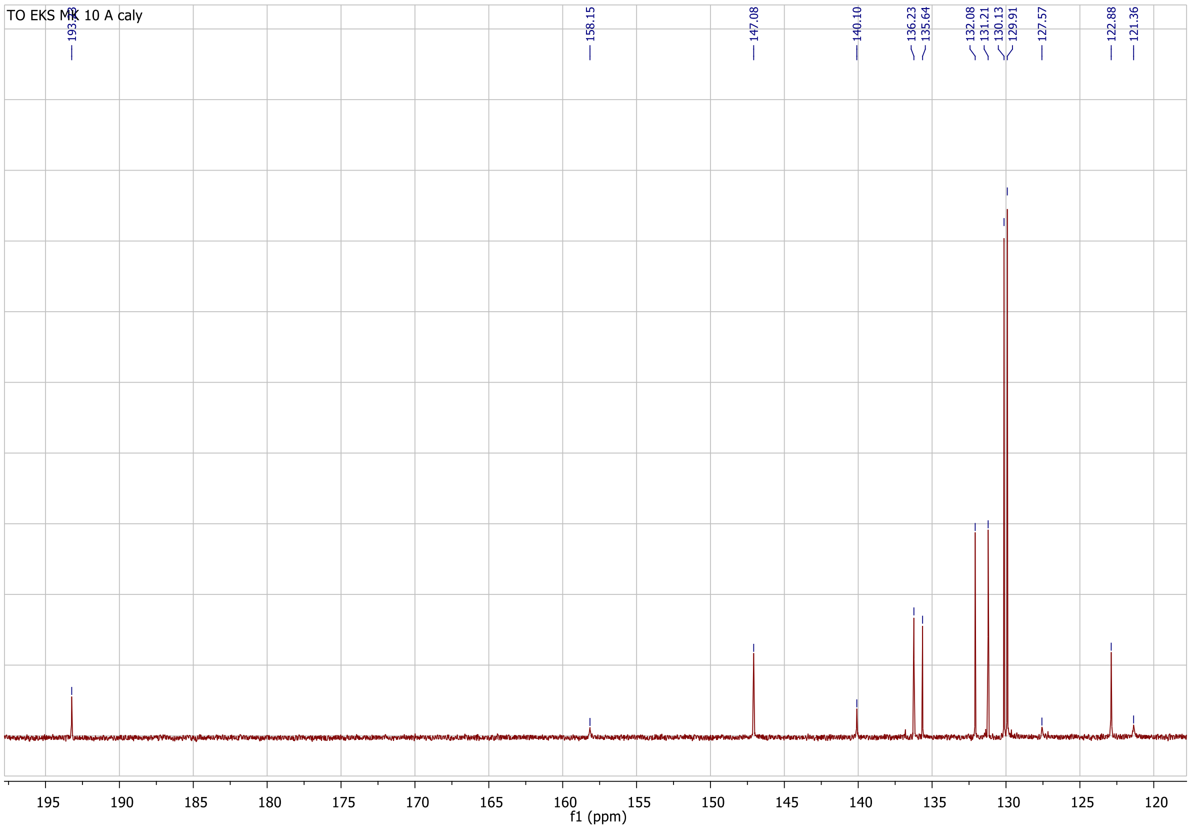

Figure S4. ^{13}C NMR of 5′-chloro-2′-hydroxy-3′-nitrochalcone (4).

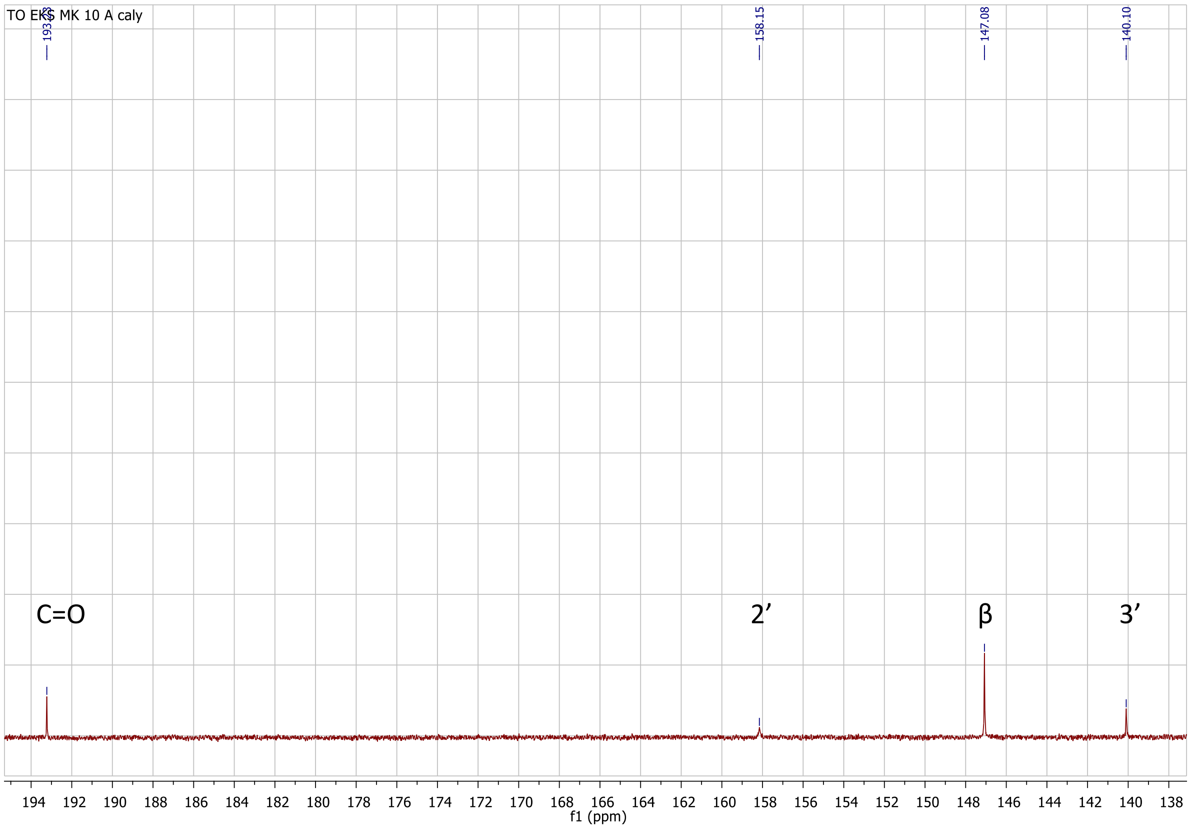

Figure S5. ^{13}C NMR spectrum expansion of 5′-chloro-2′-hydroxy-3′-nitrochalcone (4).

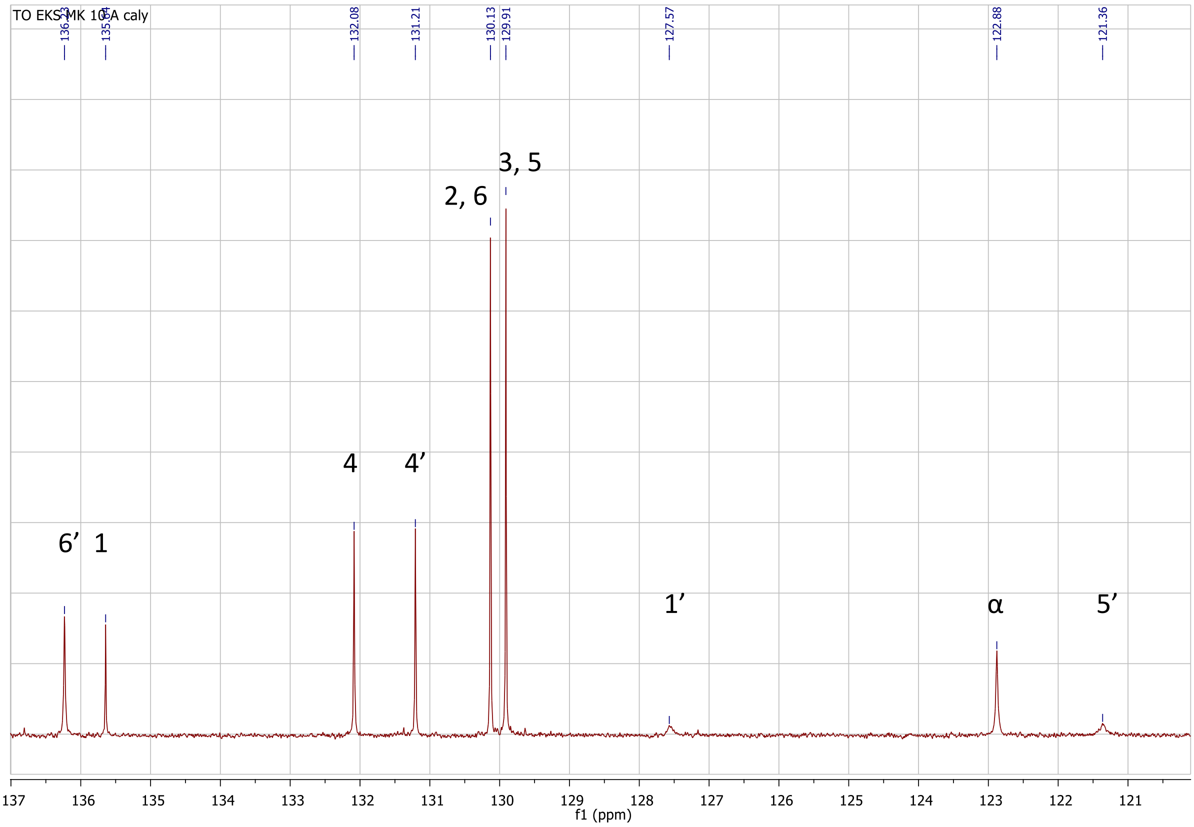

Figure S6. ^{13}C NMR spectrum expansion of 5′-chloro-2′-hydroxy-3′-nitrochalcone (4).

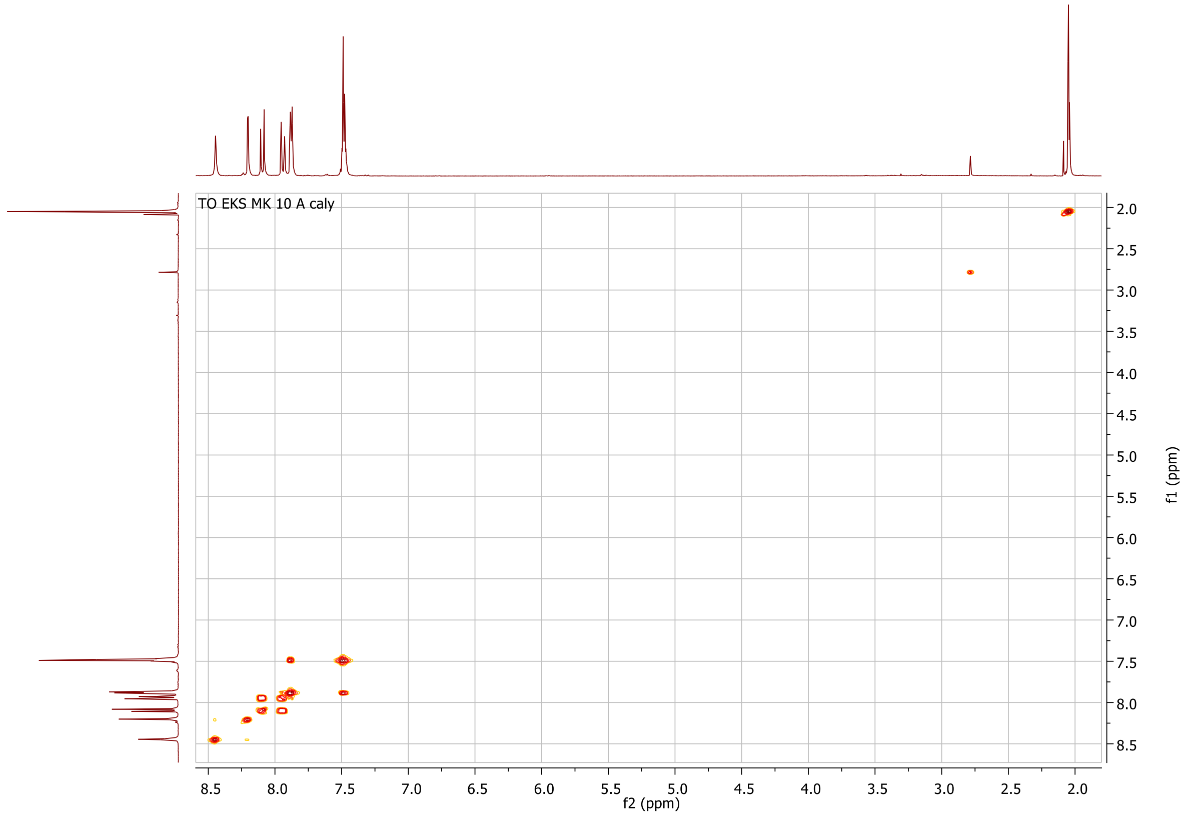

Figure S7. COSY contour map – ^{1}H x ^{1}H of 5′-chloro-2′-hydroxy-3′-nitrochalcone (4).

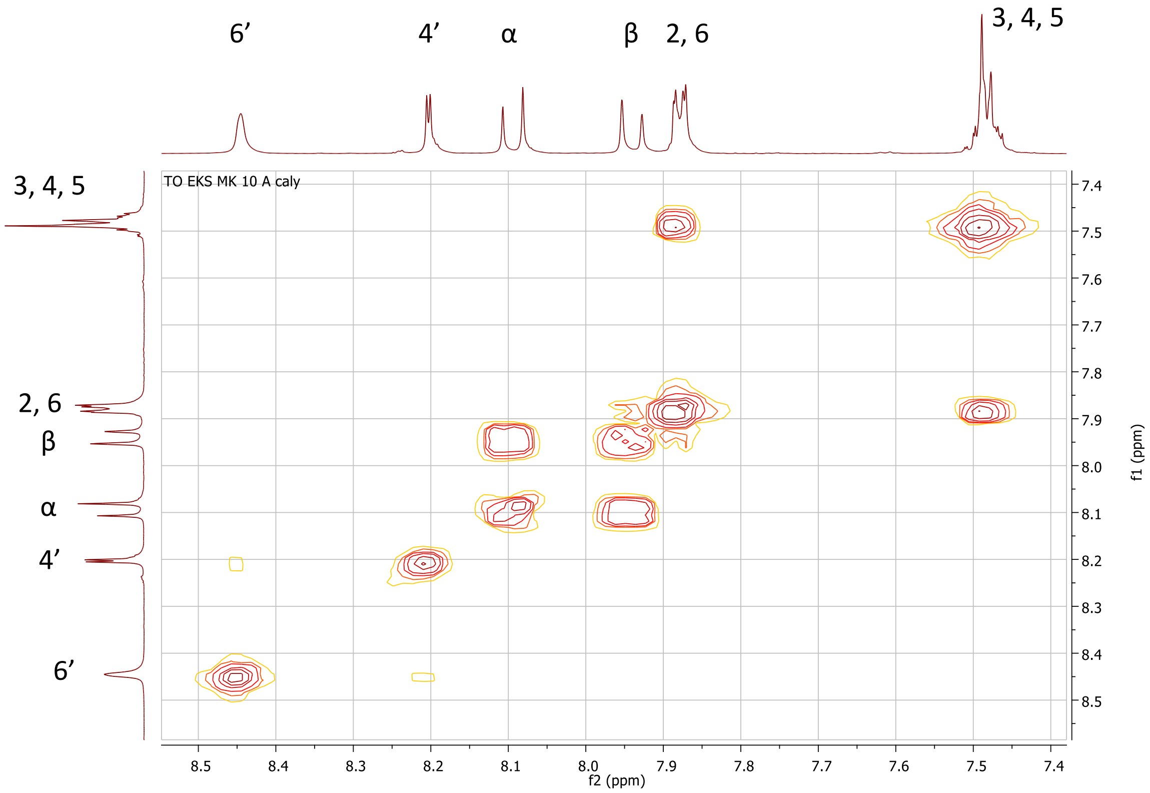

Figure S8. COSY contour map – ^{1}H x ^{1}H expansion of 5′-chloro-2′-hydroxy-3′-nitrochalcone (4).

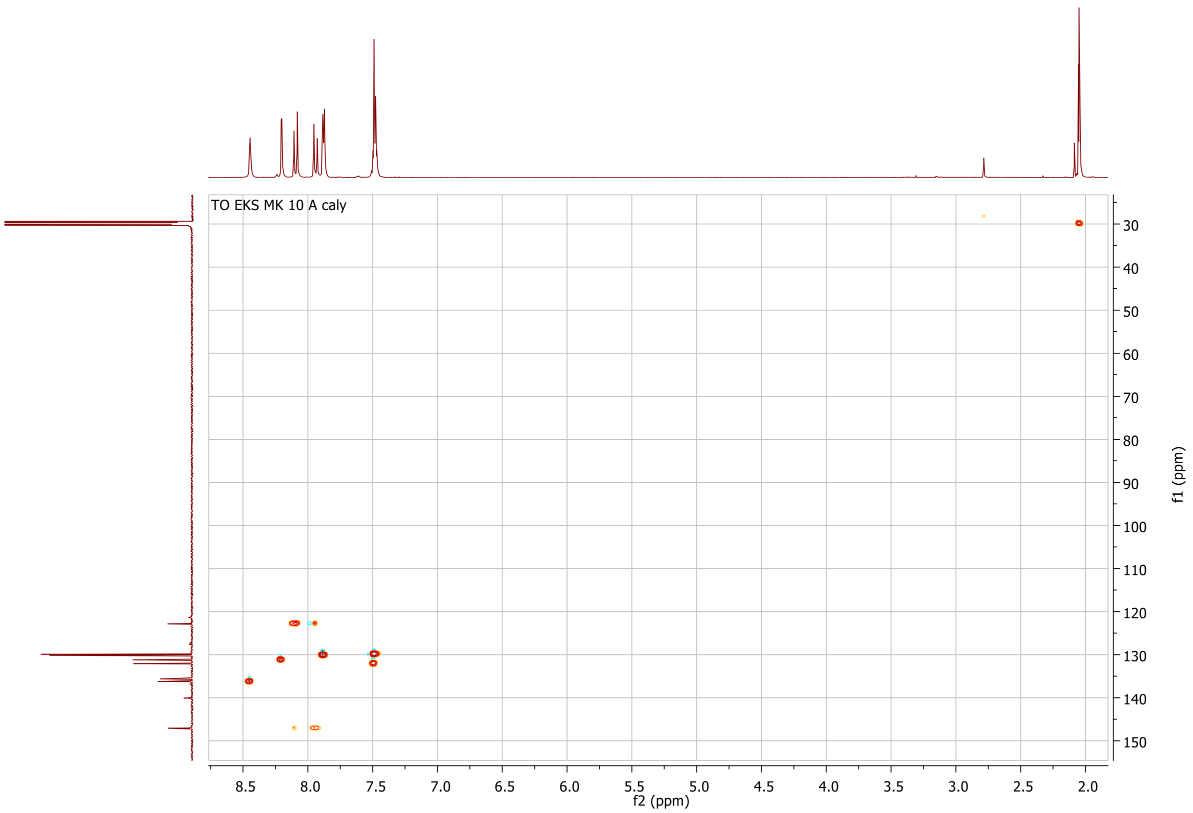

Figure S9. HSQC contour map – ^{1}H x ^{13}C of 5′-chloro-2′-hydroxy-3′-nitrochalcone (4).

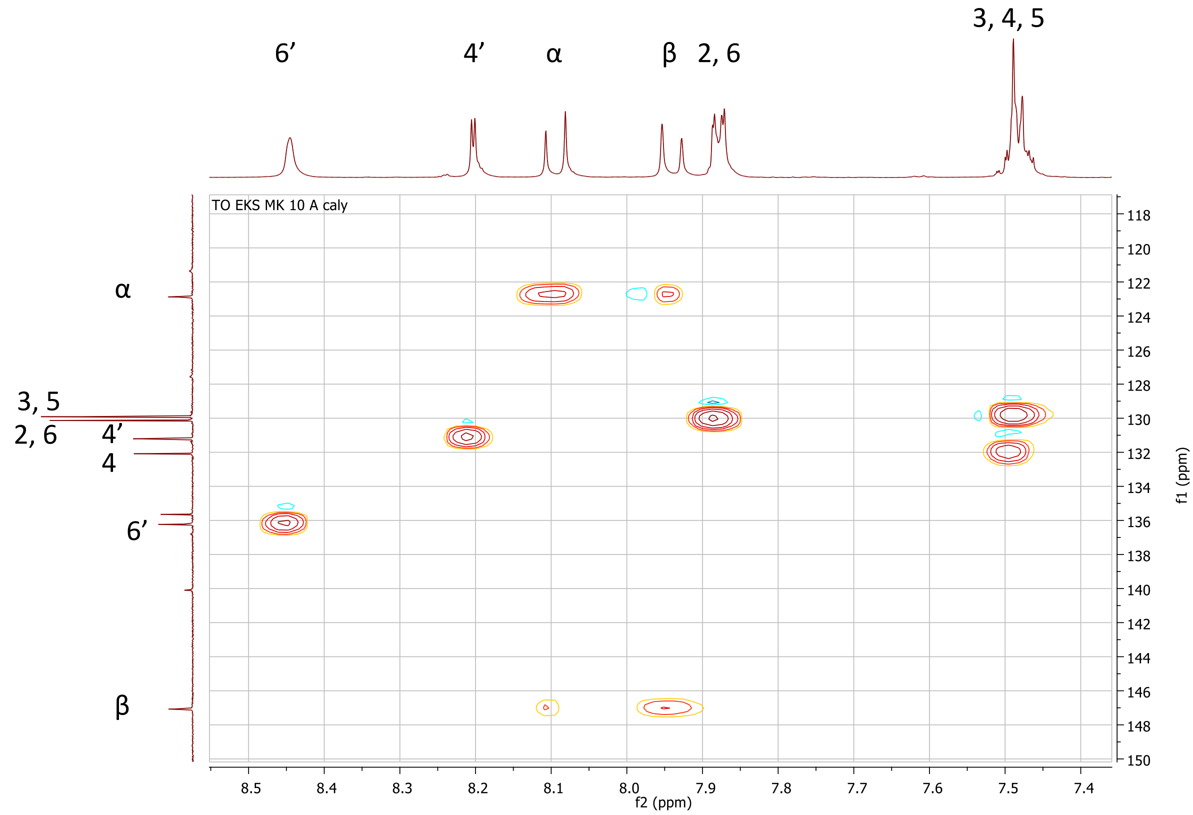

Figure S10. HSQC contour map – ^{1}H x ^{13}C expansion of 5′-chloro-2′-hydroxy-3′-nitrochalcone (4).

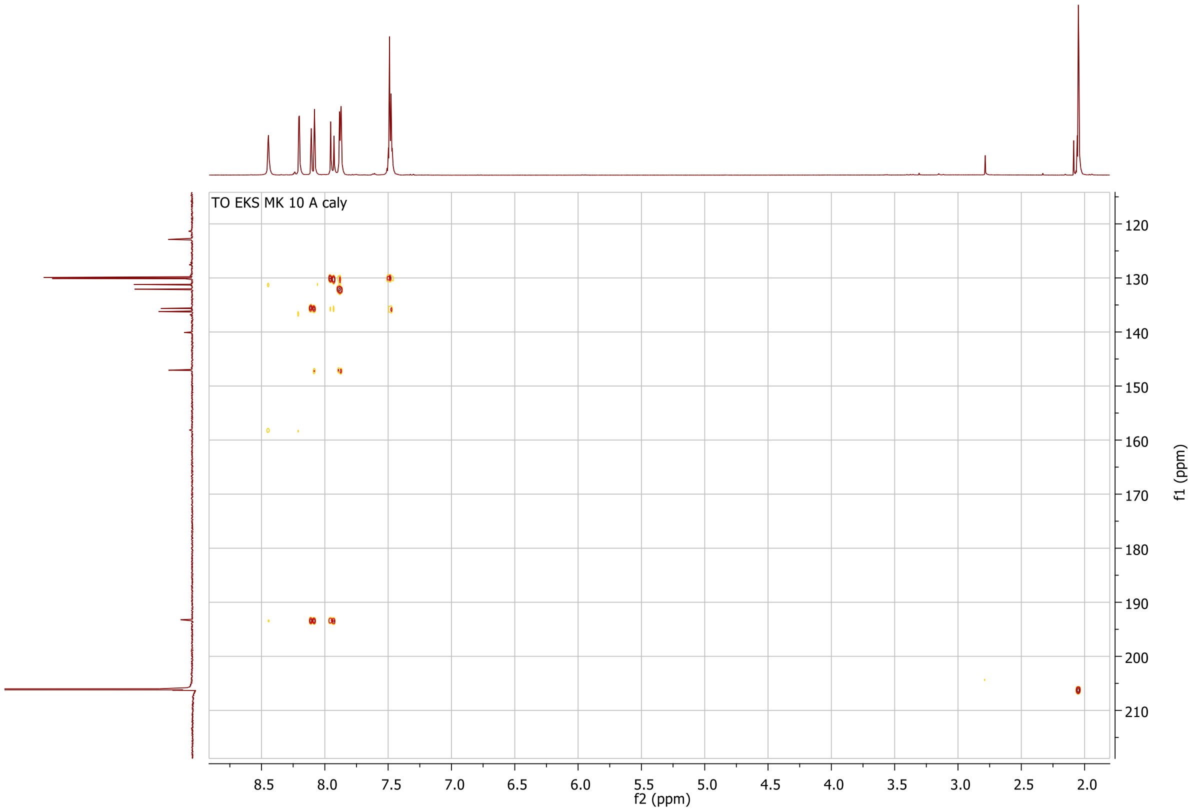

Figure S11. HMBC contour map – ^{1}H x ^{13}C of 5′-chloro-2′-hydroxy-3′-nitrochalcone (4).

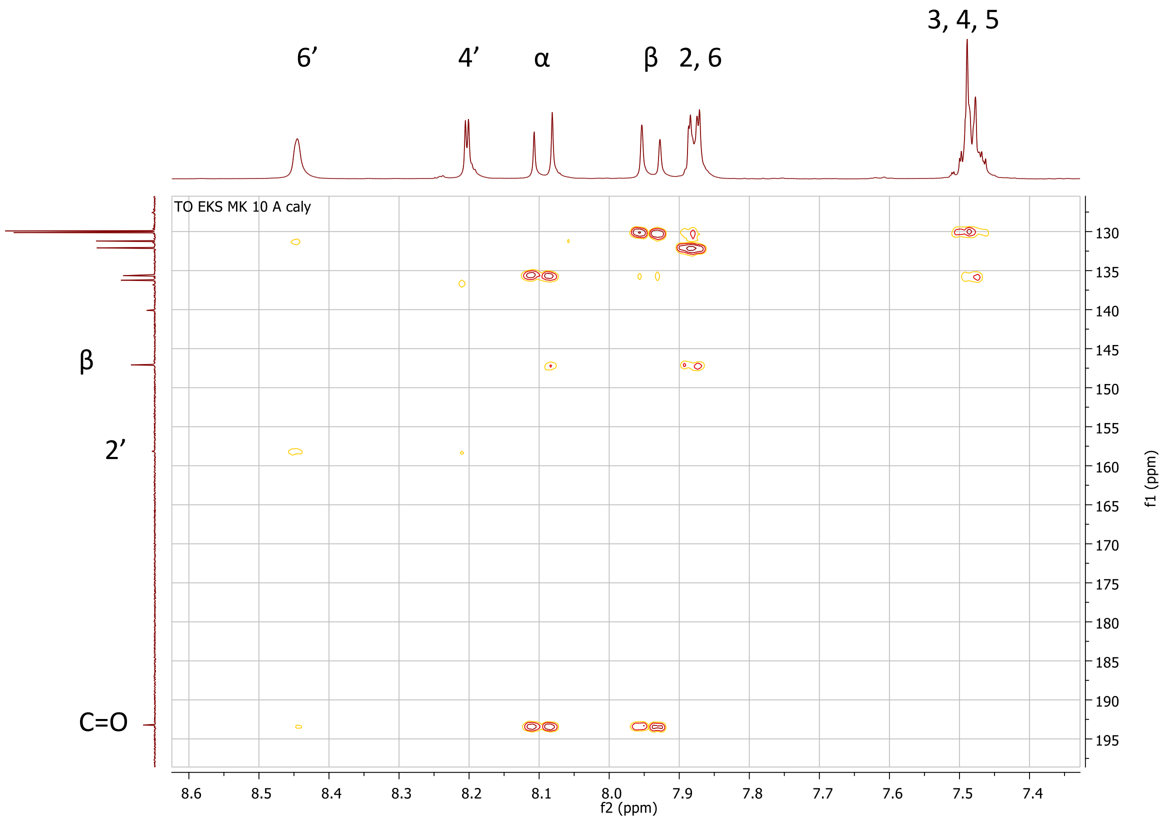

Figure S12. HMBC contour map – ^{1}H x ^{13}C expansion of 5′-chloro-2′-hydroxy-3′-nitrochalcone (4).

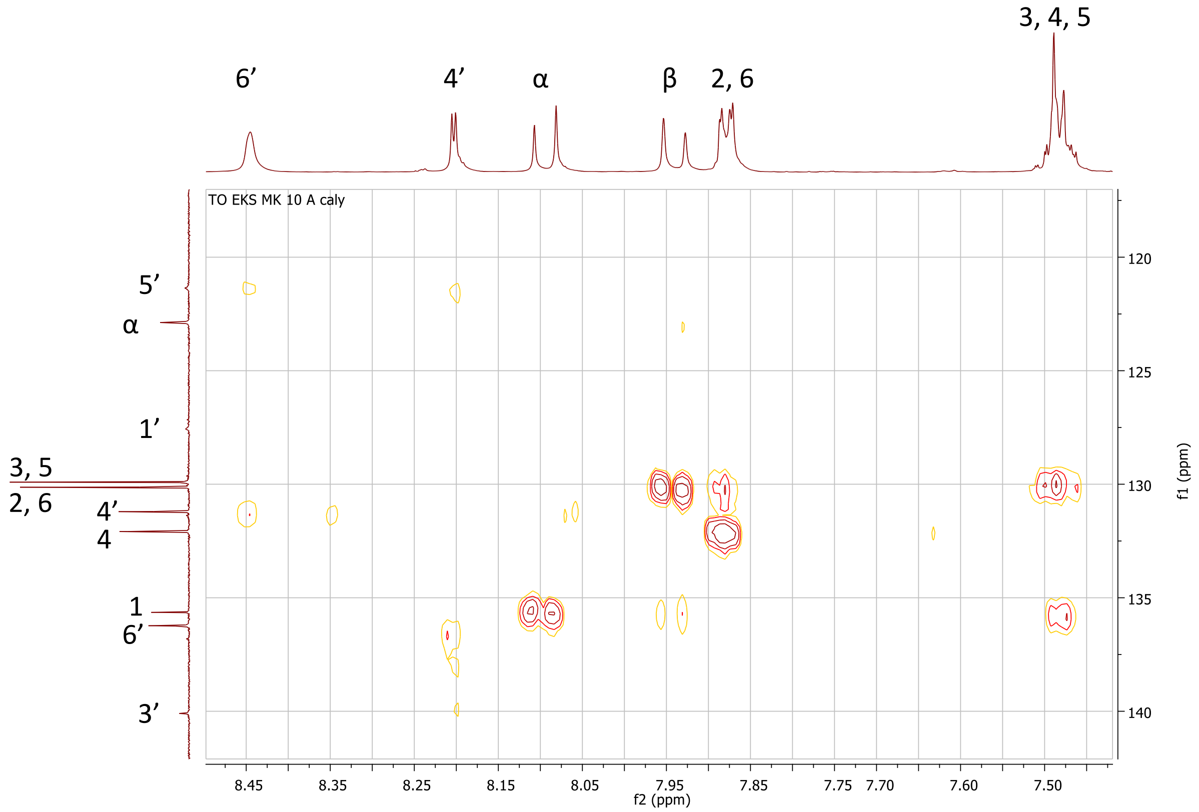

Figure S13. HMBC contour map – ^{1}H x ^{13}C expansion of 5′-chloro-2′-hydroxy-3′-nitrochalcone (4).

Compound name: 6-chloro-8-nitroflavanone
Molecular Formula: $C_{15}H_{10}ClNO_4$
Formula Weight: 303.697
Ionization mode: negative
Precursor $[M + H]^+$: 302.029
Monoisotopic Mass: 303.029
Collision energy (CE): 25.0

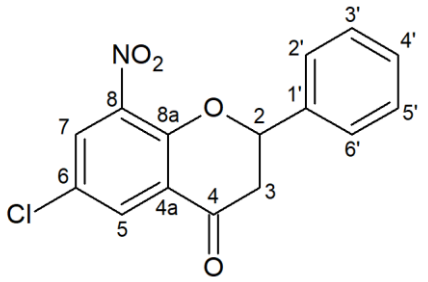

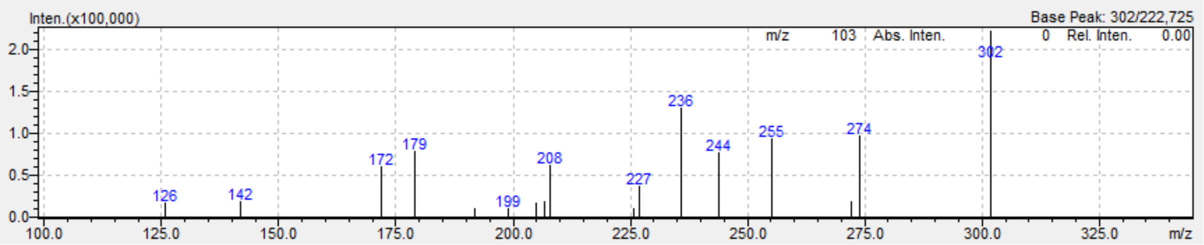

Figure S14. LC-MS spectrum of 6-chloro-8-nitroflavanone.

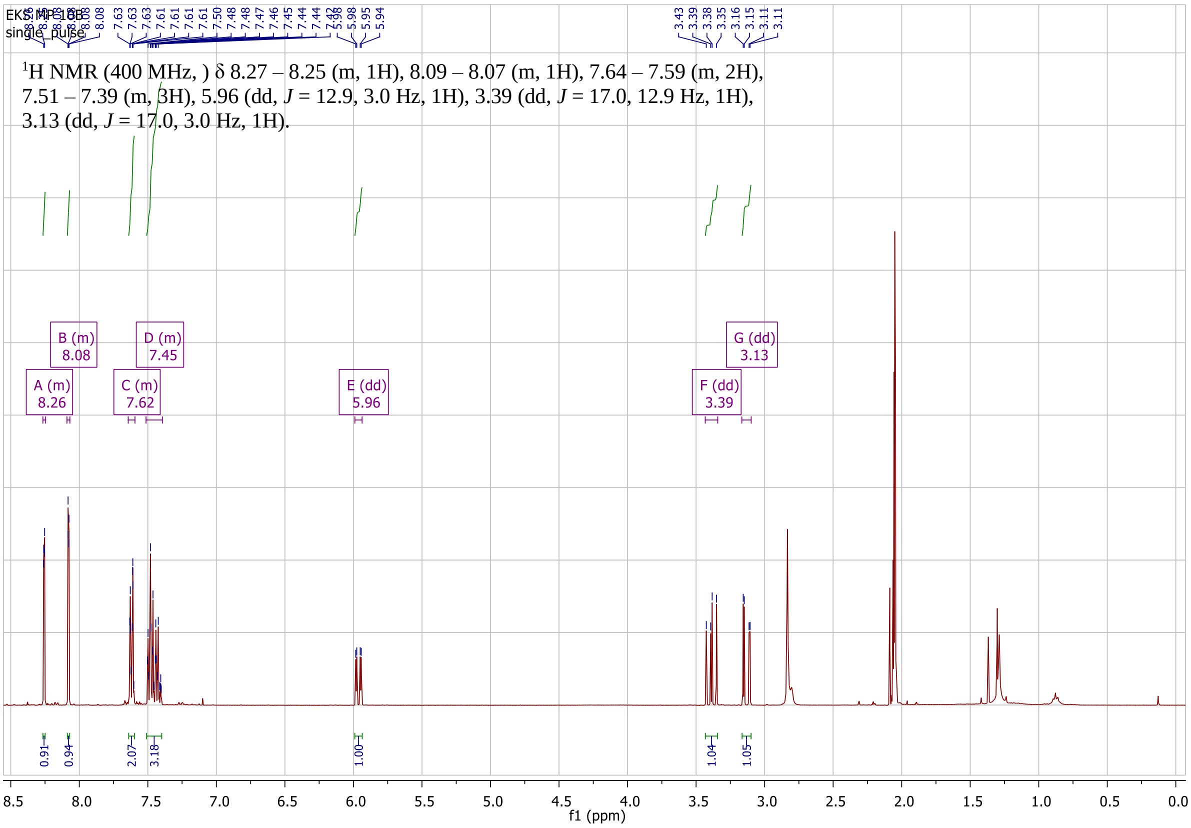

Figure S15. ¹H NMR spectrum of 6-chloro-8-nitroflavanone.

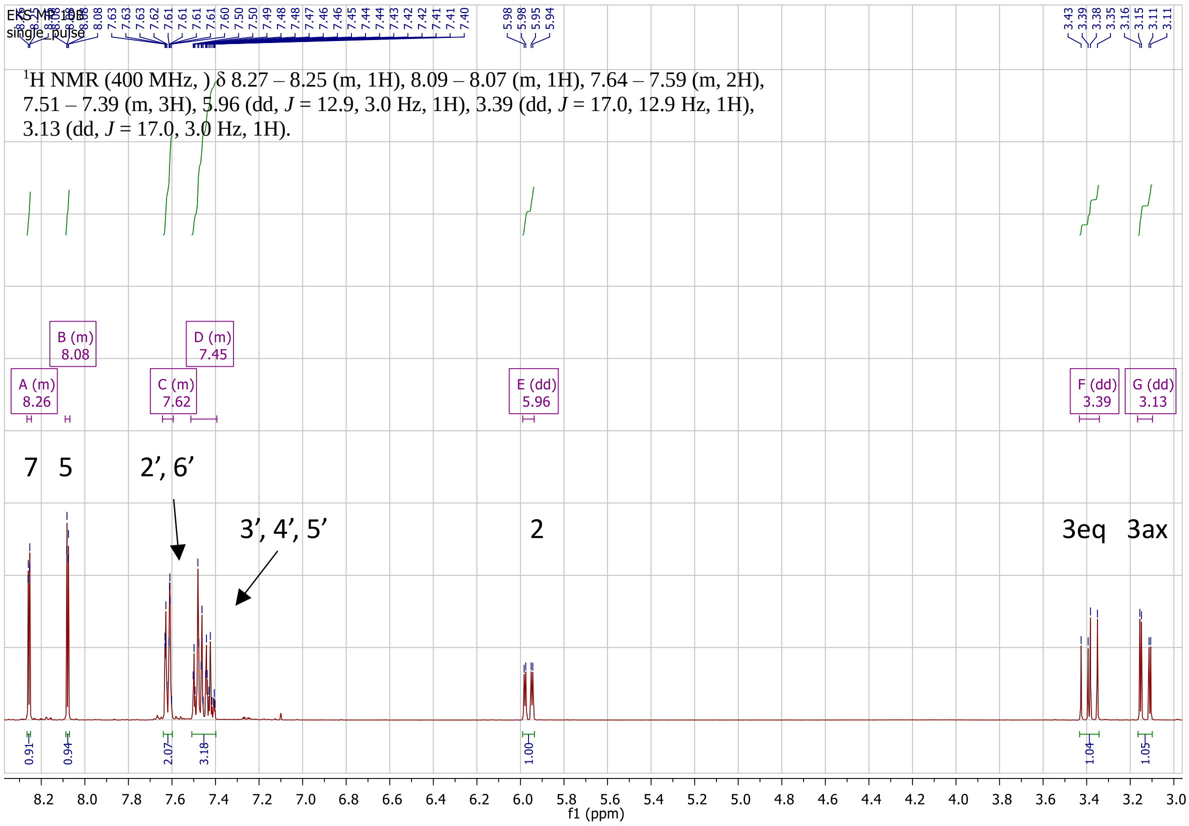

Figure S16. ^{1}H NMR spectrum expansion of 6-chloro-8-nitroflavanone.

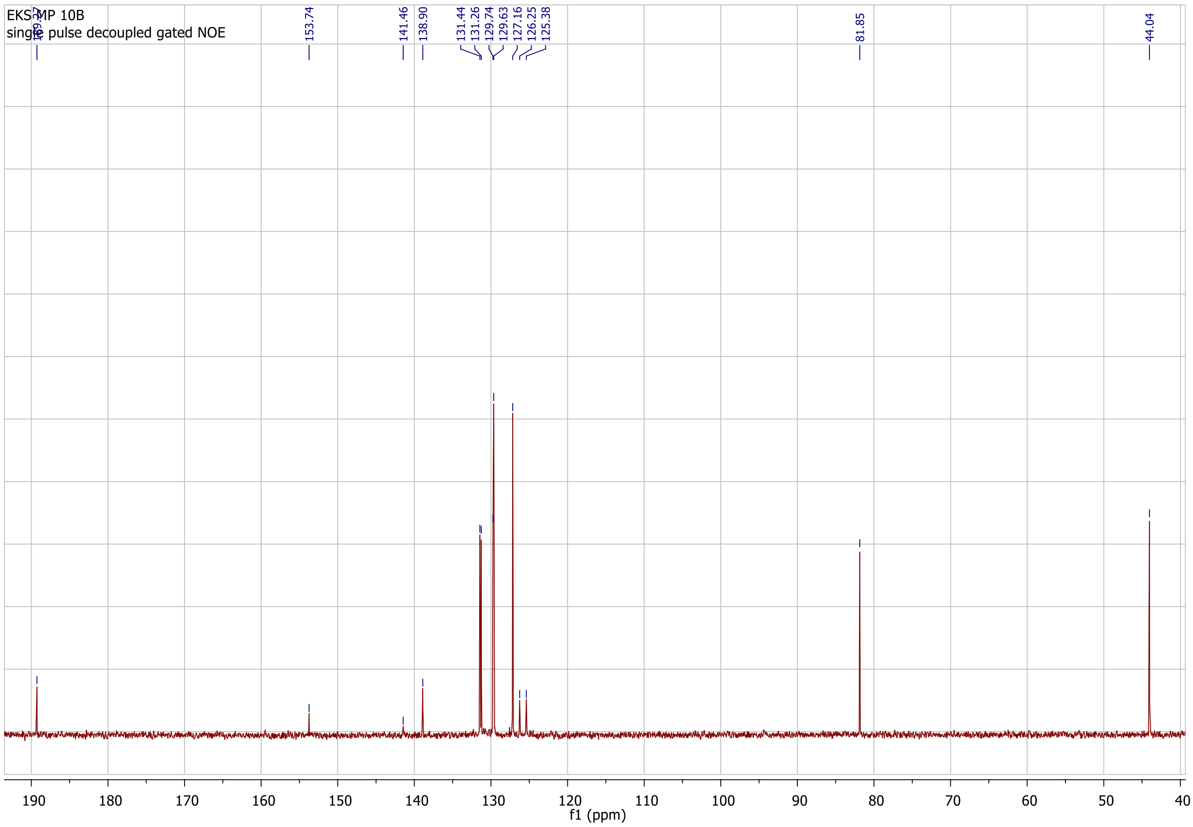

Figure S17. ^{13}C NMR spectrum of 6-chloro-8-nitroflavanone.

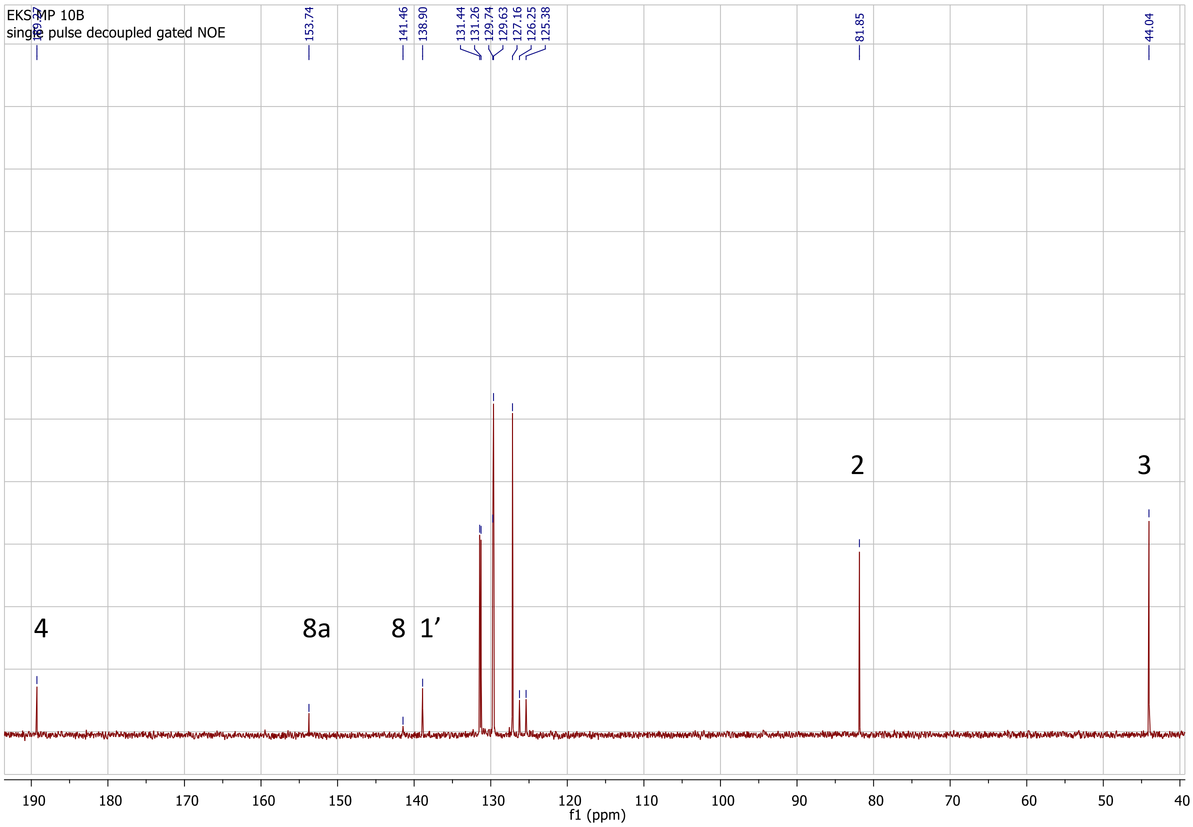

Figure S18. [13]*C NMR spectrum expansion of 6-chloro-8-nitroflavanone.*

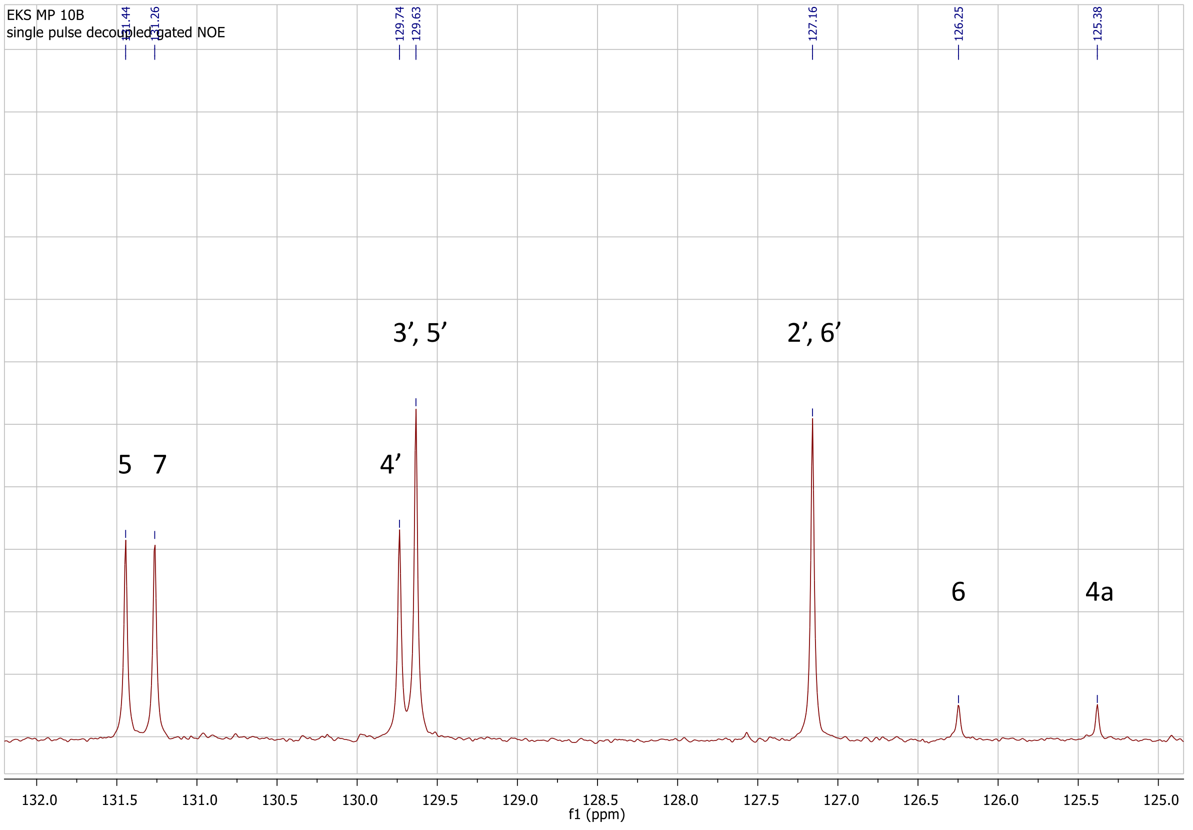

Figure S19. [13]*C NMR spectrum expansion of 6-chloro-8-nitroflavanone*

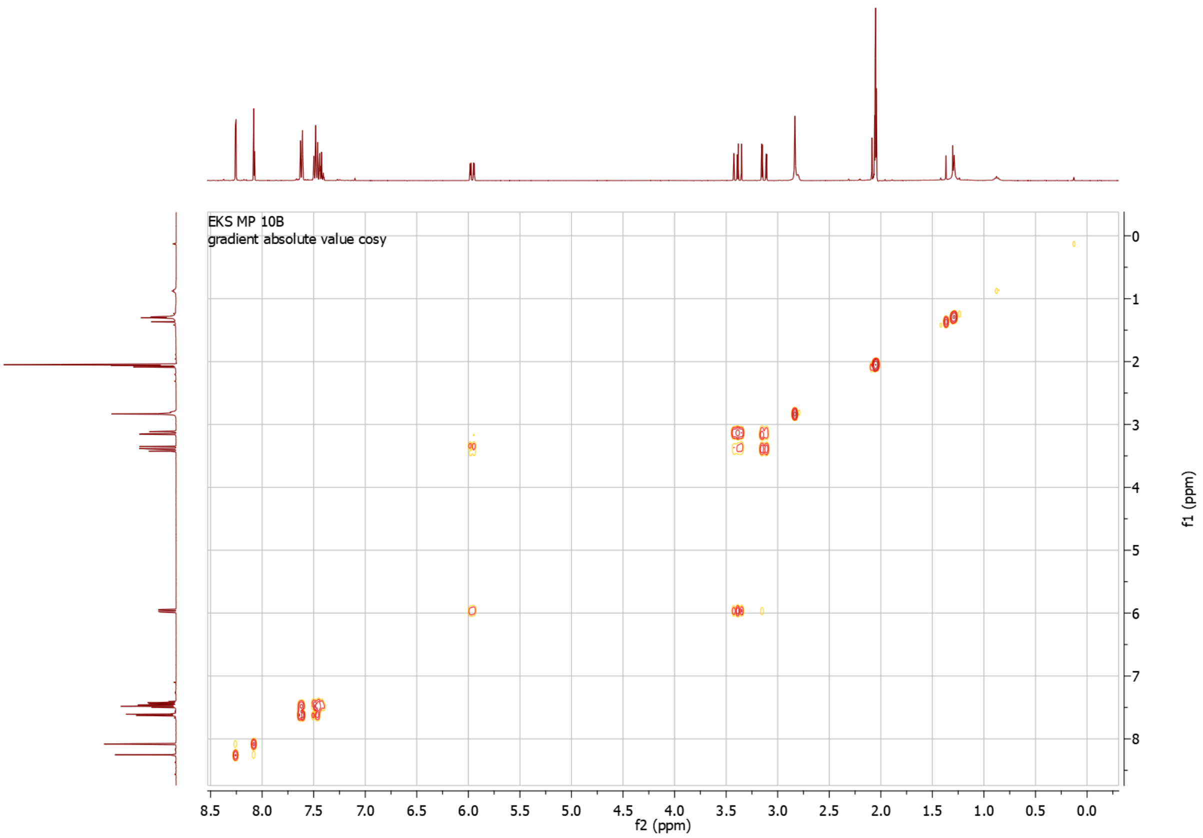

Figure S20. COSY contour map – ^{1}H x ^{1}H expansion of 6-chloro-8-nitroflavanone.

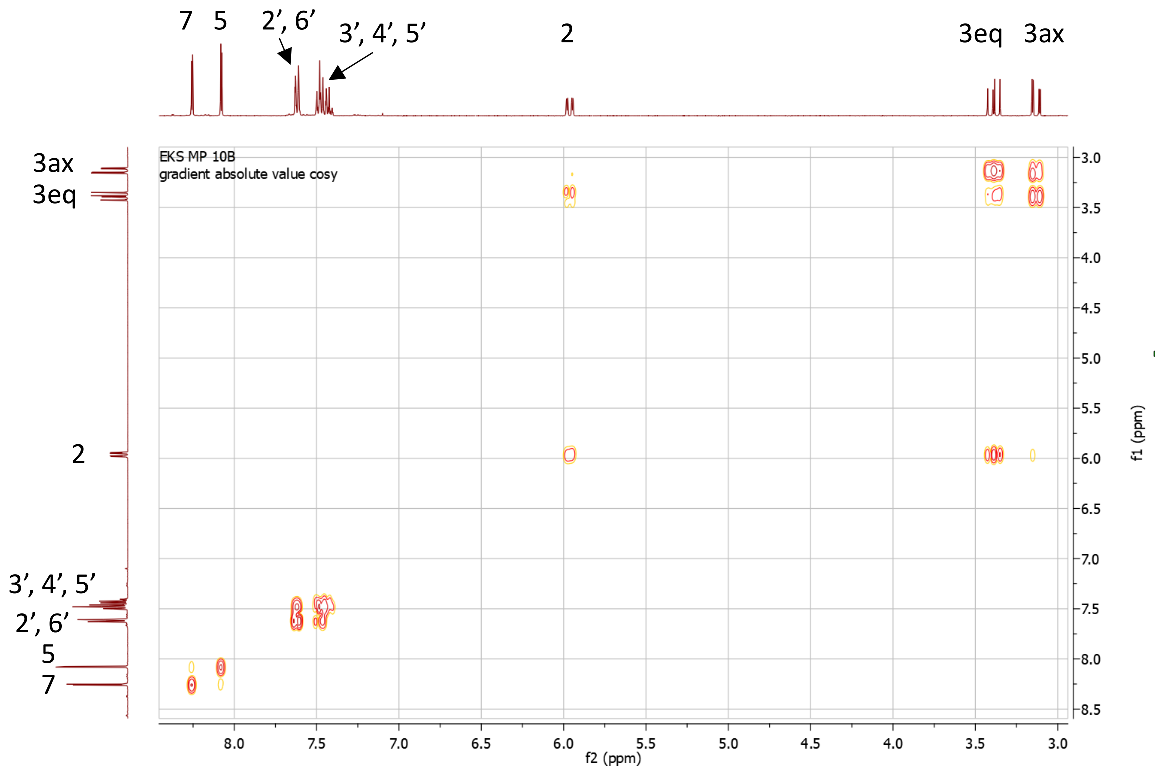

Figure S21. COSY contour map – ^{1}H x ^{1}H expansion of 6-chloro-8-nitroflavanone.

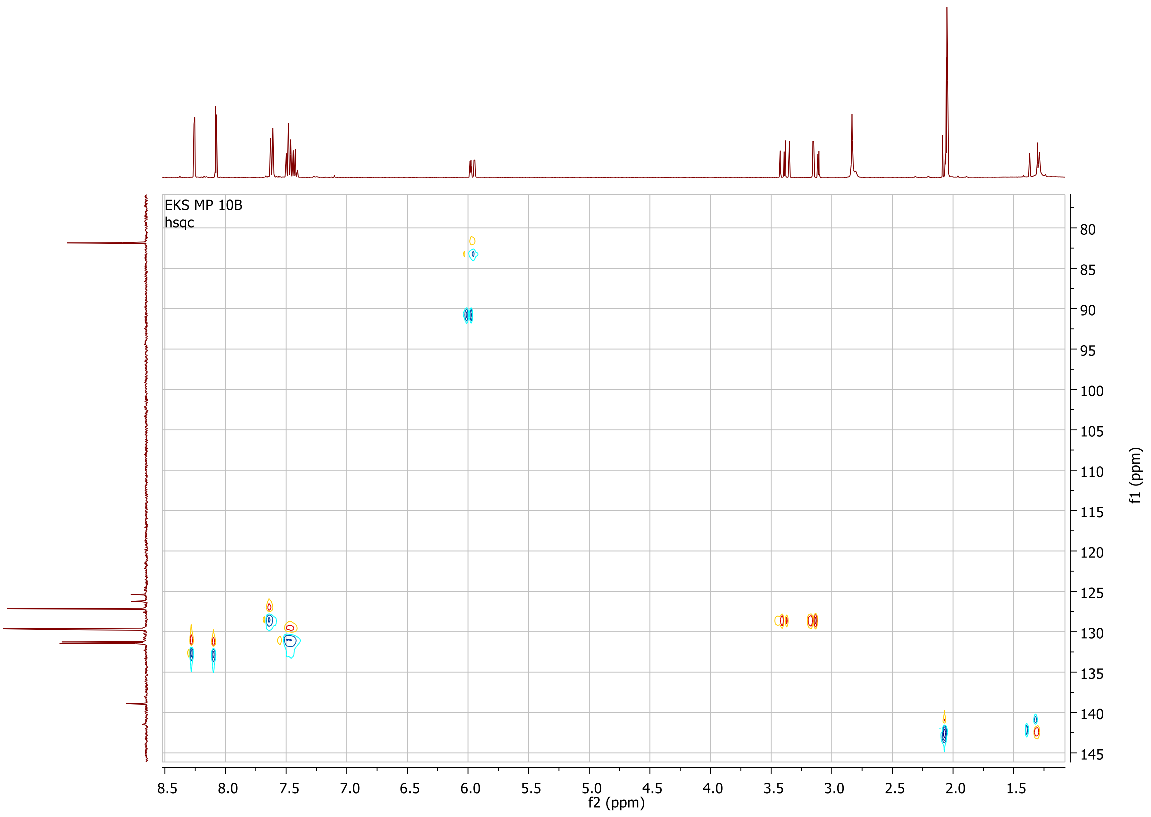

Figure S22. HSQC contour map – ^{1}H x ^{13}C of 6-chloro-8-nitroflavanone.

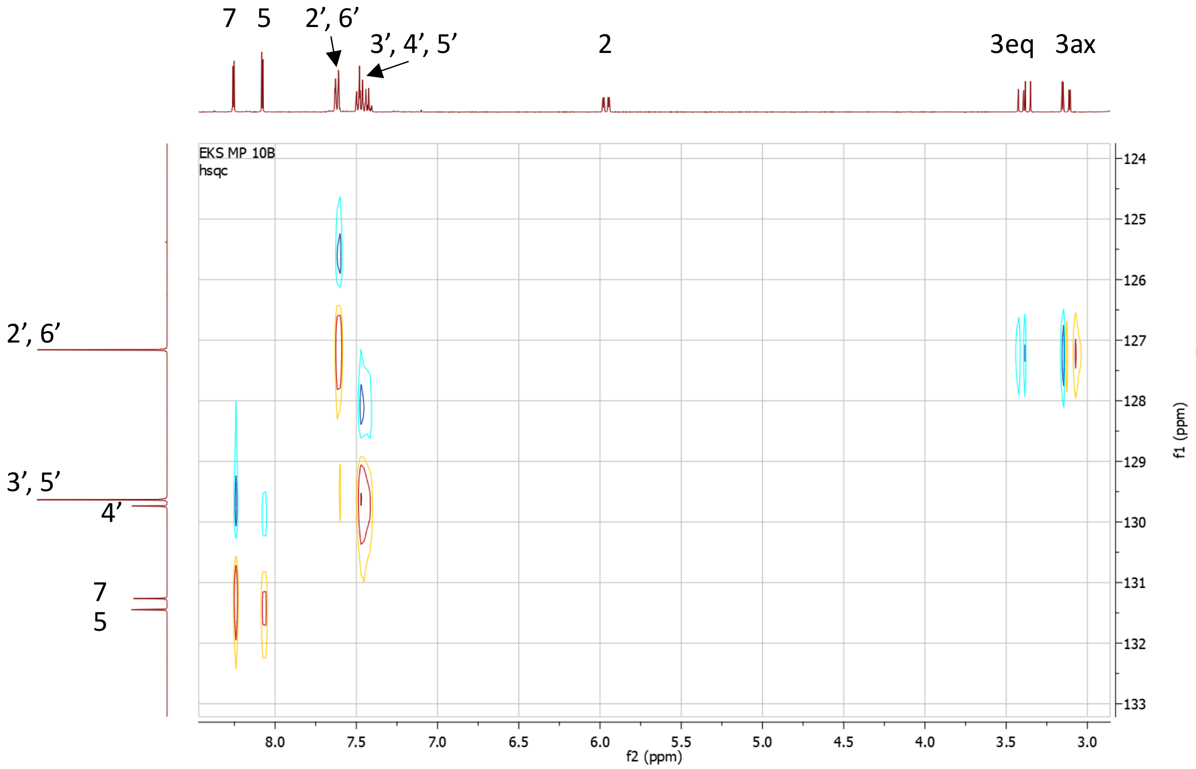

Figure S23. HSQC contour map – ^{1}H x ^{13}C expansion of 6-chloro-8-nitroflavanone.

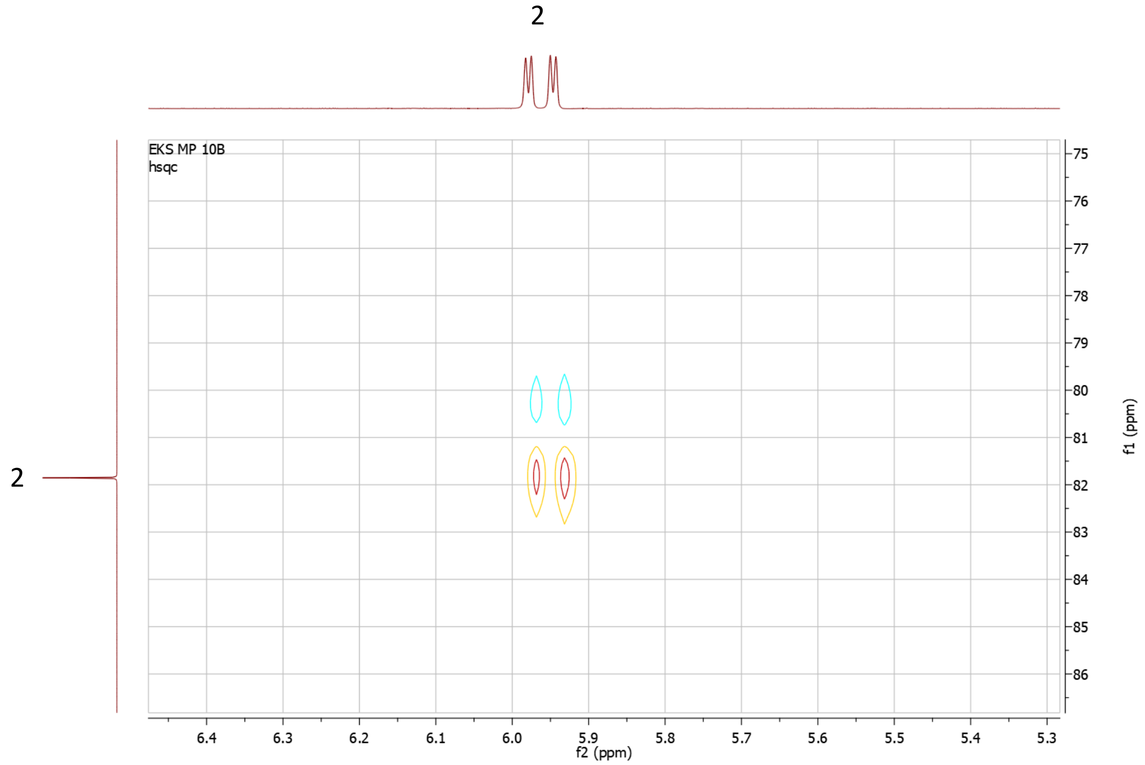

Figure S24. HSQC contour map – ¹H x ¹³C expansion of 6-chloro-8-nitroflavanone.

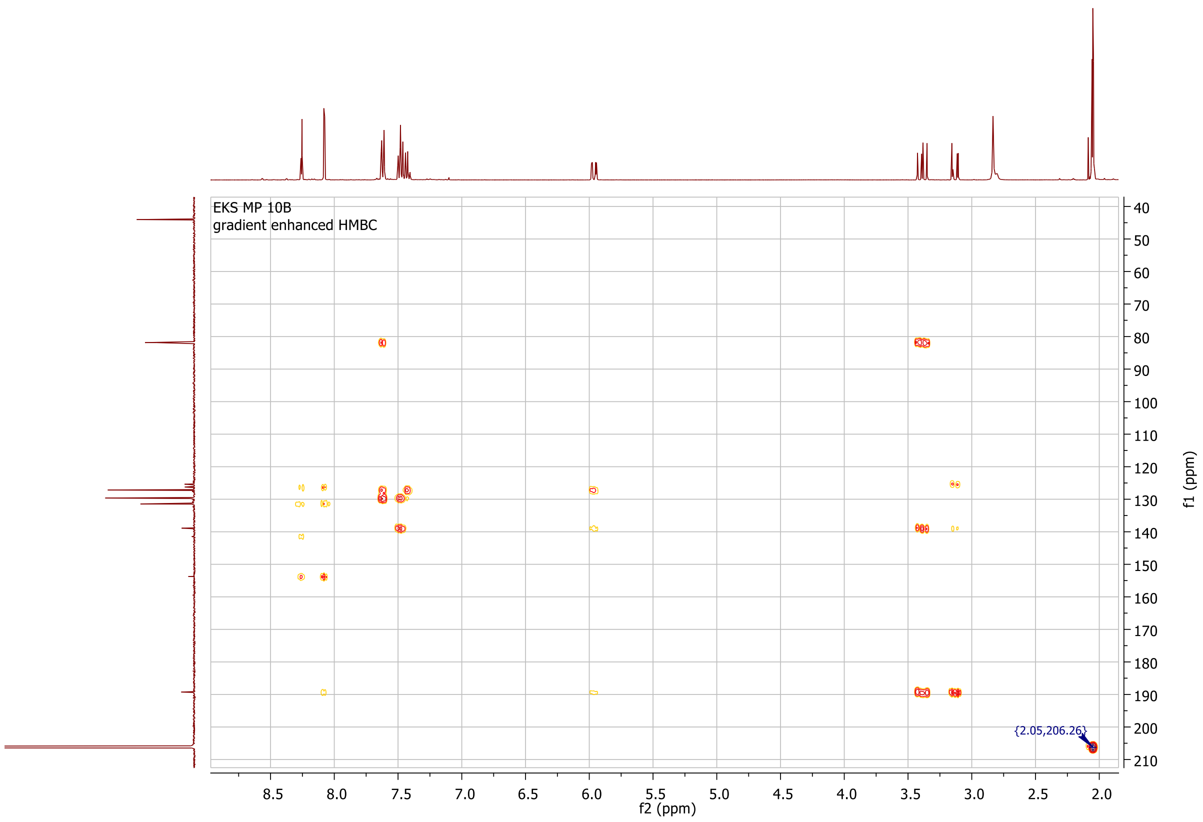

Figure S25. HMBC contour map – ¹H x ¹³C of 6-chloro-8-nitroflavanone.

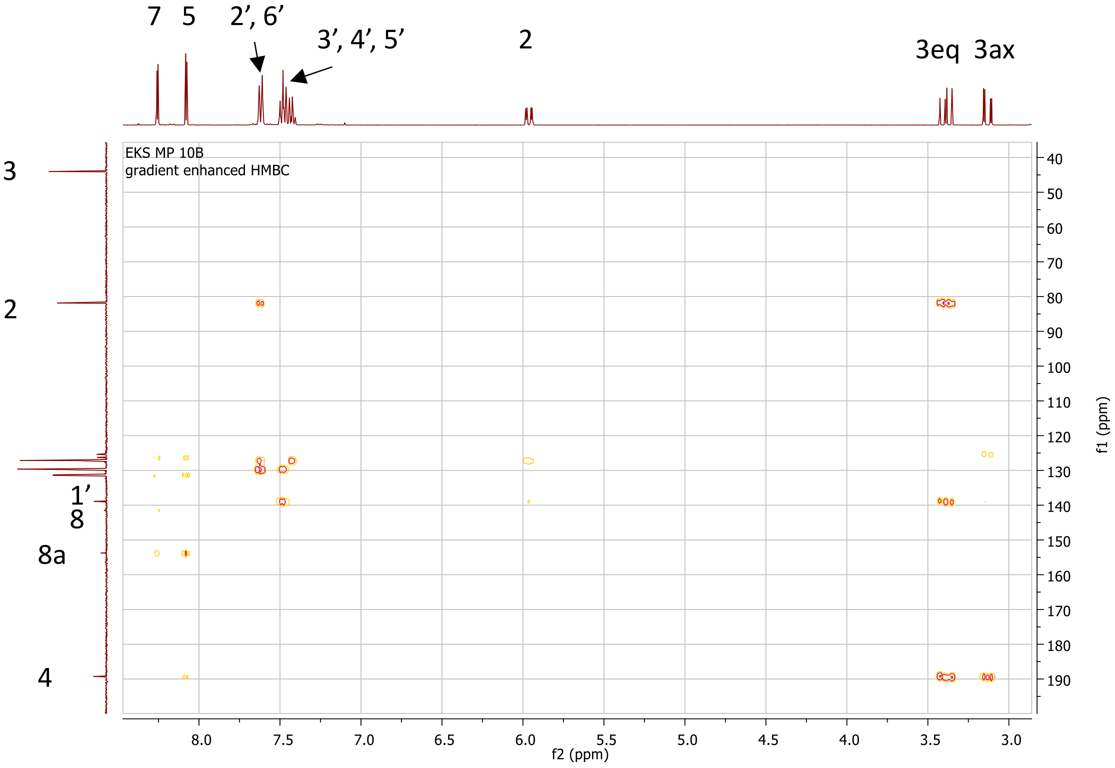

Figure S26. HMBC contour map – ^{1}H x ^{13}C expansion of 6-chloro-8-nitroflavanone.

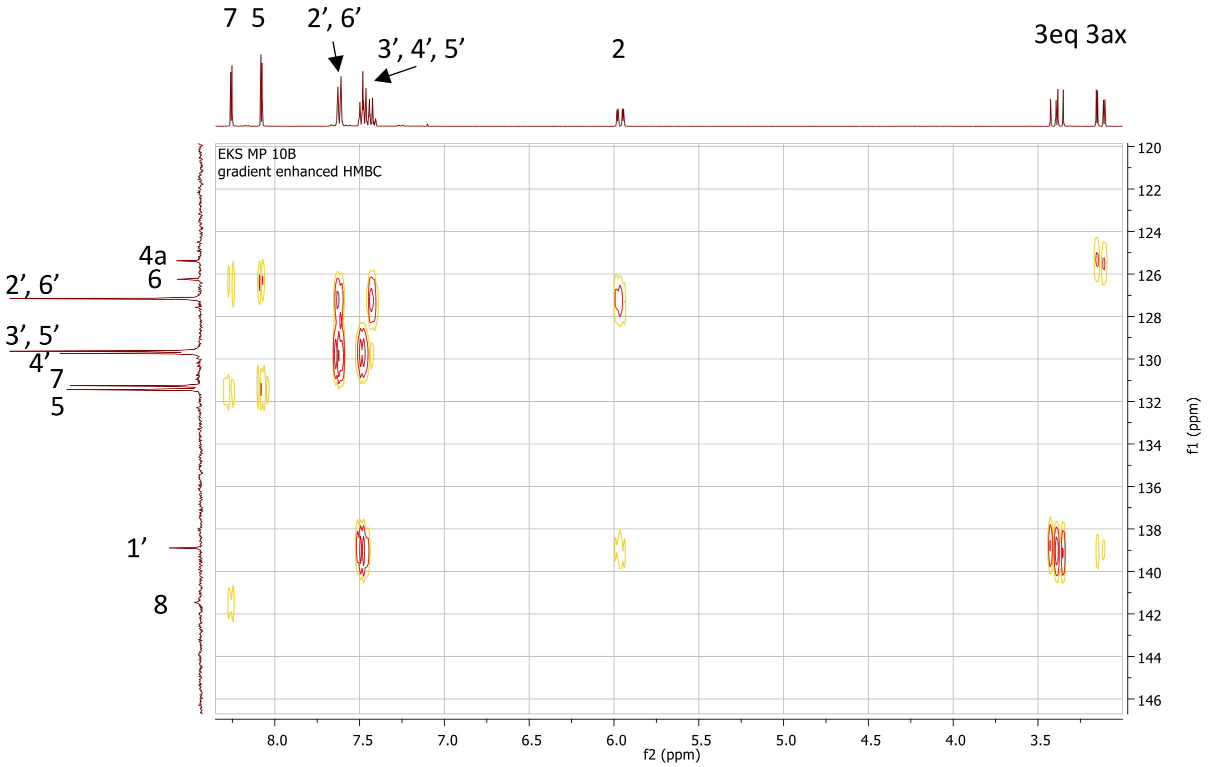

Figure S27. HMBC contour map – ^{1}H x ^{13}C expansion of 6-chloro-8-nitroflavanone.

Compound name: 6-chloro-8-nitroflavone
Molecular Formula: $C_{15}H_8ClNO_4$
Formula Weight: 301.681
Ionization mode: positive
Precursor $[M + H]^+$: 302.014
Monoisotopic Mass: 301.014
Collision energy (CE): -25.0

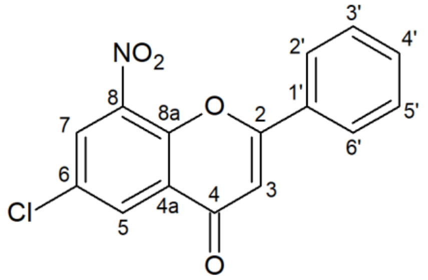

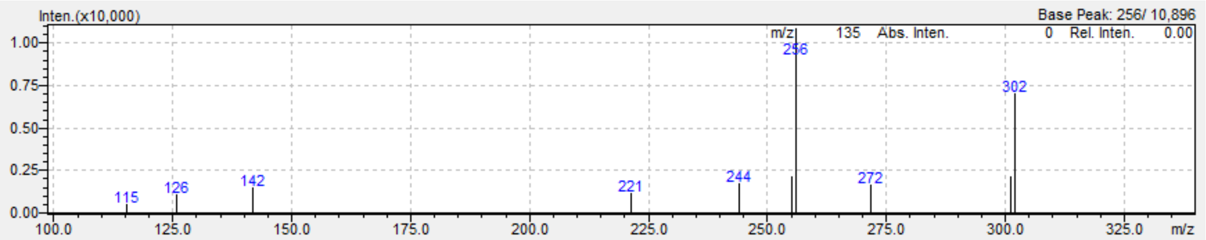

Figure S28. LC-MS spectrum of 6-chloro-8-nitroflavone.

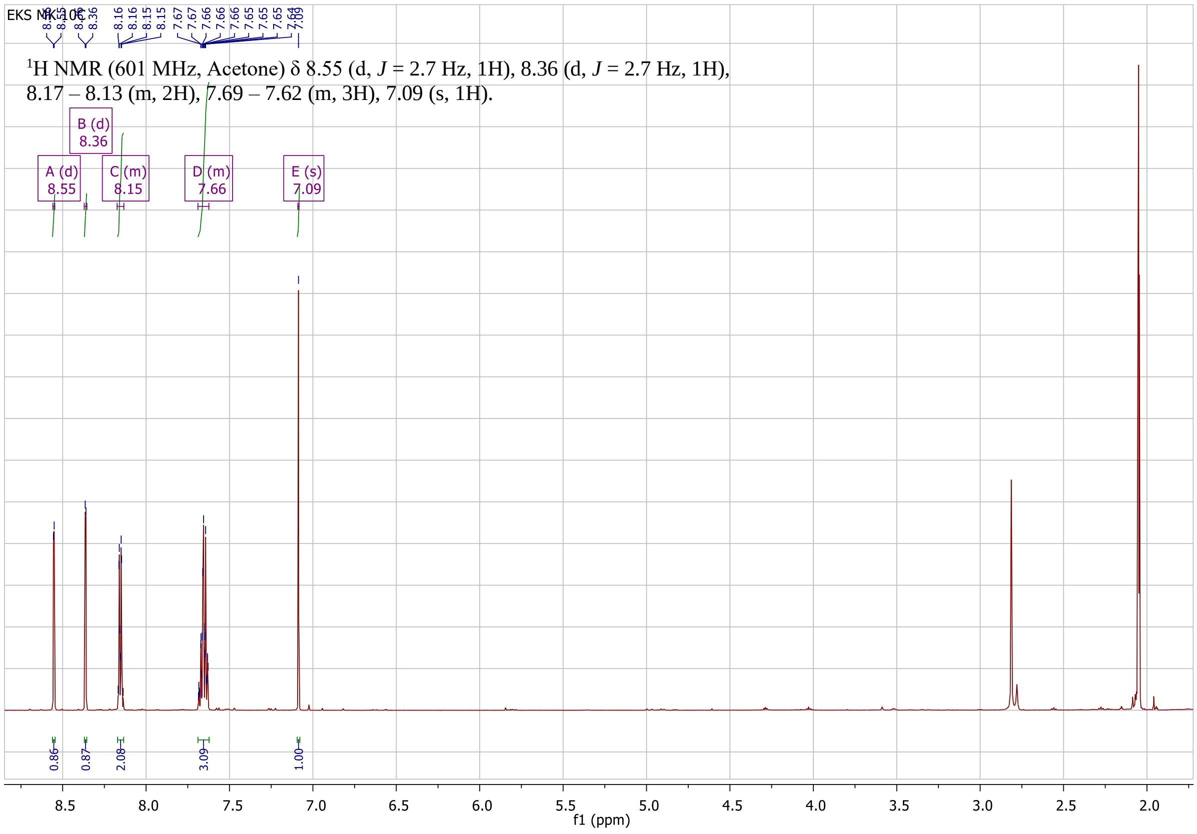

Figure S29. ^{1}H NMR spectrum of 6-chloro-8-nitroflavone.

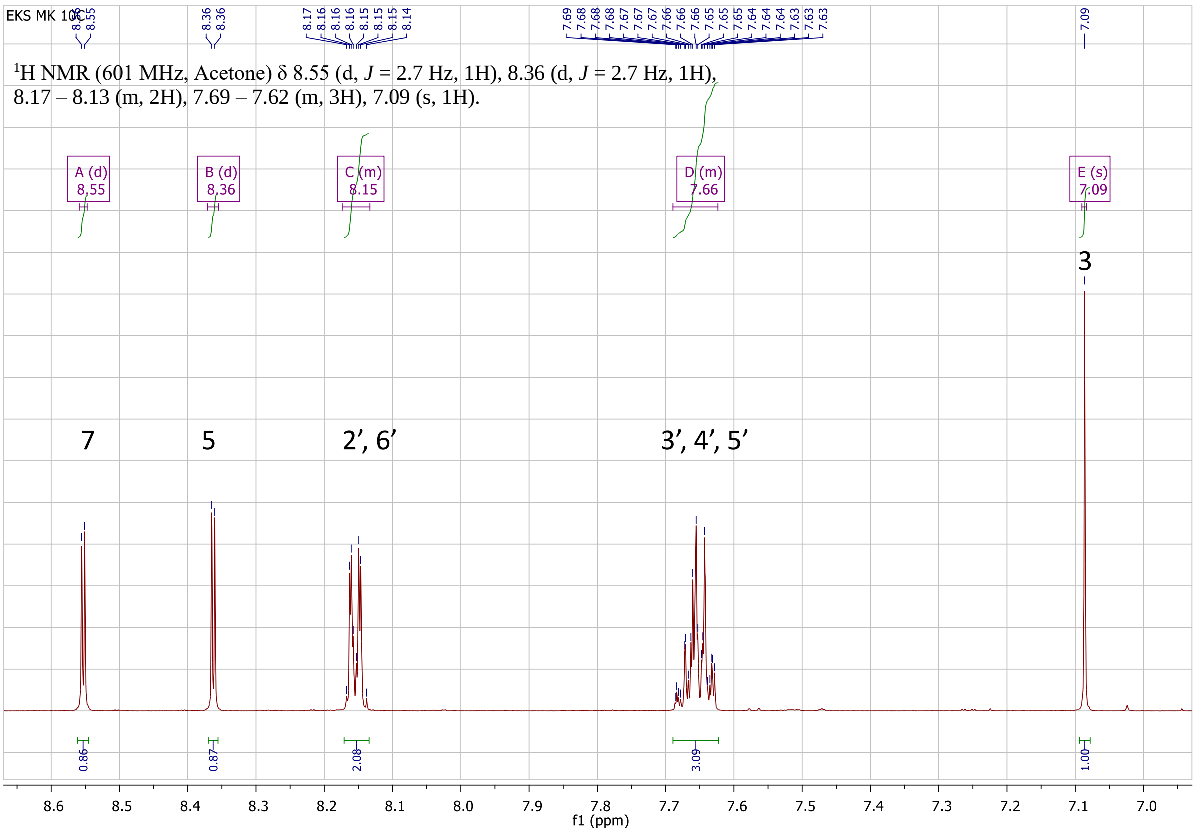

Figure S30. ¹H NMR spectrum expansion of 6-chloro-8-nitroflavone.

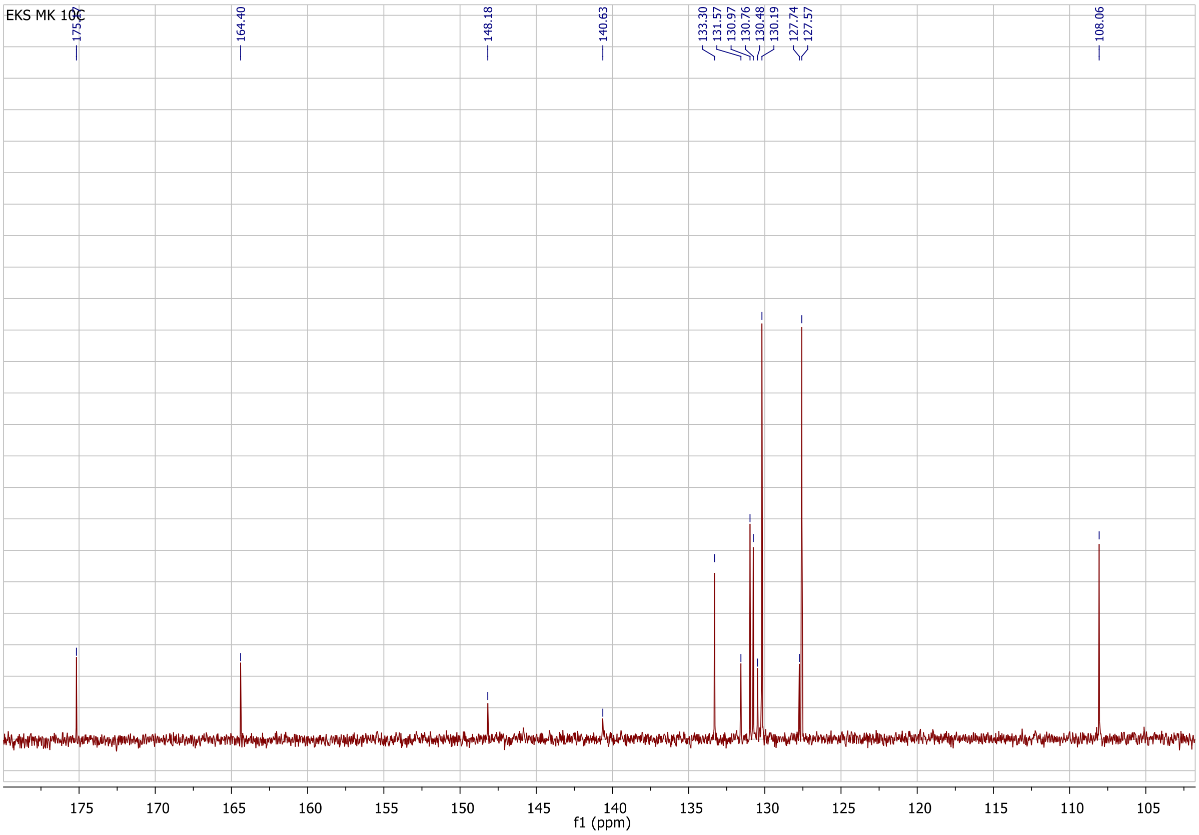

Figure S31. ¹³C NMR spectrum of 6-chloro-8-nitroflavone.

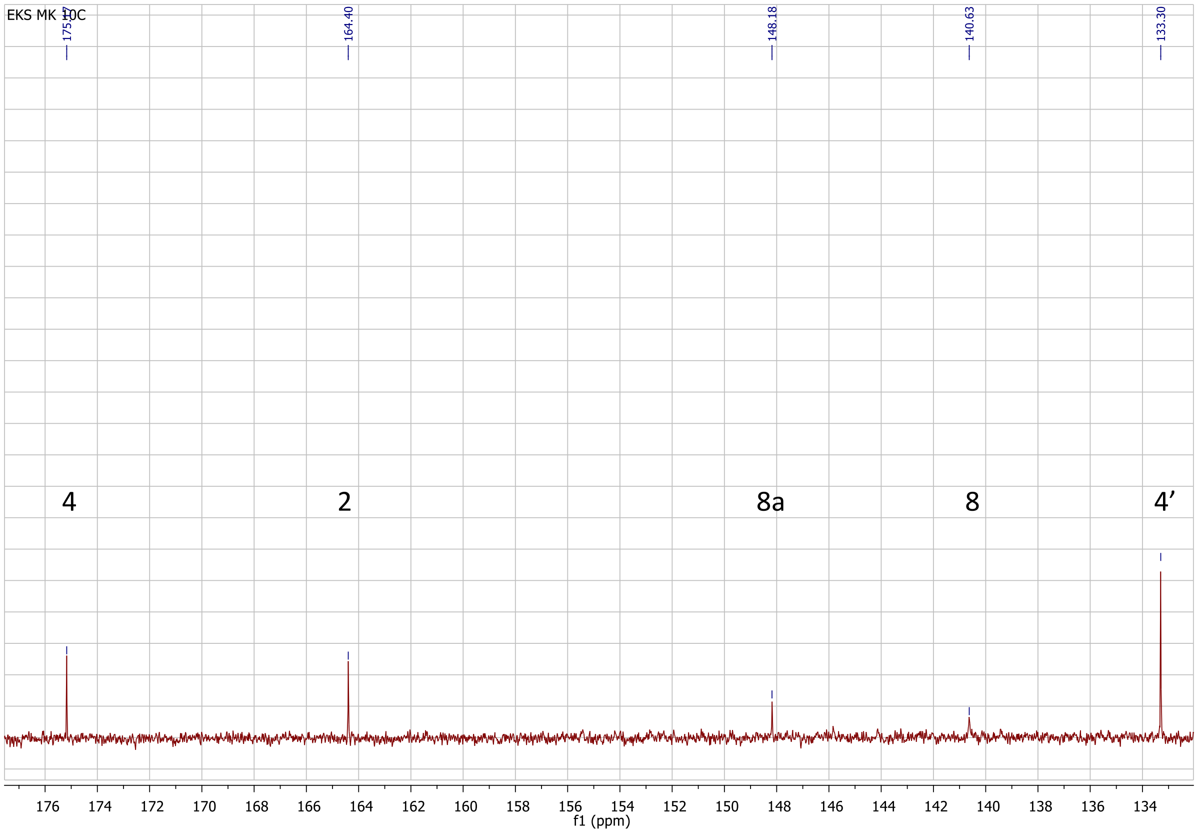

Figure S32. ^{13}*C NMR spectrum expansion of 6-chloro-8-nitroflavone.*

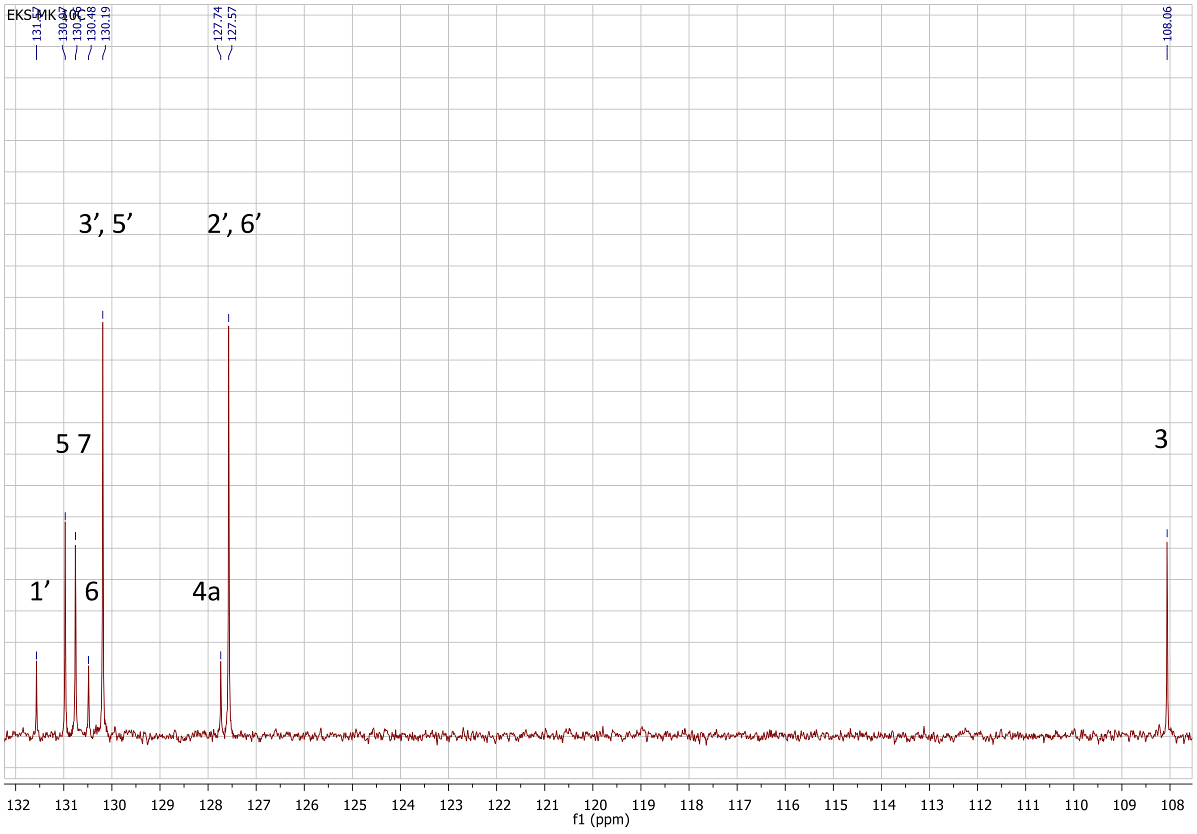

Figure S33. ^{13}*C NMR spectrum expansion of 6-chloro-8-nitroflavone.*

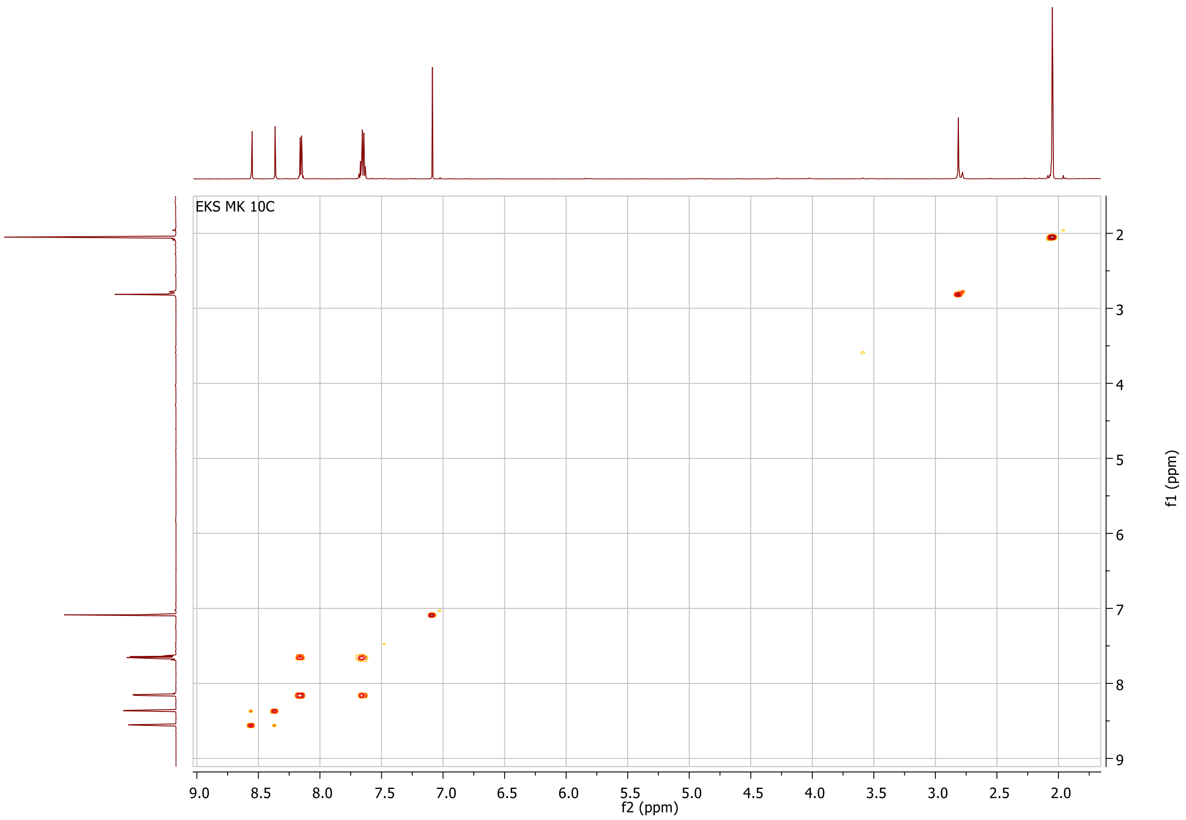

Figure S34. COSY contour map – ¹H x ¹H of 6-chloro-8-nitroflavone.

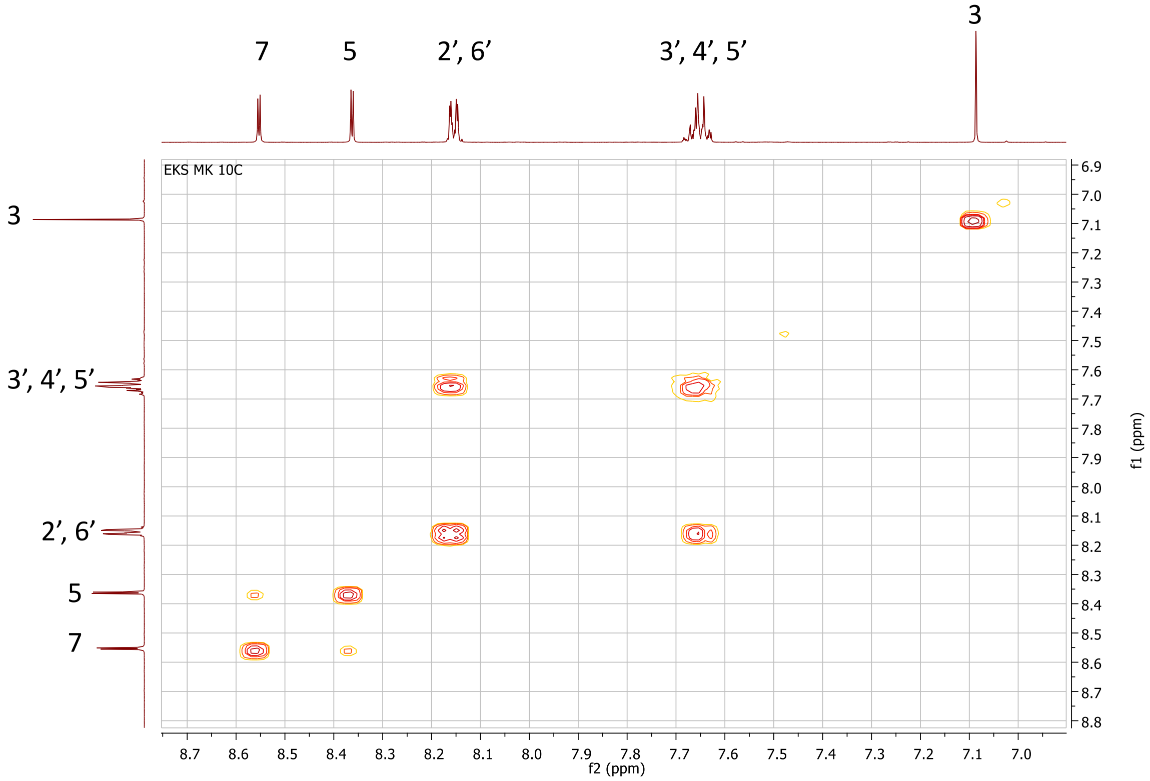

Figure S35. COSY contour map – ¹H x ¹H expansion of 6-chloro-8-nitroflavone.

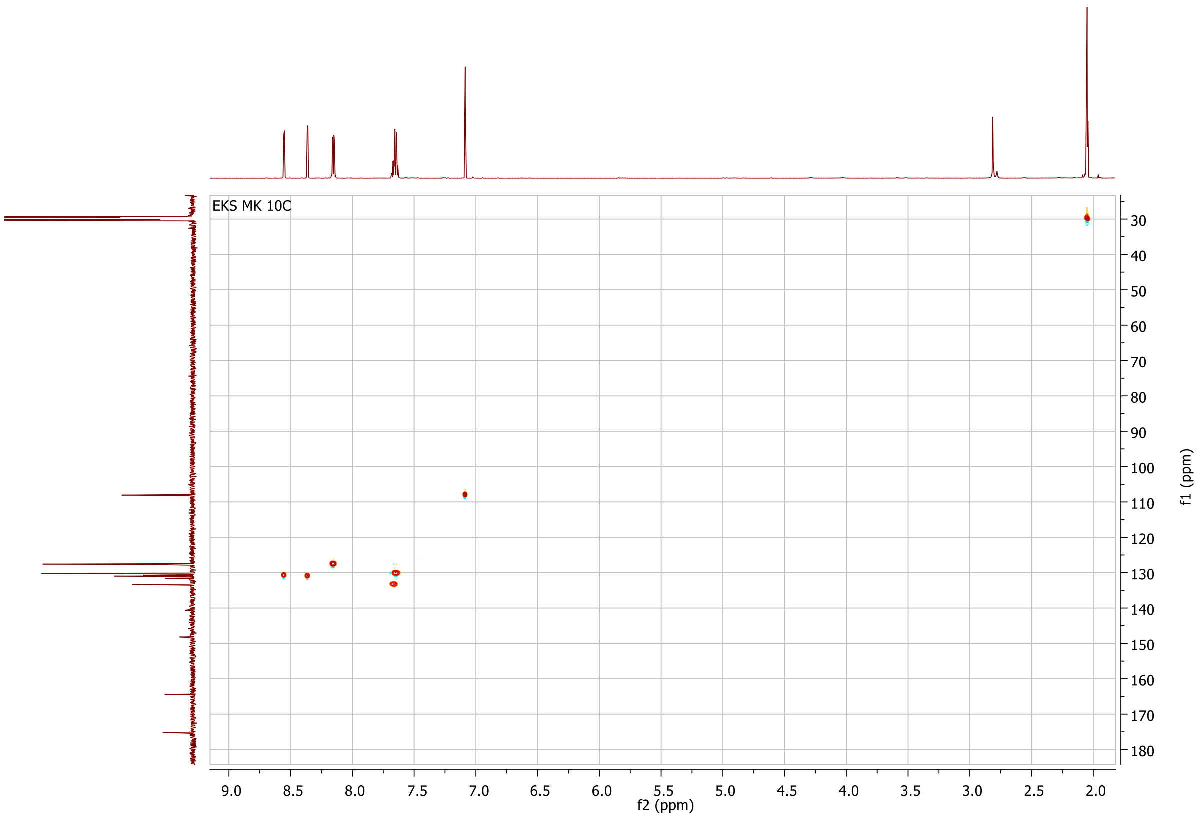

Figure S36. HSQC contour map – ^{1}H x ^{13}C of 6-chloro-8-nitroflavone.

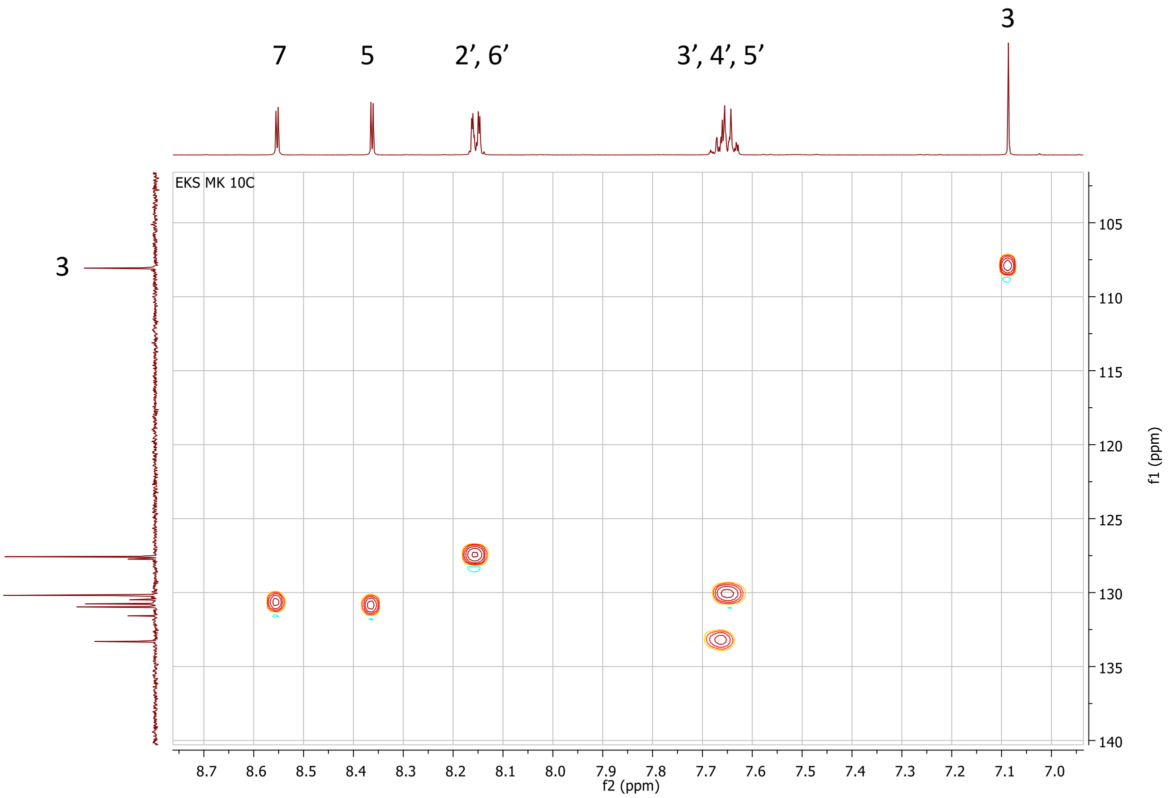

Figure S37. HSQC contour map – ^{1}H x ^{13}C expansion of 6-chloro-8-nitroflavone.

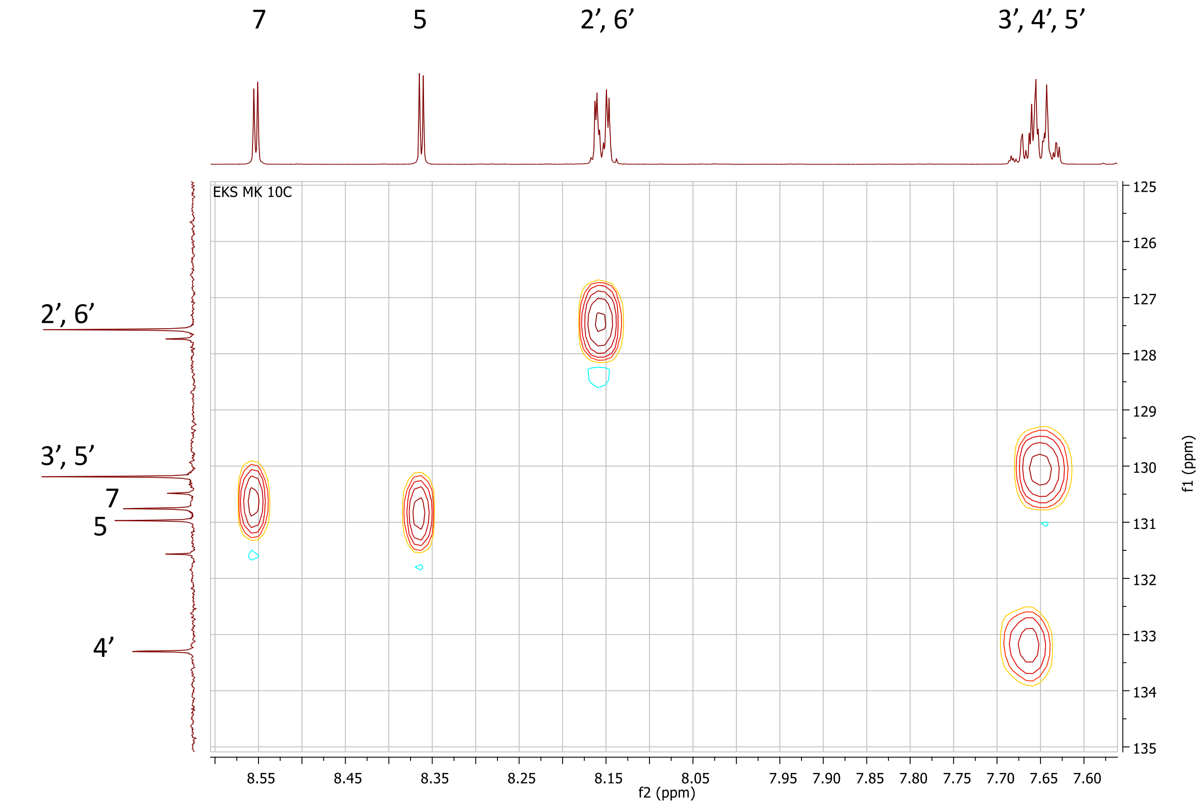

Figure S38. HSQC contour map – ¹H x ¹³C expansion of 6-chloro-8-nitroflavone.

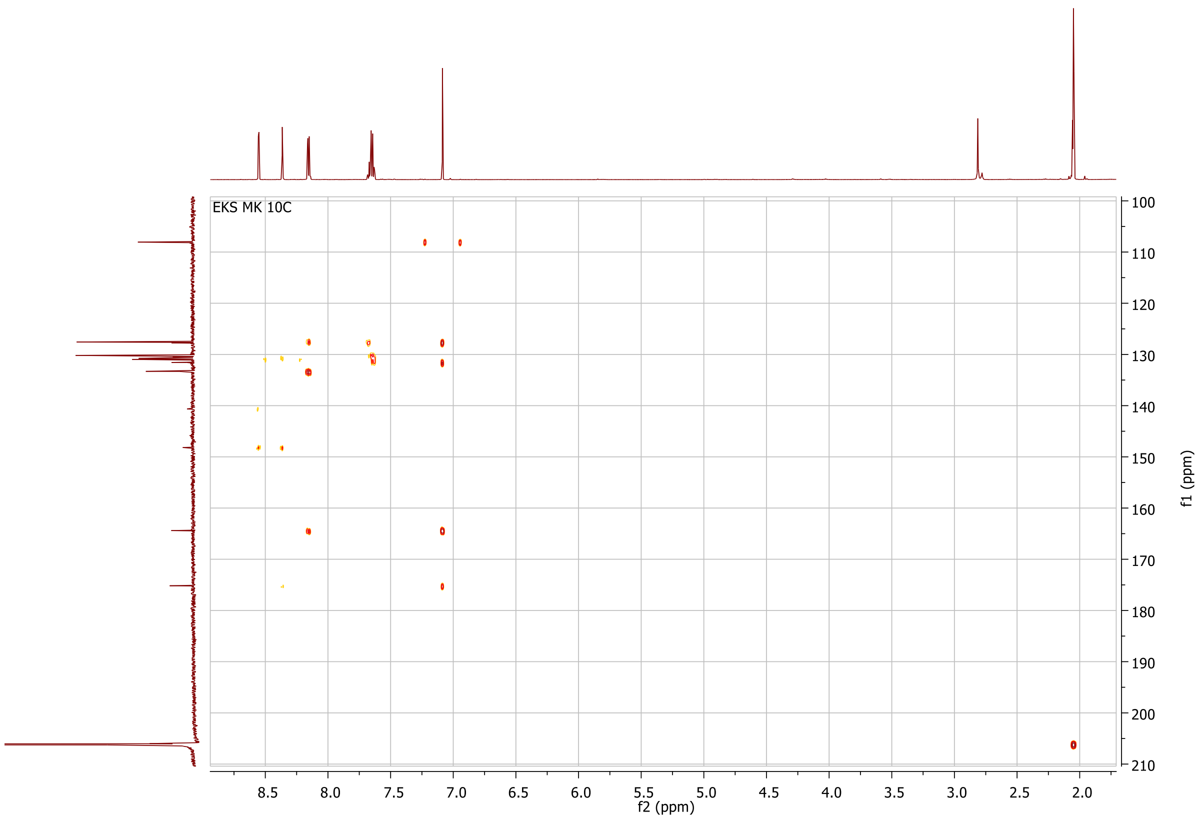

Figure S39. HMBC contour map – ¹H x ¹³C of 6-chloro-8-nitroflavone.

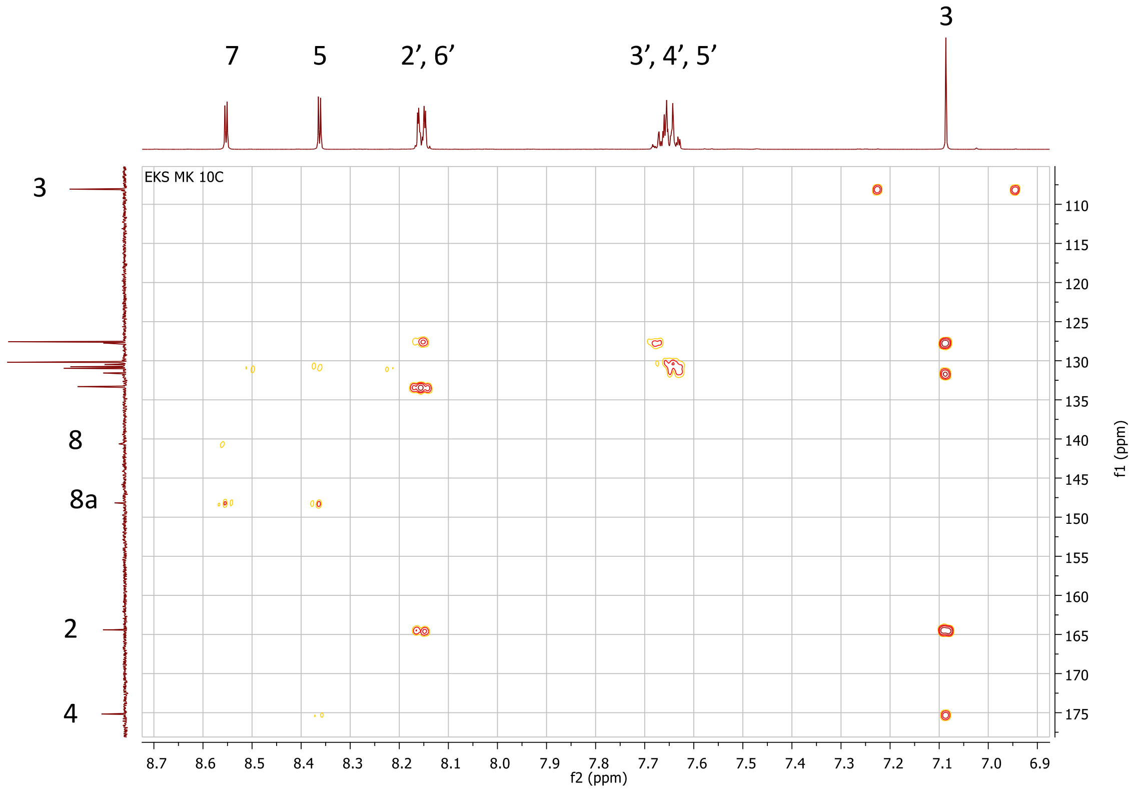

Figure S40. HMBC contour map – ^{1}H x ^{13}C expansion of 6-chloro-8-nitroflavone.

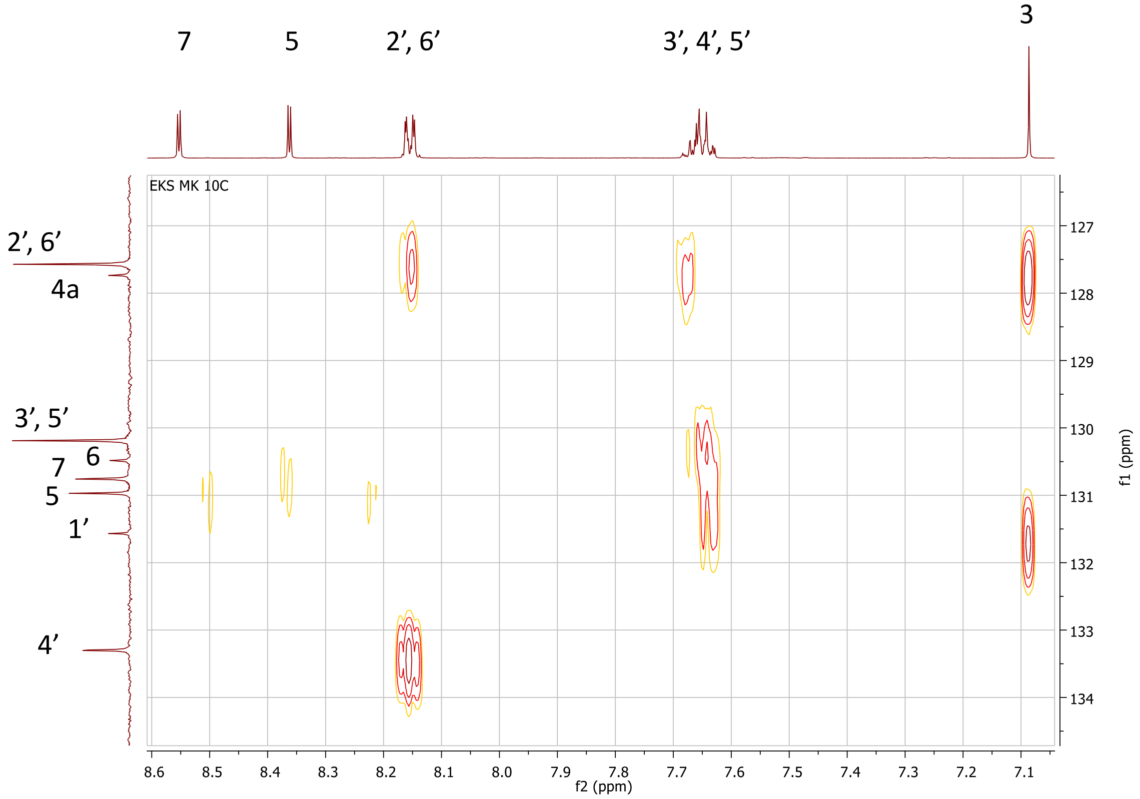

Figure S41. HMBC contour map – ^{1}H x ^{13}C expansion of 6-chloro-8-nitroflavone.

Compound name: 8-amino-6-chloroflavone 4′-*O*-*β*-D-(4″-*O*-methyl)-glucopyranoside
Molecular Formula: $C_{22}H_{22}ClNO_8$
Formula Weight: 463.865
Ionization mode: positive
Precursor $[M + H]^+$: 464.103
Monoisotopic Mass: 463.103
Collision energy (CE): -15.0

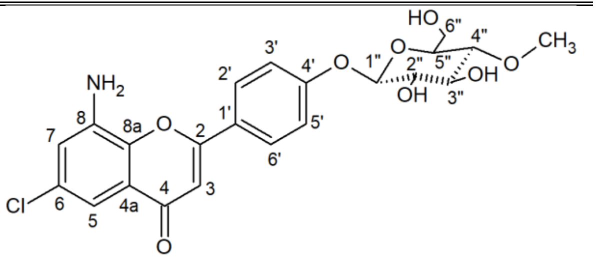

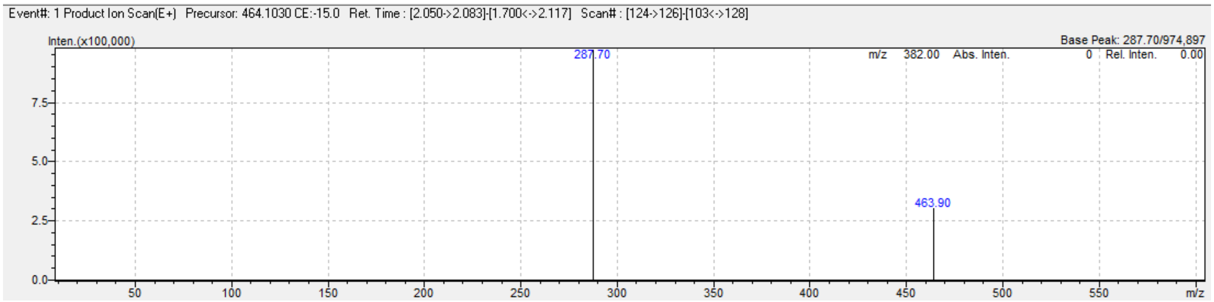

Figure S42. LC-MS spectrum of 8-amino-6-chloroflavone 4′-O-β-D-(4″-O-methyl)-glucopyranoside.

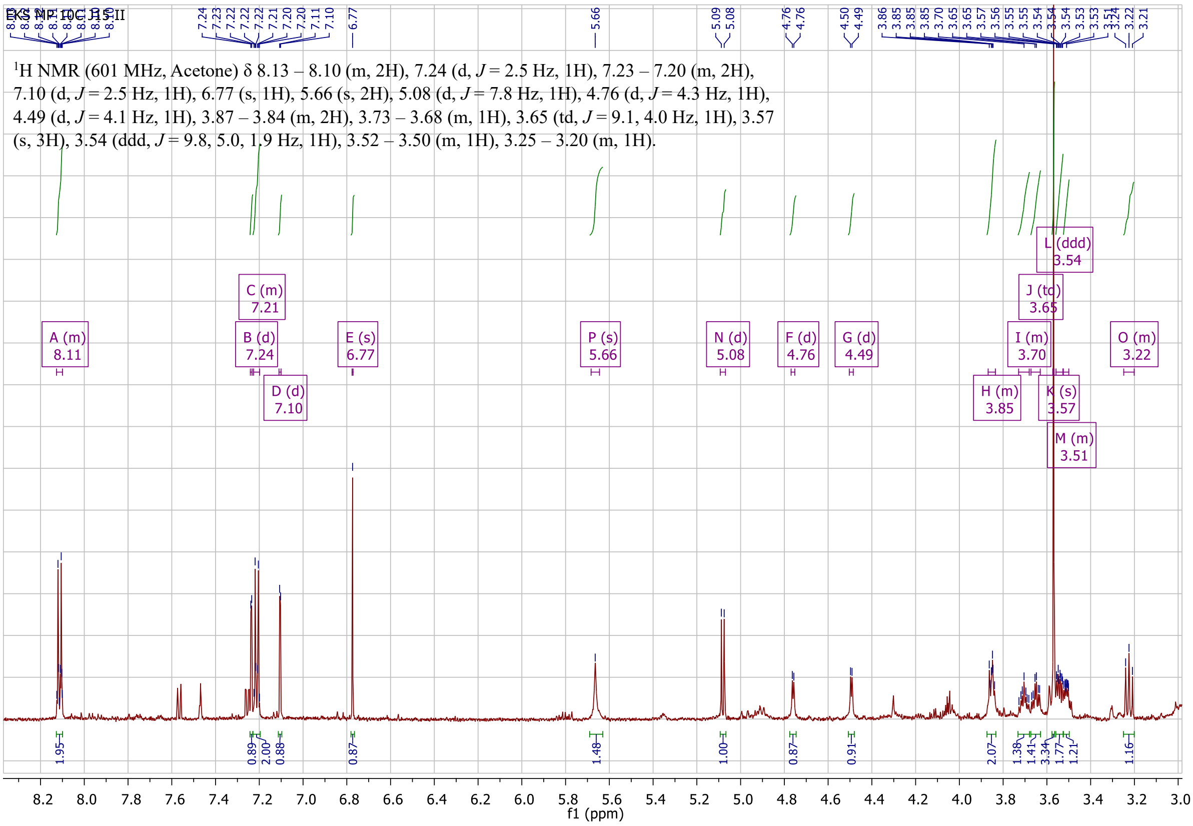

Figure S43. ¹H NMR spectrum of 8-amino-6-chloroflavone 4′-O-β-D-(4″-O-methyl)-glucopyranoside.

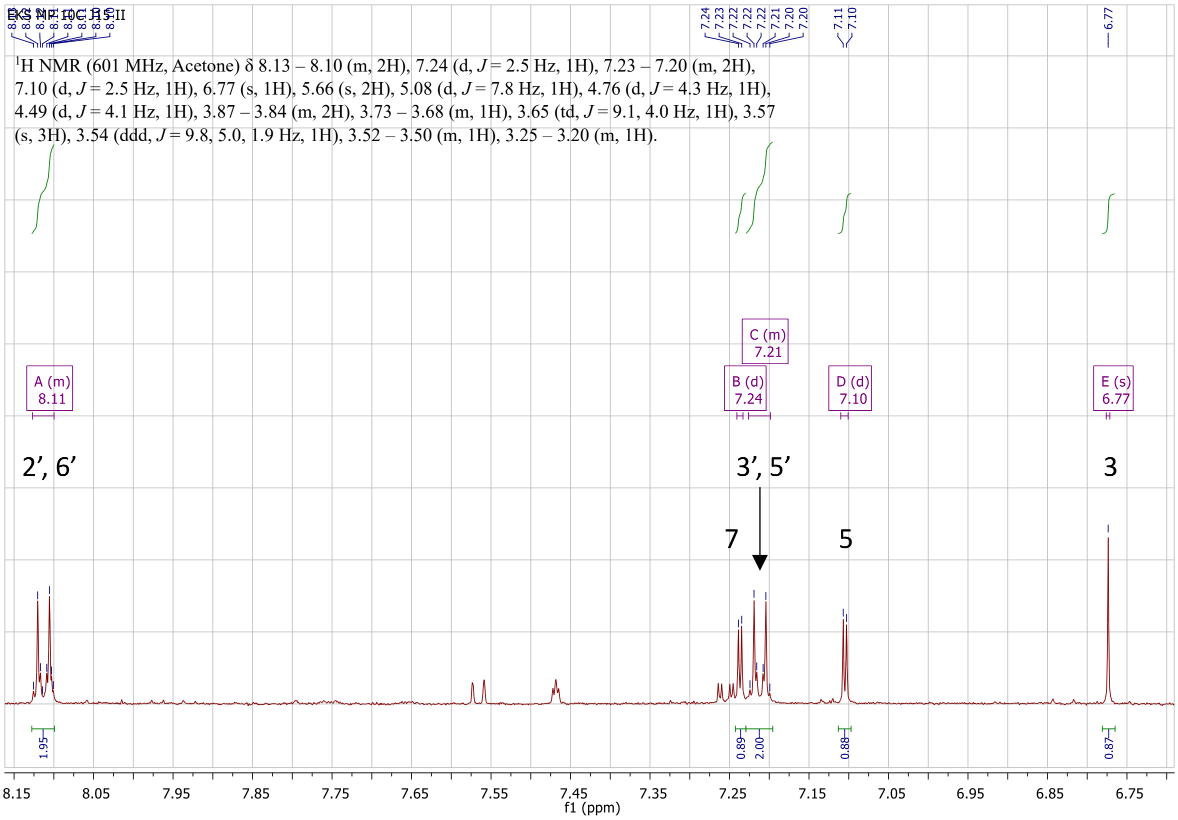

Figure S44. ^{1}H NMR spectrum expansion of 8-amino-6-chloroflavone 4′-O-β-D-(4″-O-methyl)-glucopyranoside.

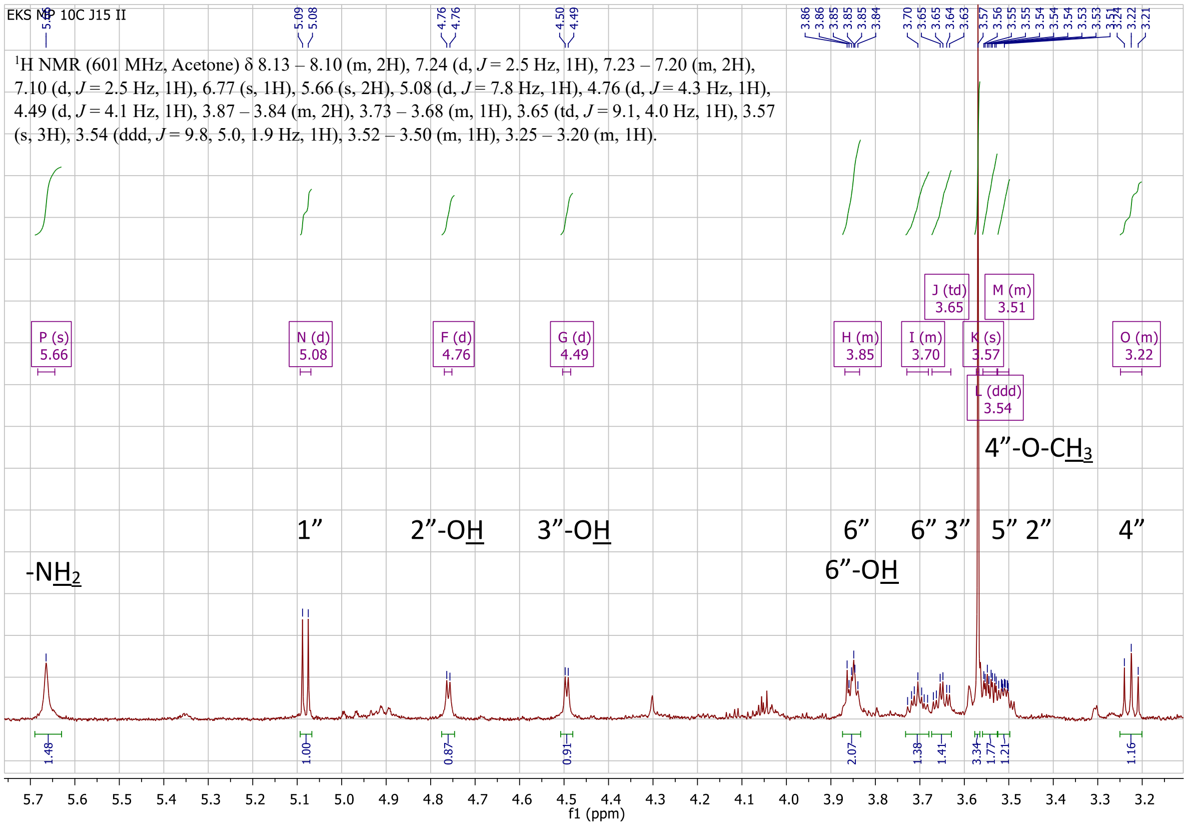

Figure S45. ^{1}H NMR spectrum expansion of 8-amino-6-chloroflavone 4′-O-β-D-(4″-O-methyl)-glucopyranoside.

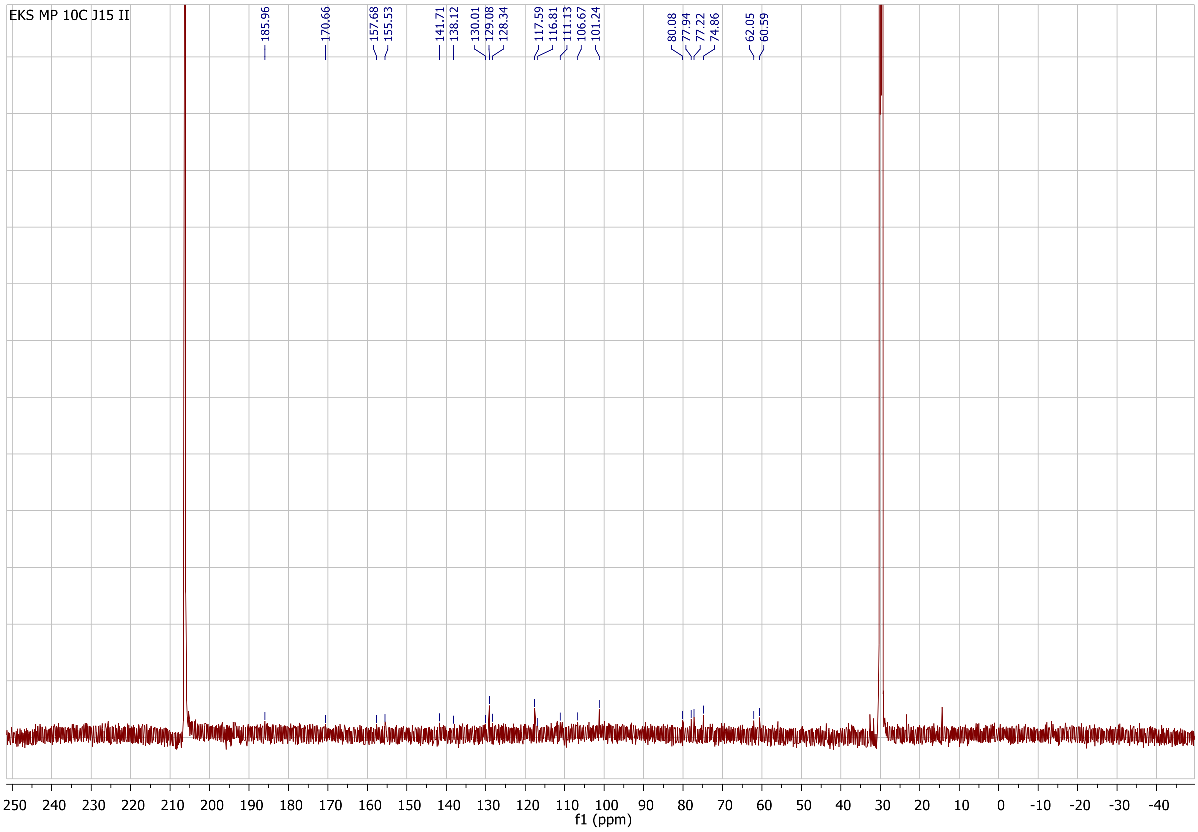

Figure S46. ^{13}C NMR spectrum of 8-amino-6-chloroflavone 4′-O-β-D-(4″-O-methyl)-glucopyranoside.

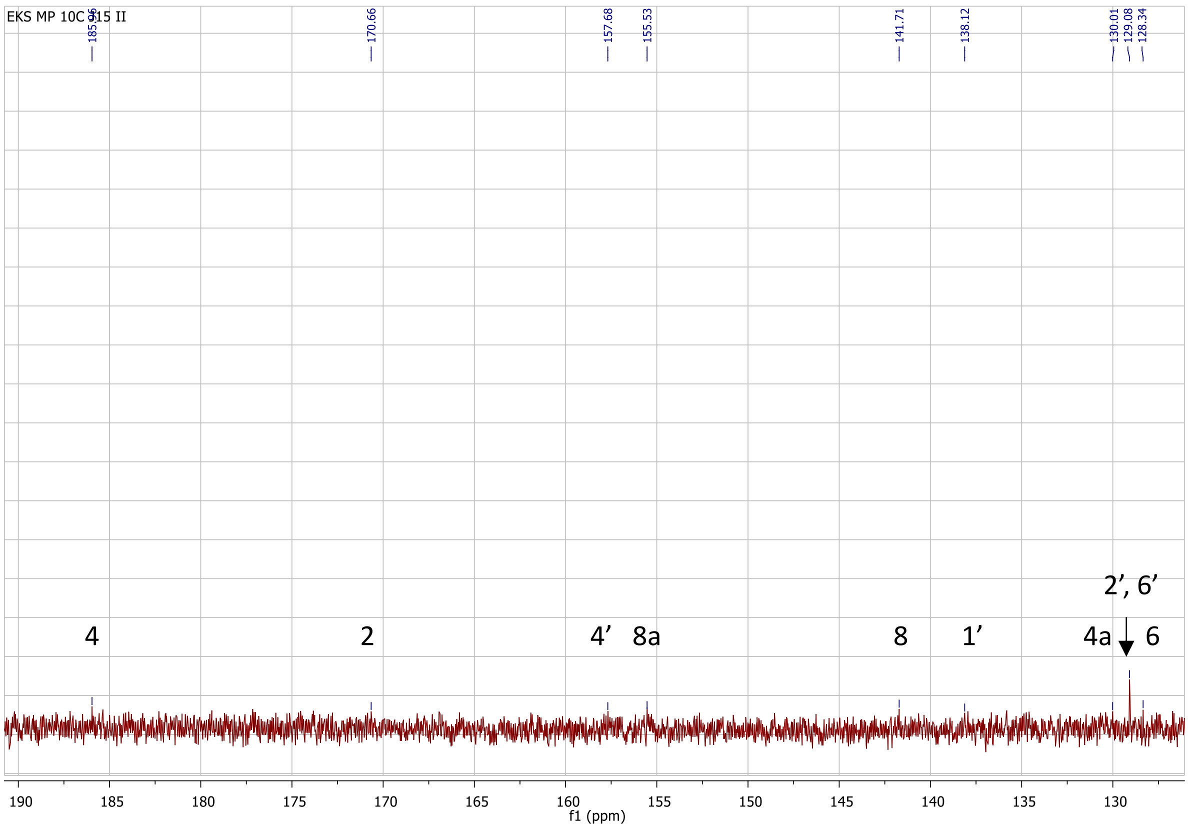

Figure S47. ^{13}C NMR spectrum expansion of 8-amino-6-chloroflavone 4′-O-β-D-(4″-O-methyl)-glucopyranoside.

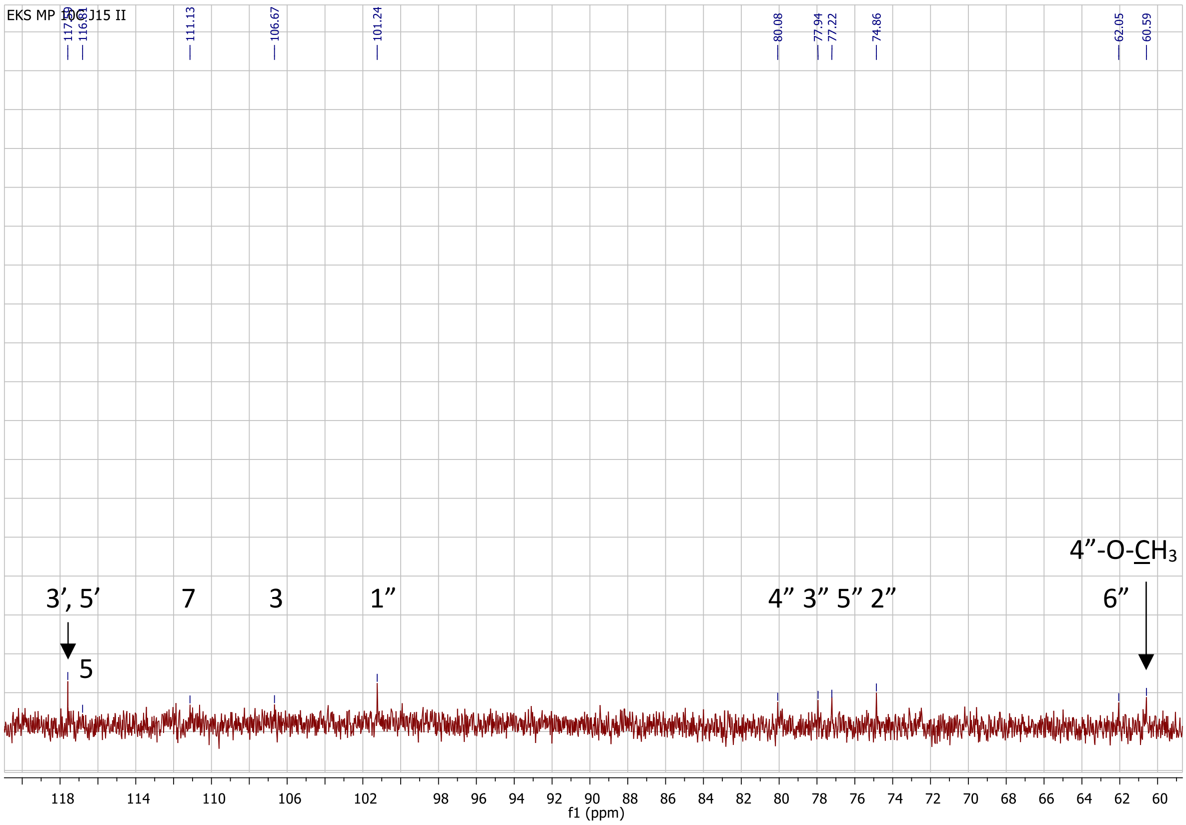

Figure S48. 13C NMR spectrum expansion of 8-amino-6-chloroflavone 4′-O-β-D-(4″-O-methyl)-glucopyranoside.

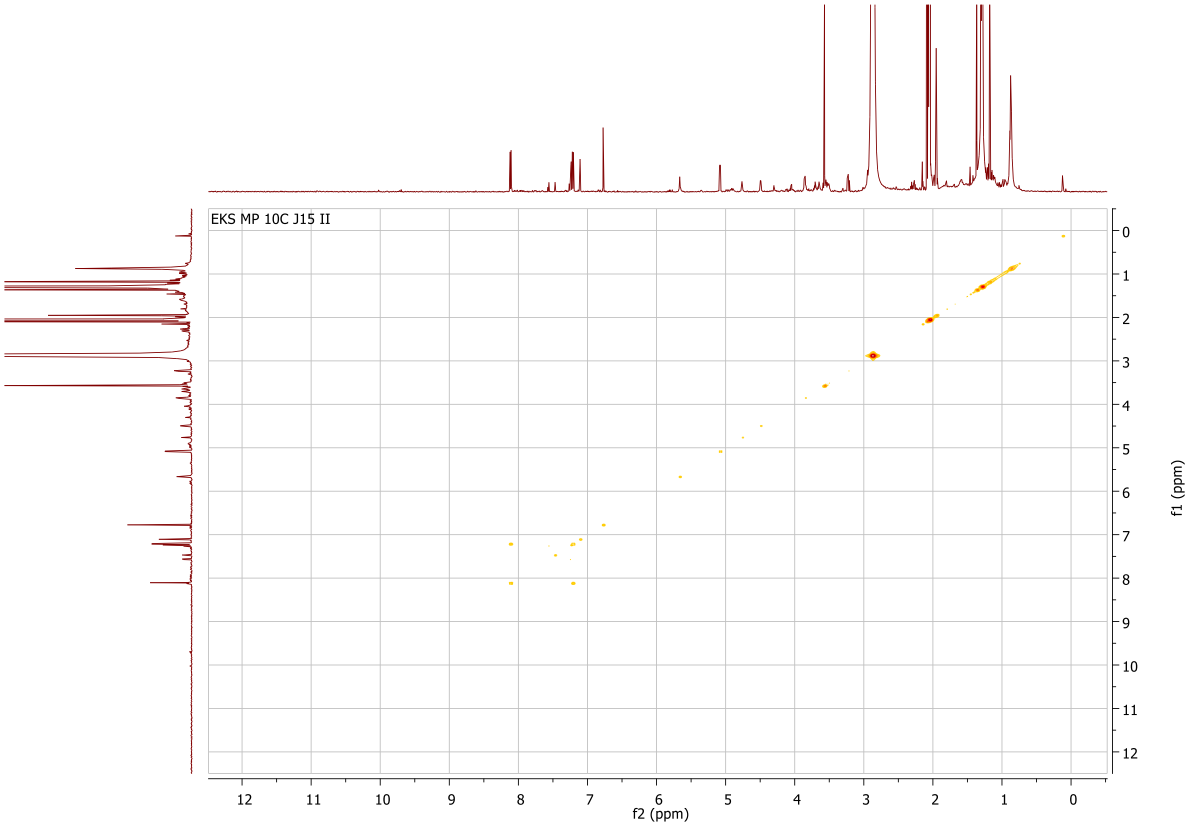

Figure S49. COSY contour map – 1H x 1H of 8-amino-6-chloroflavone 4′-O-β-D-(4″-O-methyl)-glucopyranoside.

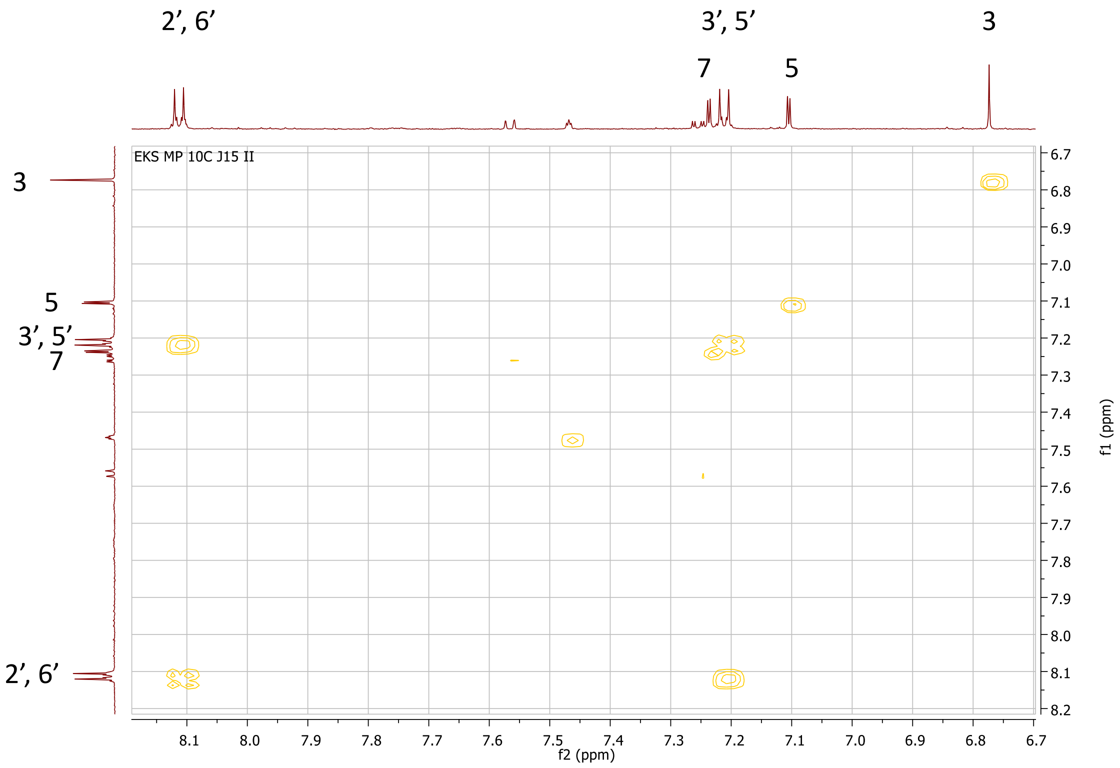

Figure S50. COSY contour map – ¹H x ¹H expansion of 8-amino-6-chloroflavone 4′-O-β-D-(4″-O-methyl)-glucopyranoside.

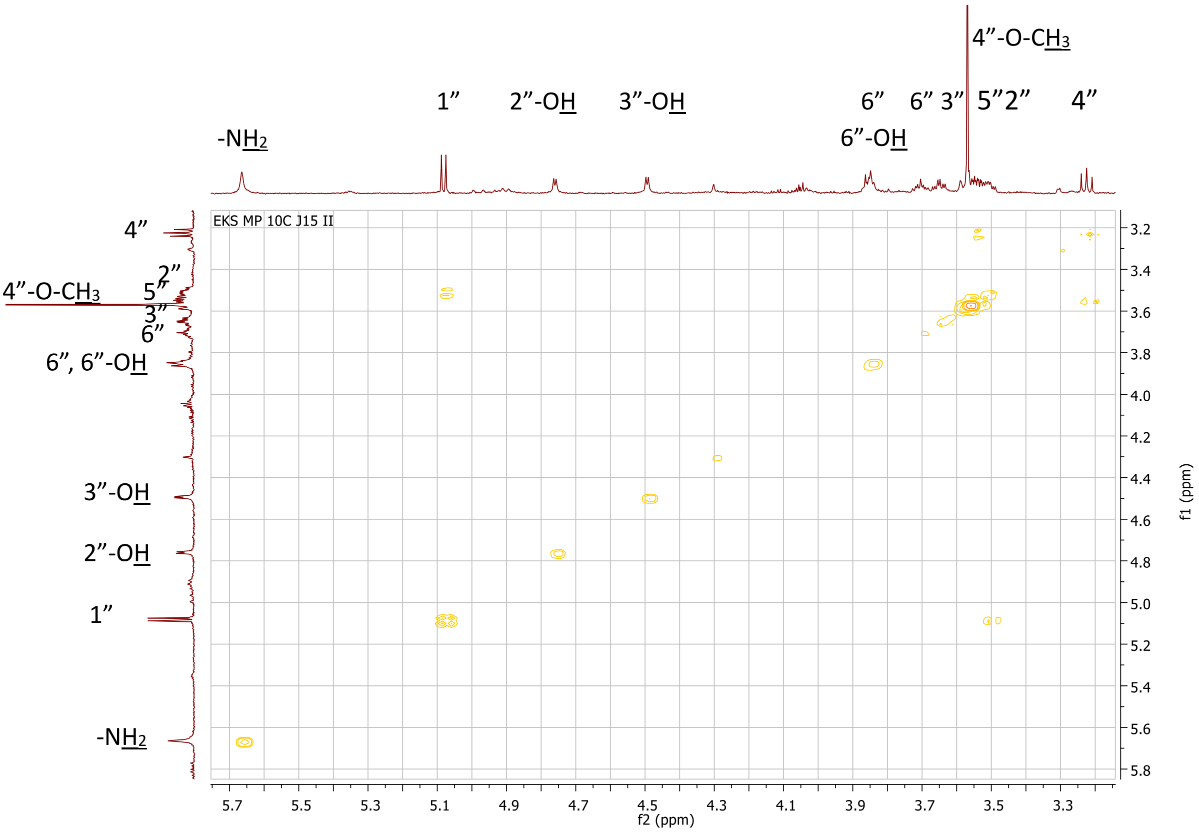

Figure S51. COSY contour map – ¹H x ¹H expansion of 8-amino-6-chloroflavone 4′-O-β-D-(4″-O-methyl)-glucopyranoside.

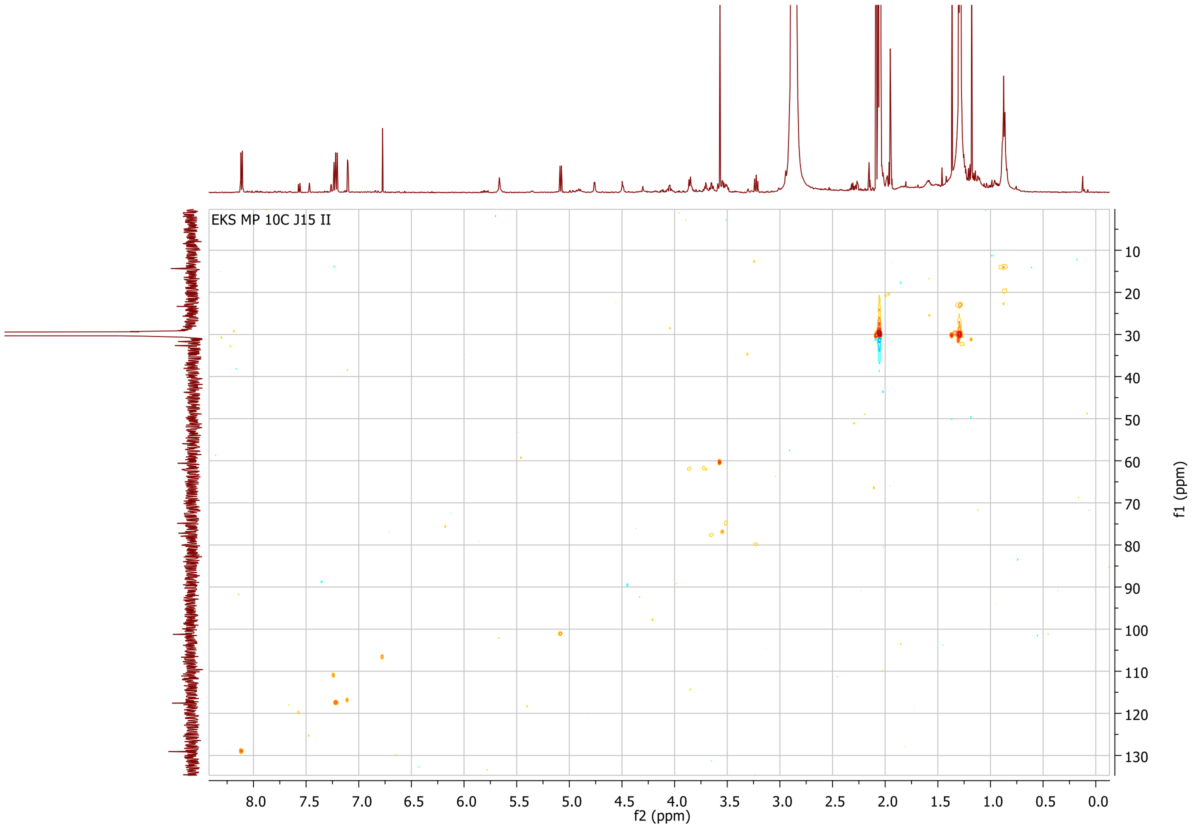

Figure S52. HSQC contour map – ^{1}H x ^{13}C of 8-amino-6-chloroflavone 4′-O-β-D-(4″-O-methyl)-glucopyranoside.

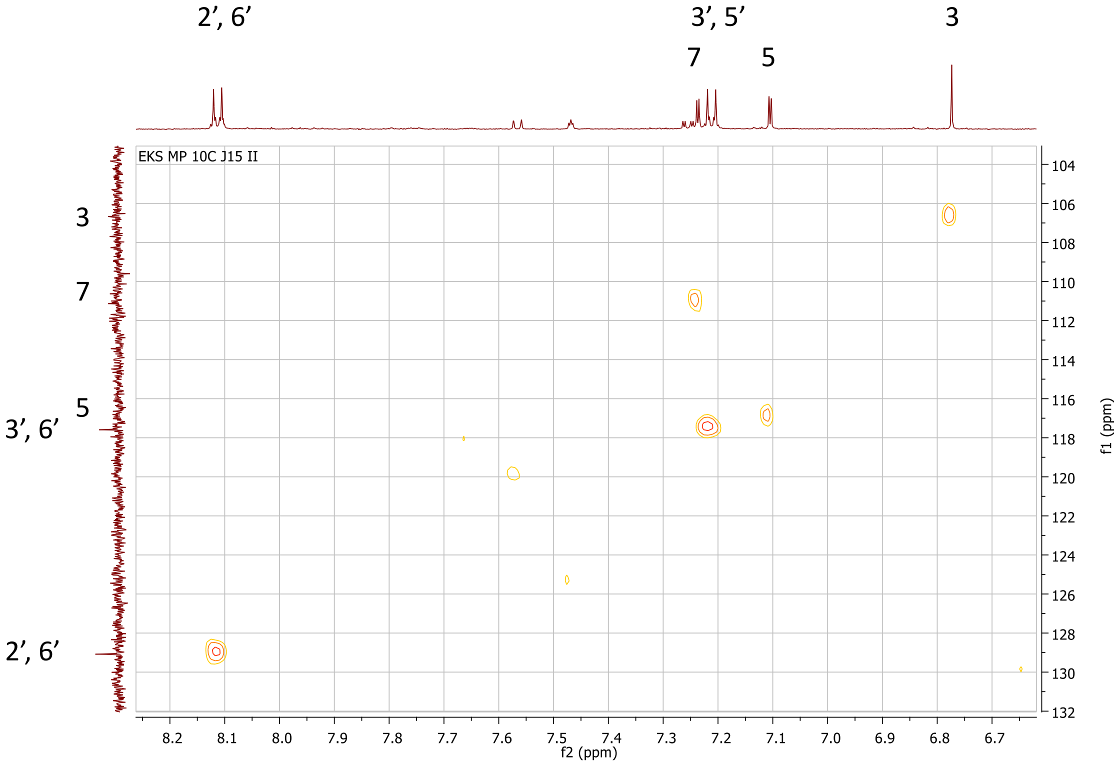

Figure S53. HSQC contour map – ^{1}H x ^{13}C expansion of 8-amino-6-chloroflavone 4′-O-β-D-(4″-O-methyl)-glucopyranoside.

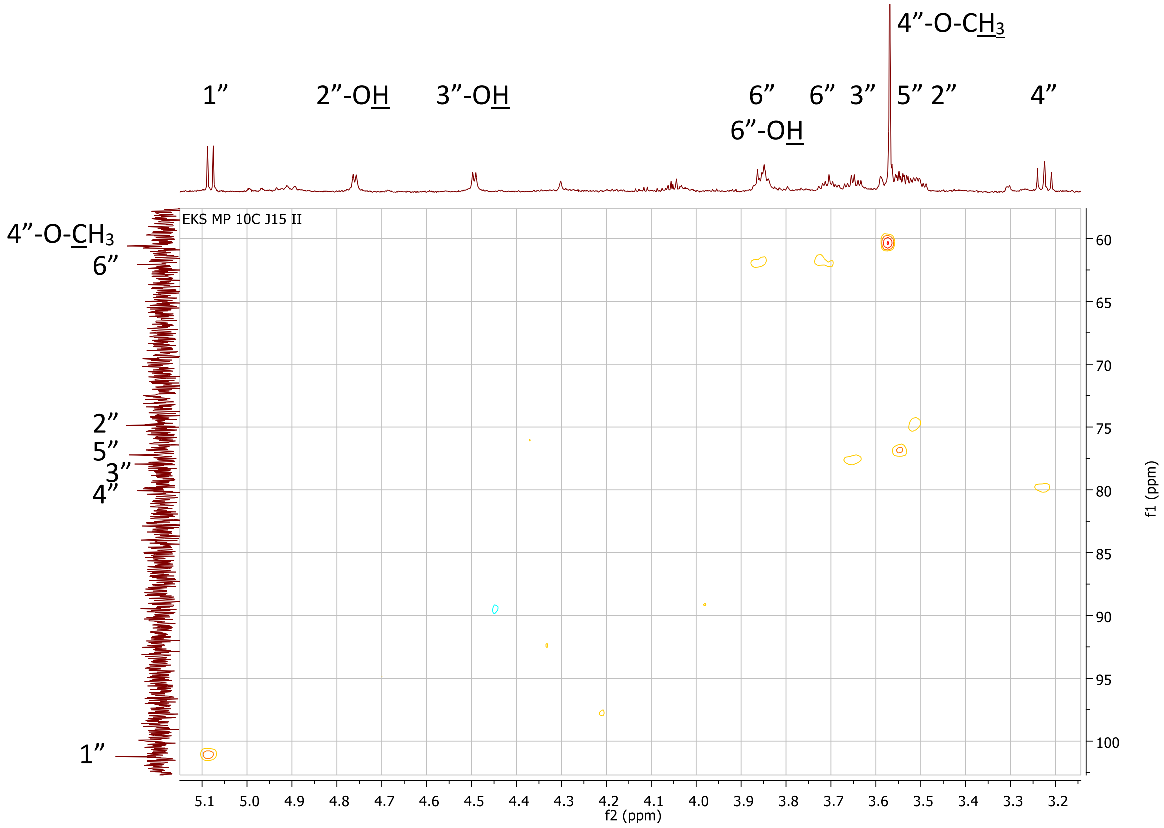

Figure S54. HSQC contour map – ^{1}H x ^{13}C expansion of 8-amino-6-chloroflavone 4′-O-β-D-(4″-O-methyl)-glucopyranoside.

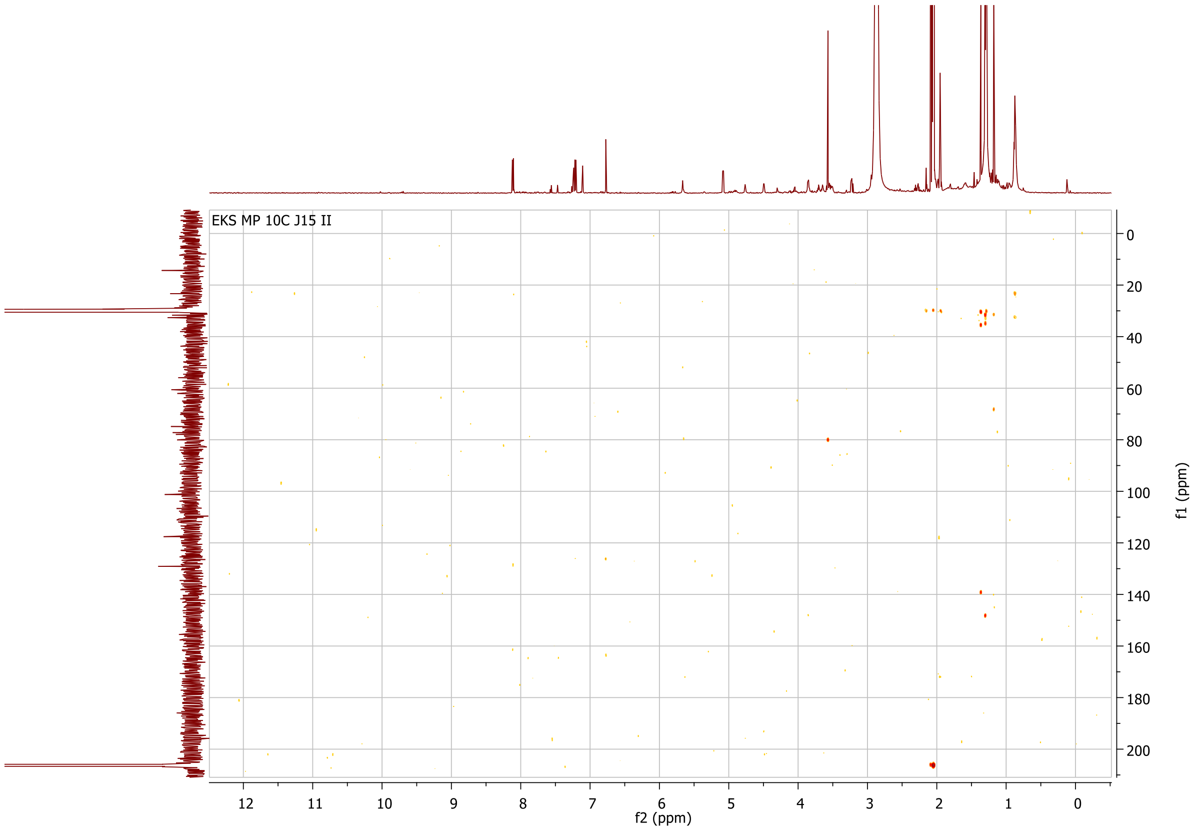

Figure S55. HMBC contour map – ^{1}H x ^{13}C of 8-amino-6-chloroflavone 4′-O-β-D-(4″-O-methyl)-glucopyranoside.

Compound name: 5′-bromo-2′-hydroxy-3′-nitrochalcone
Molecular Formula: $C_{15}H_{10}BrNO_4$
Formula Weight: 348.148
Ionization mode: positive
Precursor $[M + H]^+$: 347.979
Monoisotopic Mass: 346.979
Collision energy (CE): -15.0

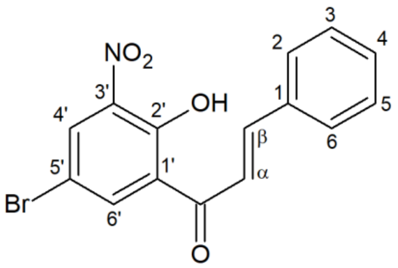

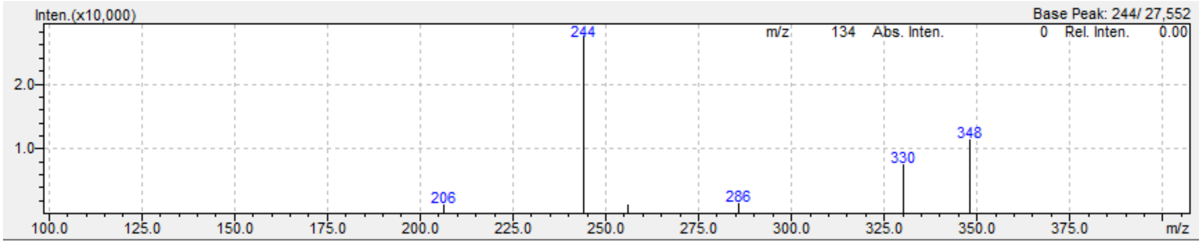

Figure S56. LC-MS spectrum of 5′-bromo-2′-hydroxy-3′-nitrochalcone.

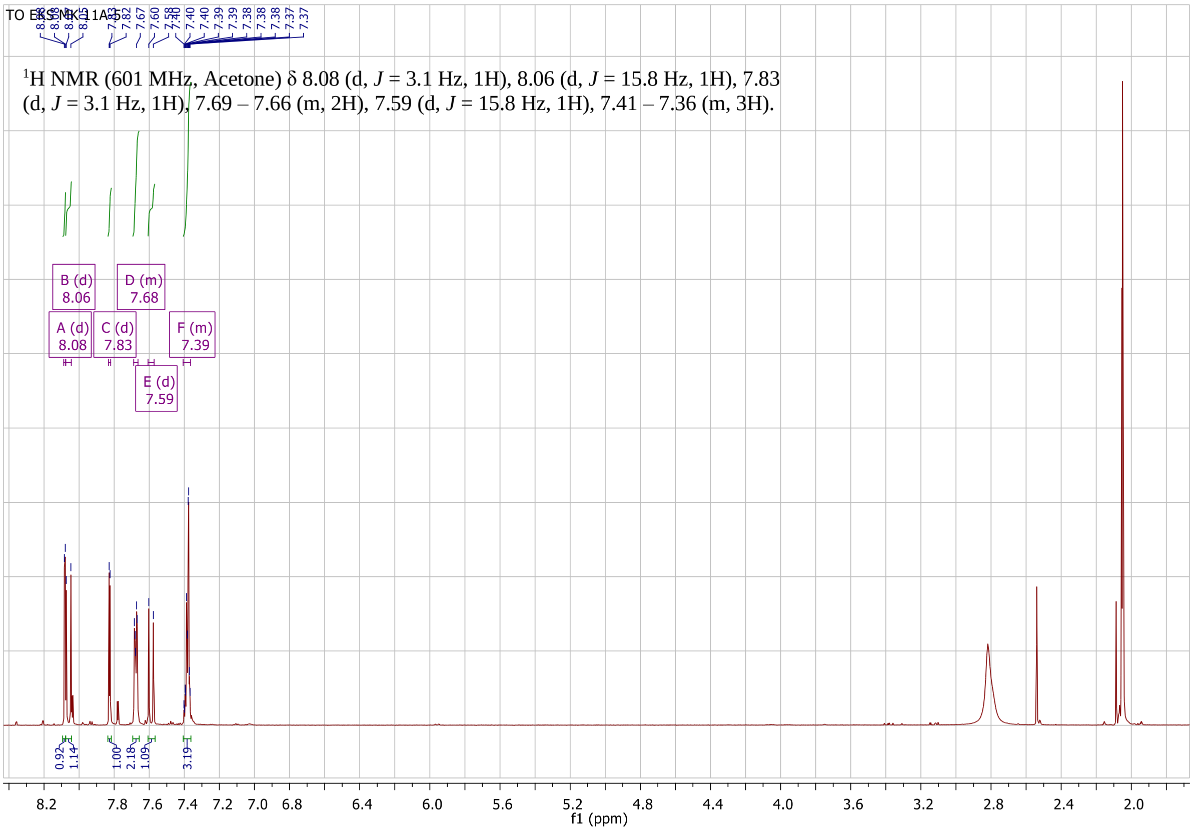

Figure S57. ¹H NMR spectrum of 5′-bromo-2′-hydroxy-3′-nitrochalcone.

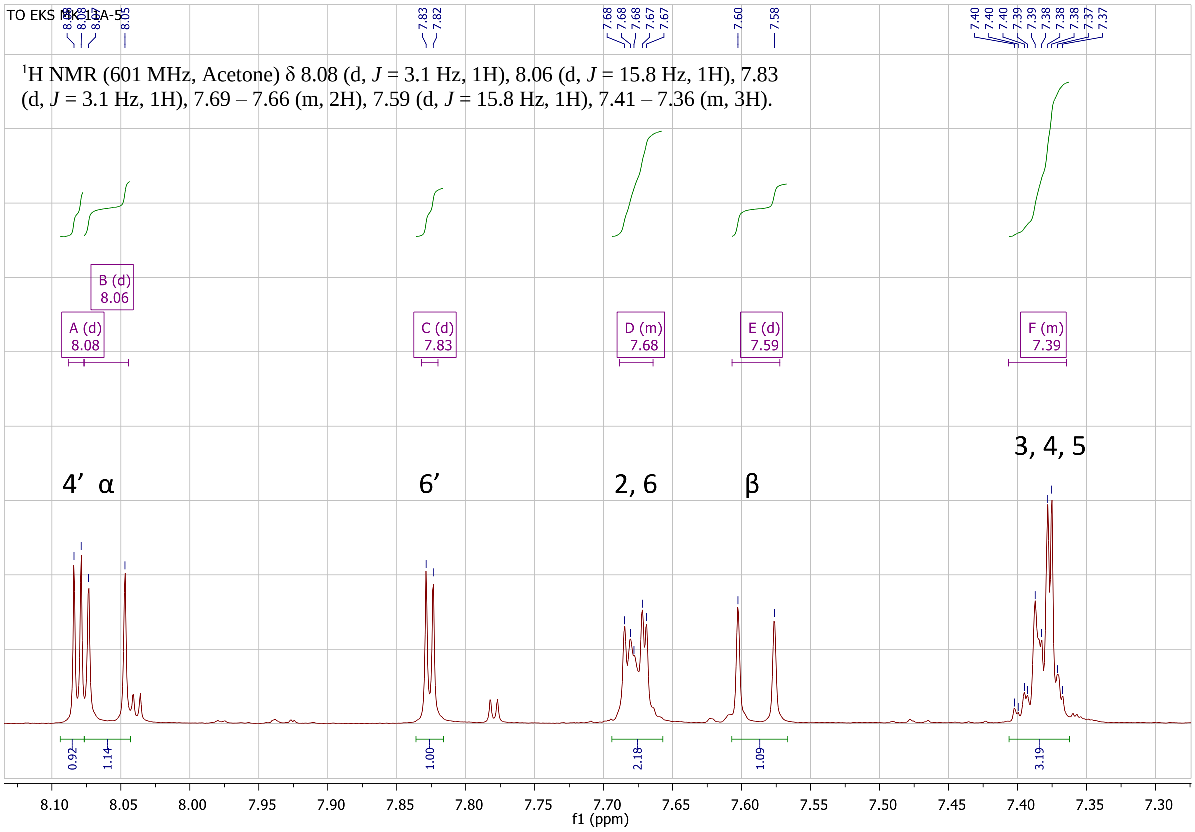

Figure S58. ¹H NMR spectrum expansion of 5′-bromo-2′-hydroxy-3′-nitrochalcone.

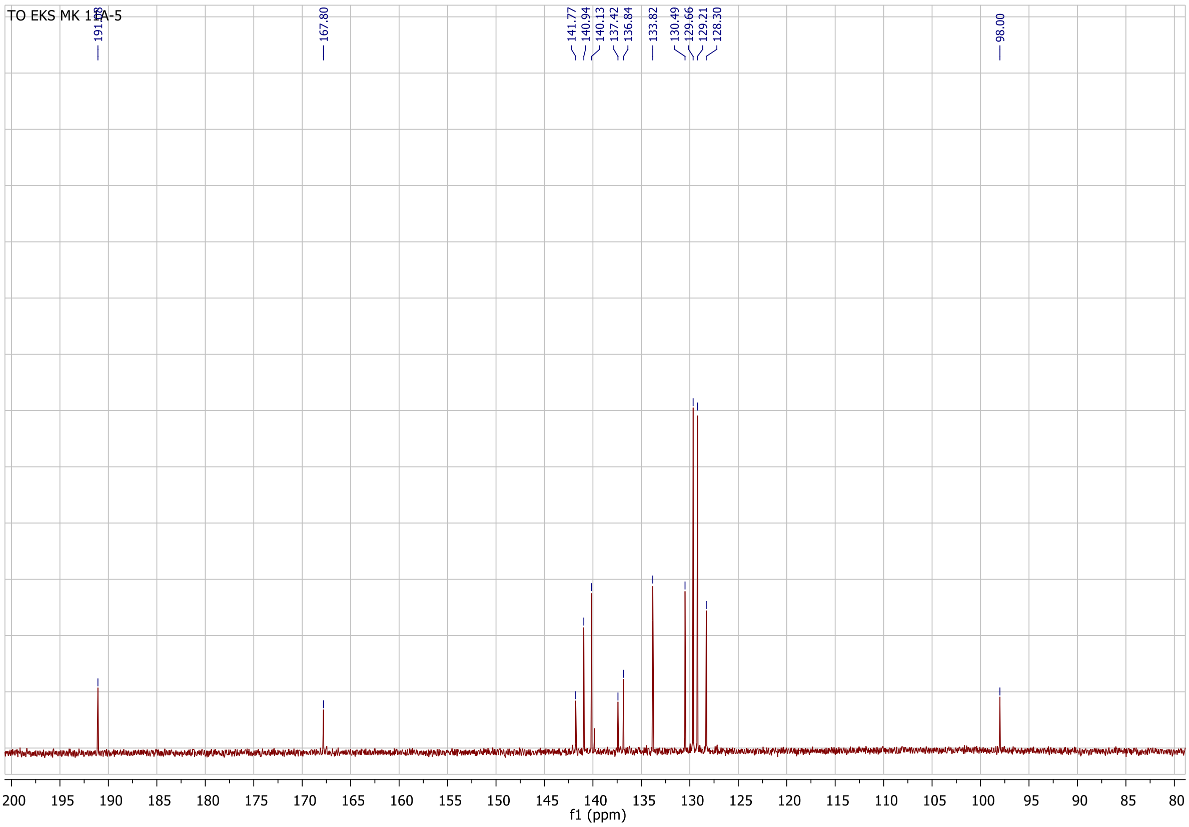

Figure S59. ¹³C NMR spectrum of 5′-bromo-2′-hydroxy-3′-nitrochalcone.

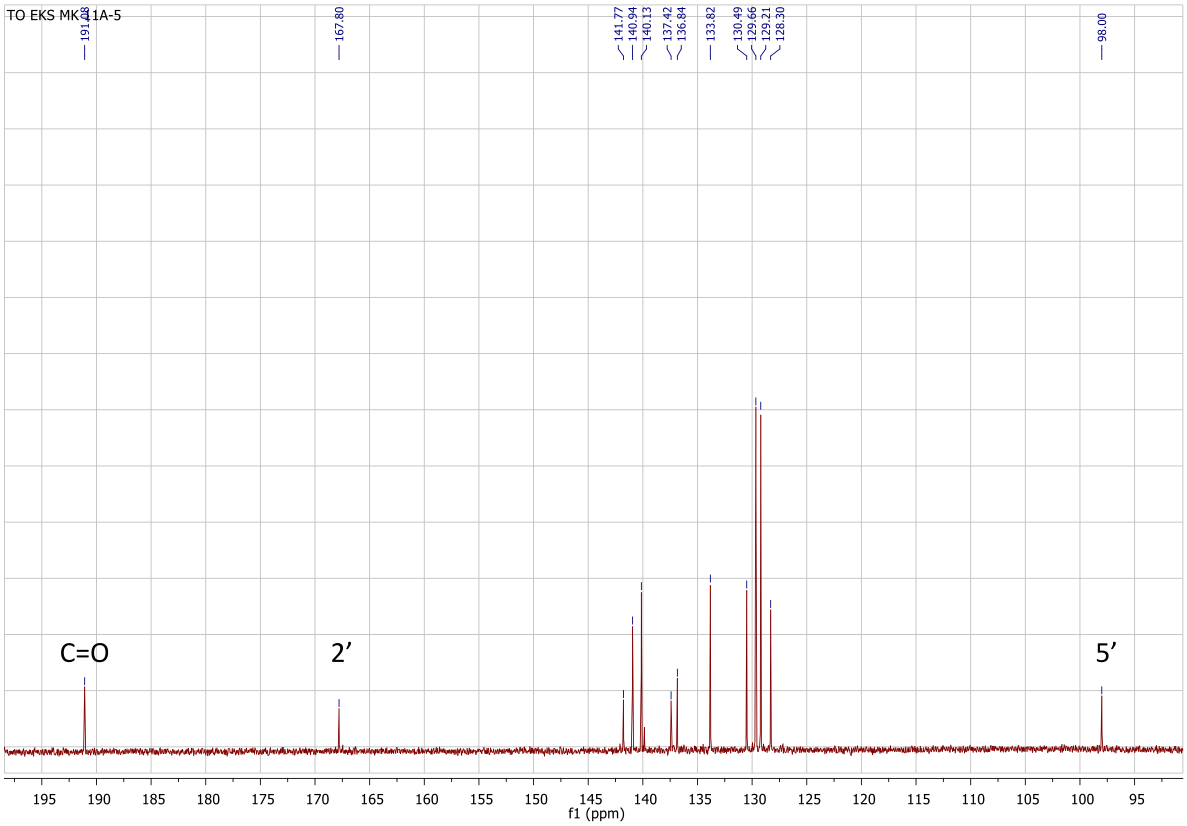

Figure S60. ^{13}*C NMR spectrum expansion of 5′-bromo-2′-hydroxy-3′-nitrochalcone.*

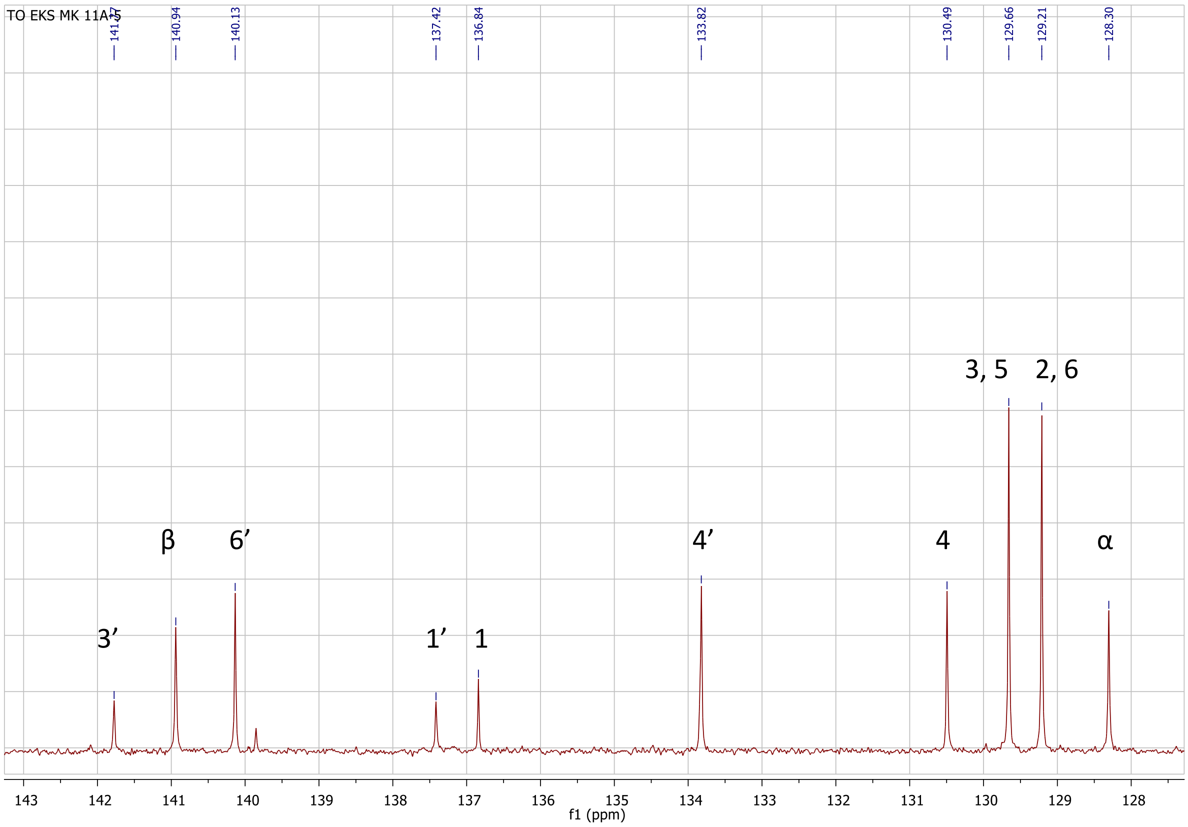

Figure S61. ^{13}*C NMR spectrum expansion of 5′-bromo-2′-hydroxy-3′-nitrochalcone.*

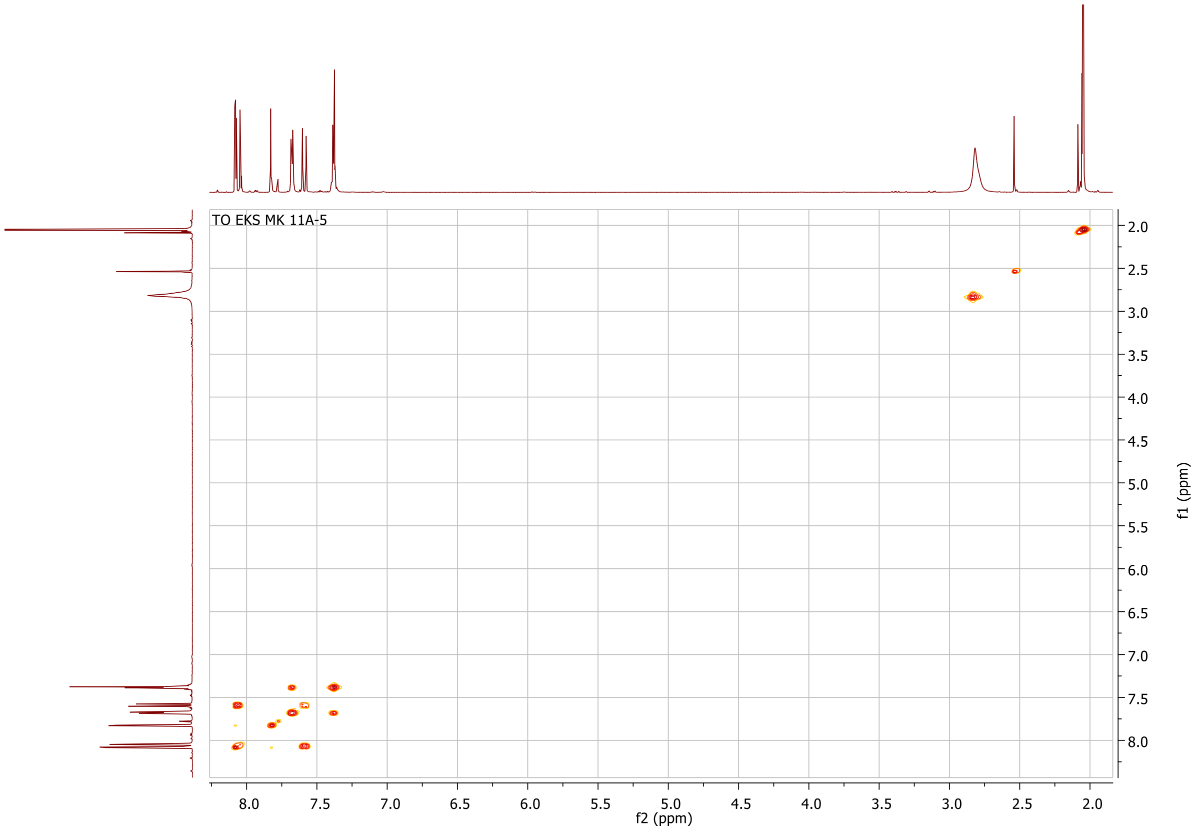

Figure S62. COSY contour map – ¹H x ¹H of 5′-bromo-2′-hydroxy-3′-nitrochalcone.

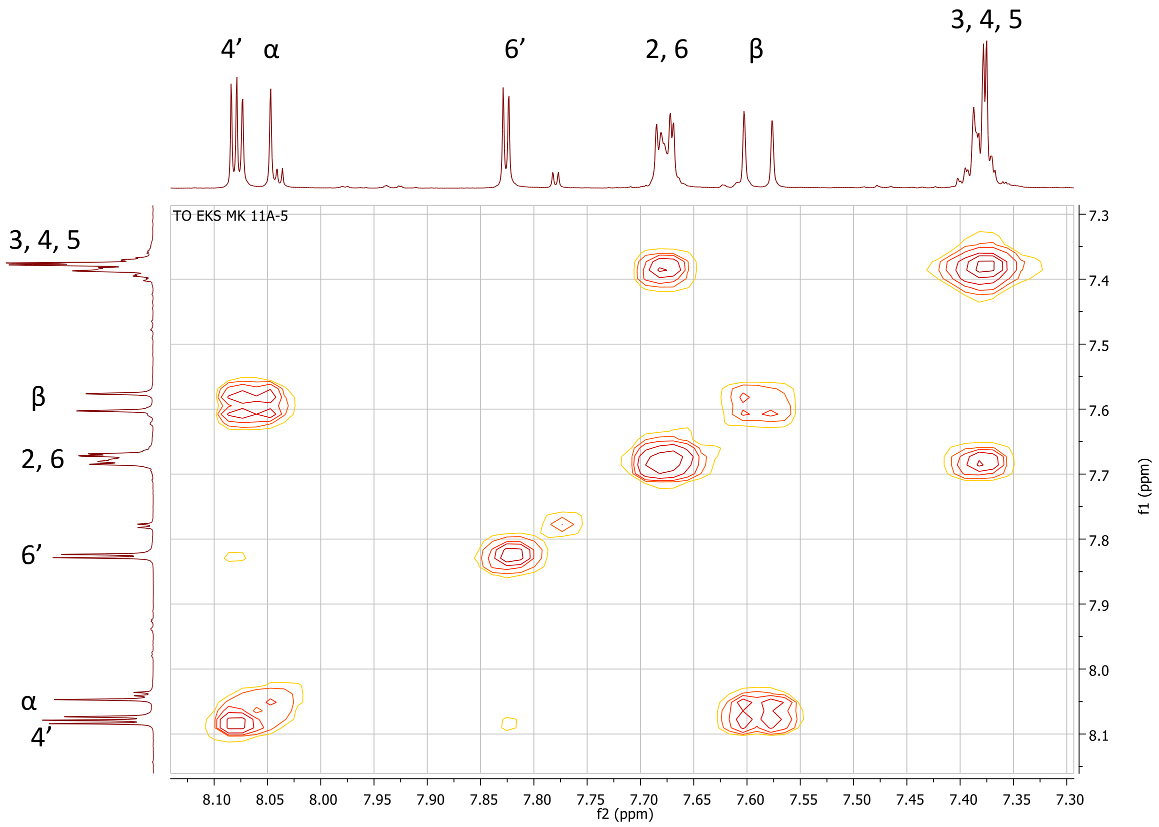

Figure S20. COSY contour map – ¹H x ¹H expansion of 5′-bromo-2′-hydroxy-3′-nitrochalcone.

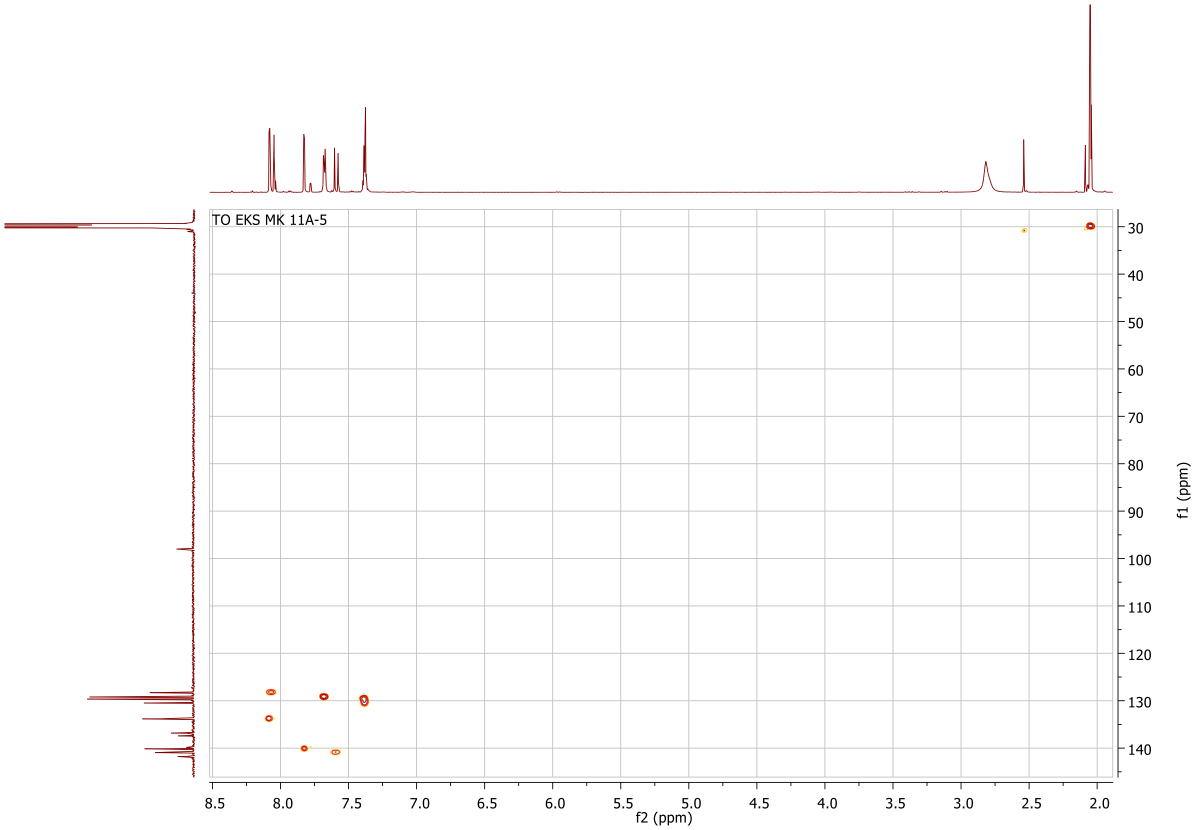

Figure S64. HSQC contour map – ^{1}H x ^{13}C of 5′-bromo-2′-hydroxy-3′-nitrochalcone.

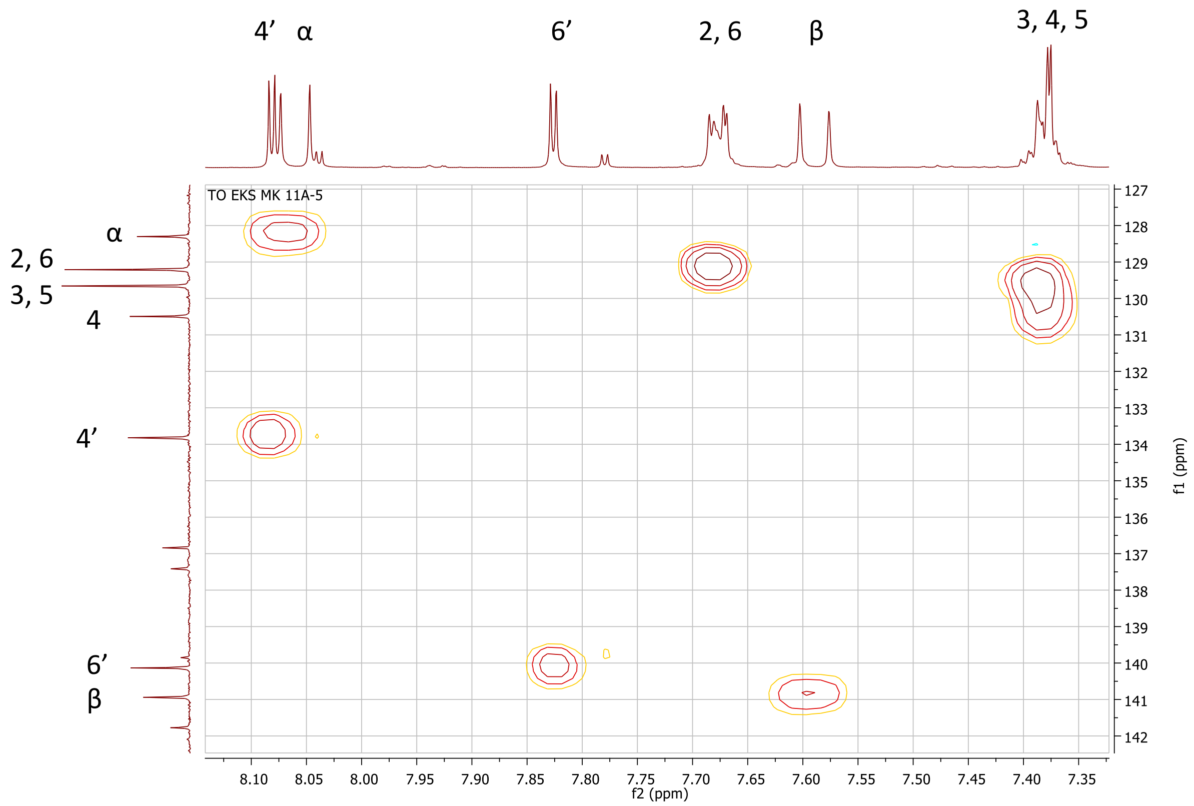

Figure S65. HSQC contour map – ^{1}H x ^{13}C expansion of 5′-bromo-2′-hydroxy-3′-nitrochalcone.

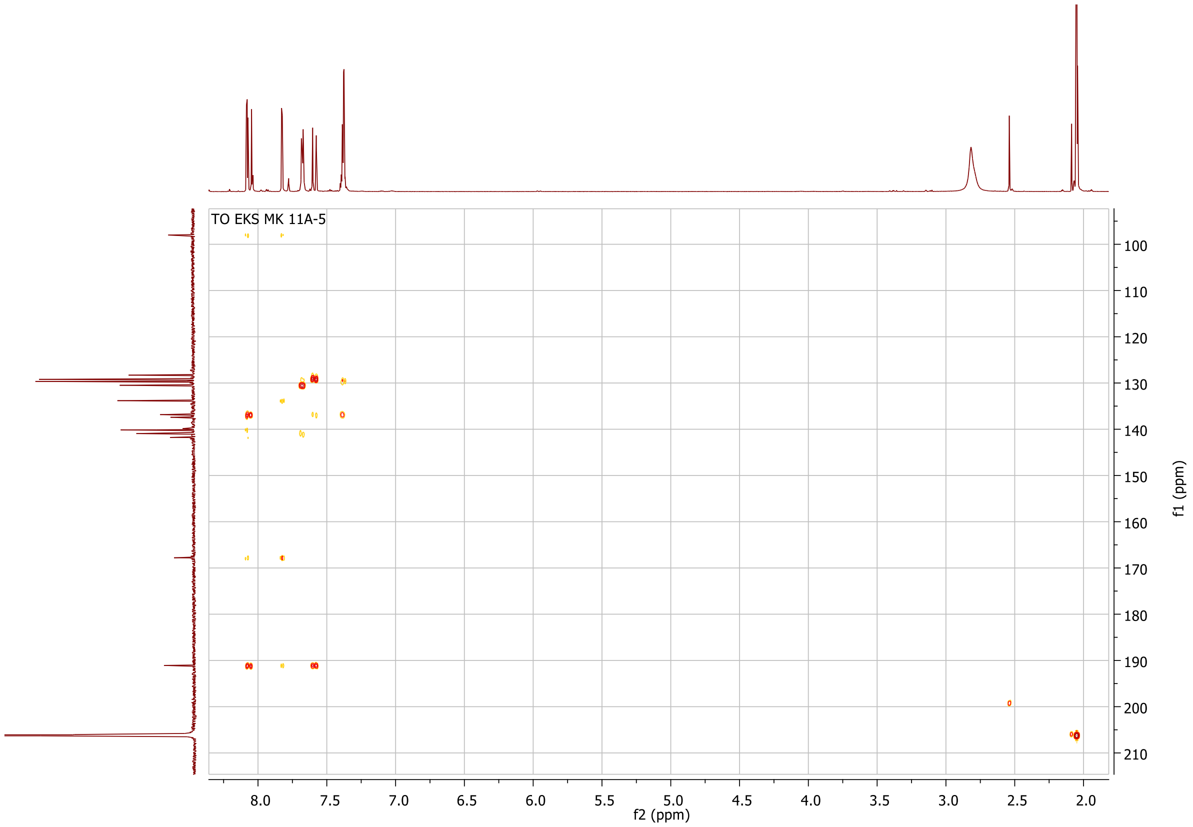

Figure S66. HMBC contour map – ^{1}H x ^{13}C of 5′-bromo-2′-hydroxy-3′-nitrochalcone.

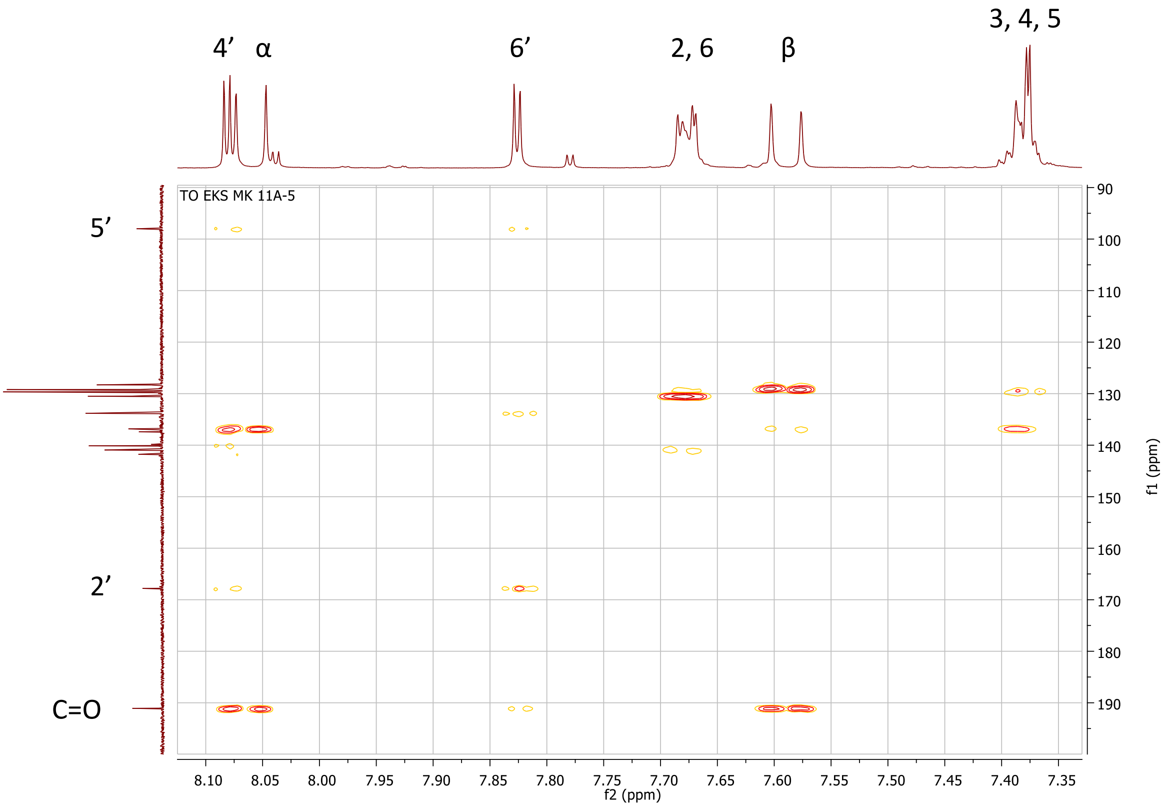

Figure S67. HMBC contour map – ^{1}H x ^{13}C expansion of 5′-bromo-2′-hydroxy-3′-nitrochalcone.

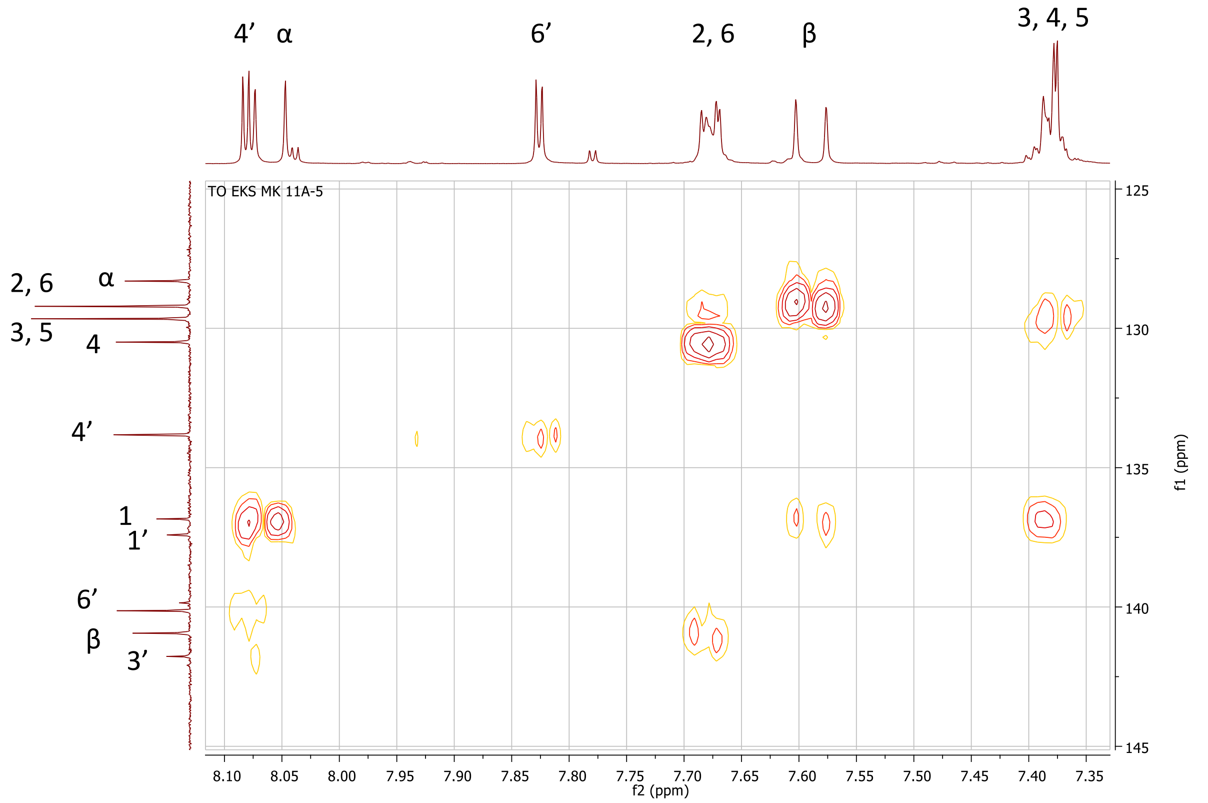

Figure S68. HMBC contour map – ^1H x ^{13}C expansion of 5′-bromo-2′-hydroxy-3′-nitrochalcone.

Compound name: 6-bromo-8-nitroflavanone
Molecular Formula: $C_{15}H_{10}BrNO_4$
Formula Weight: 348.148
Ionization mode: negative
Precursor $[M + H]^+$: 345.979
Monoisotopic Mass: 346.979
Collision energy (CE): +25.0

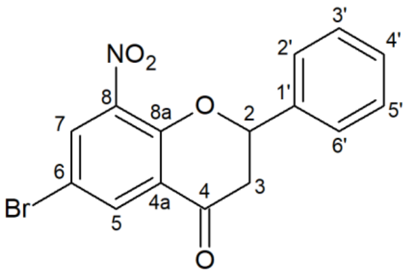

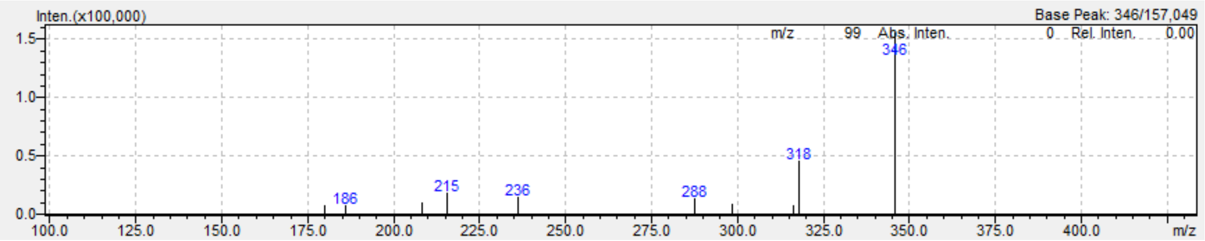

Figure S69. LC-MS spectrum of 6-bromo-8-nitroflavanone.

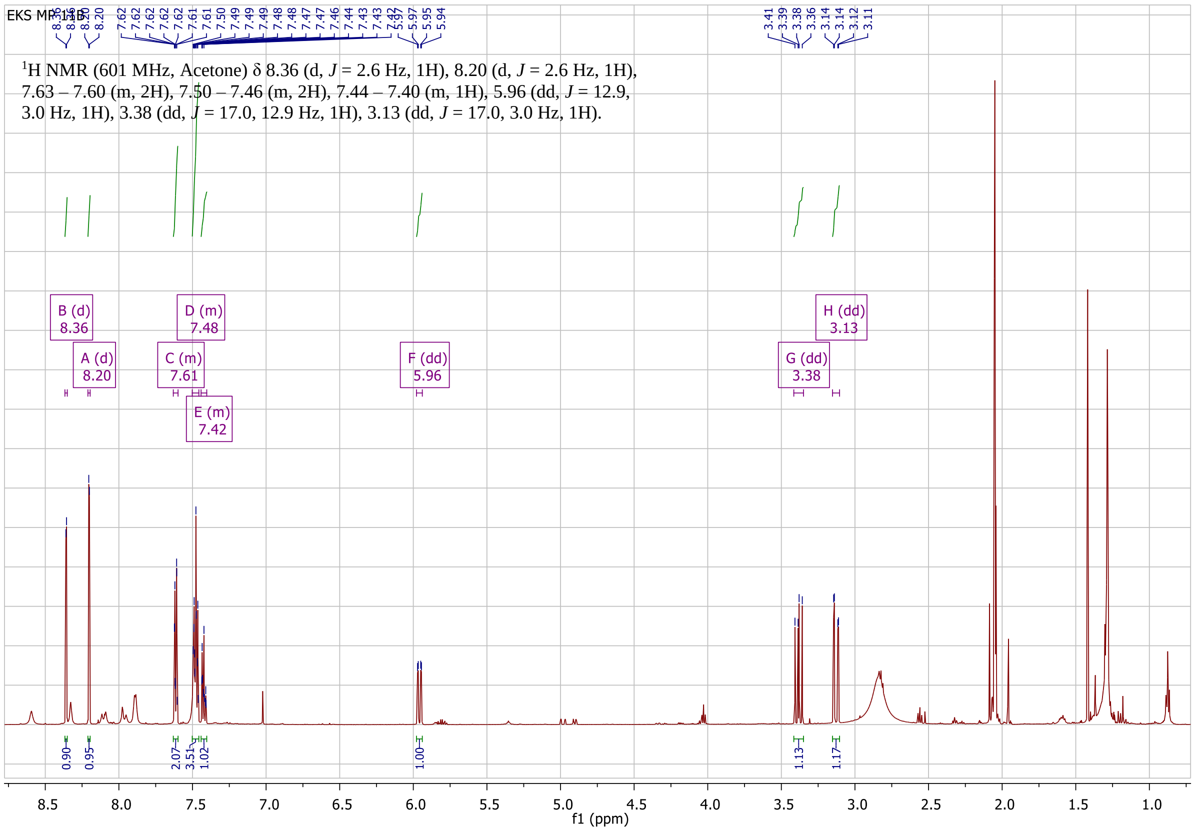

Figure S70. ¹H NMR spectrum of 6-bromo-8-nitroflavanone.

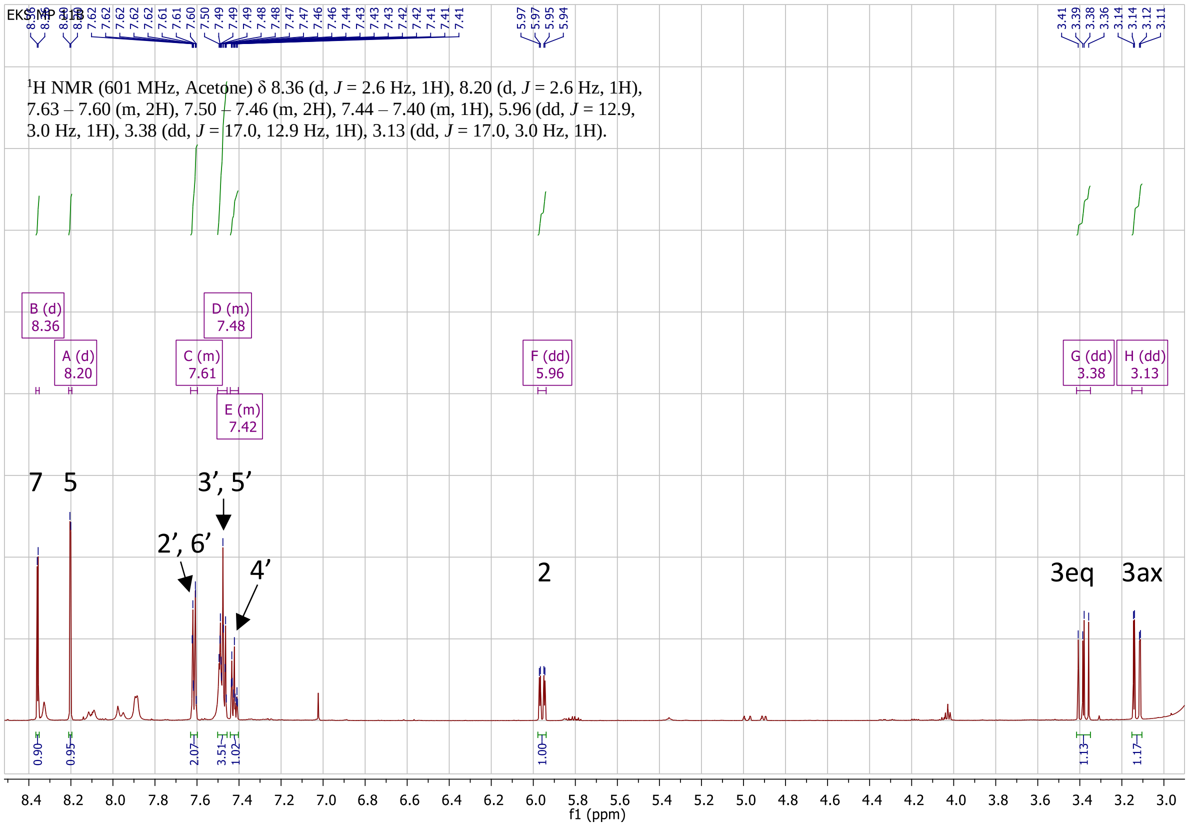

Figure S71. ¹H NMR spectrum expansion of 6-bromo-8-nitroflavanone.

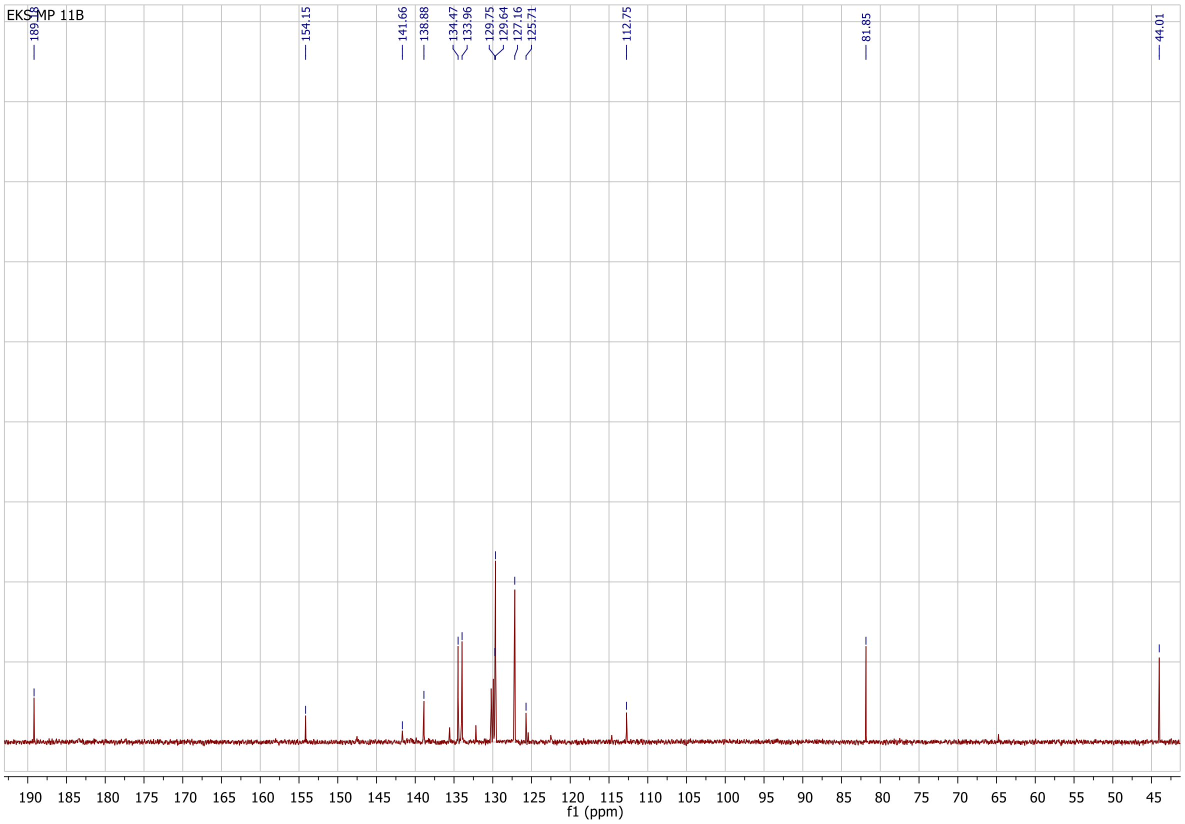

Figure S72. ^{31}C NMR spectrum of 6-bromo-8-nitroflavanone.

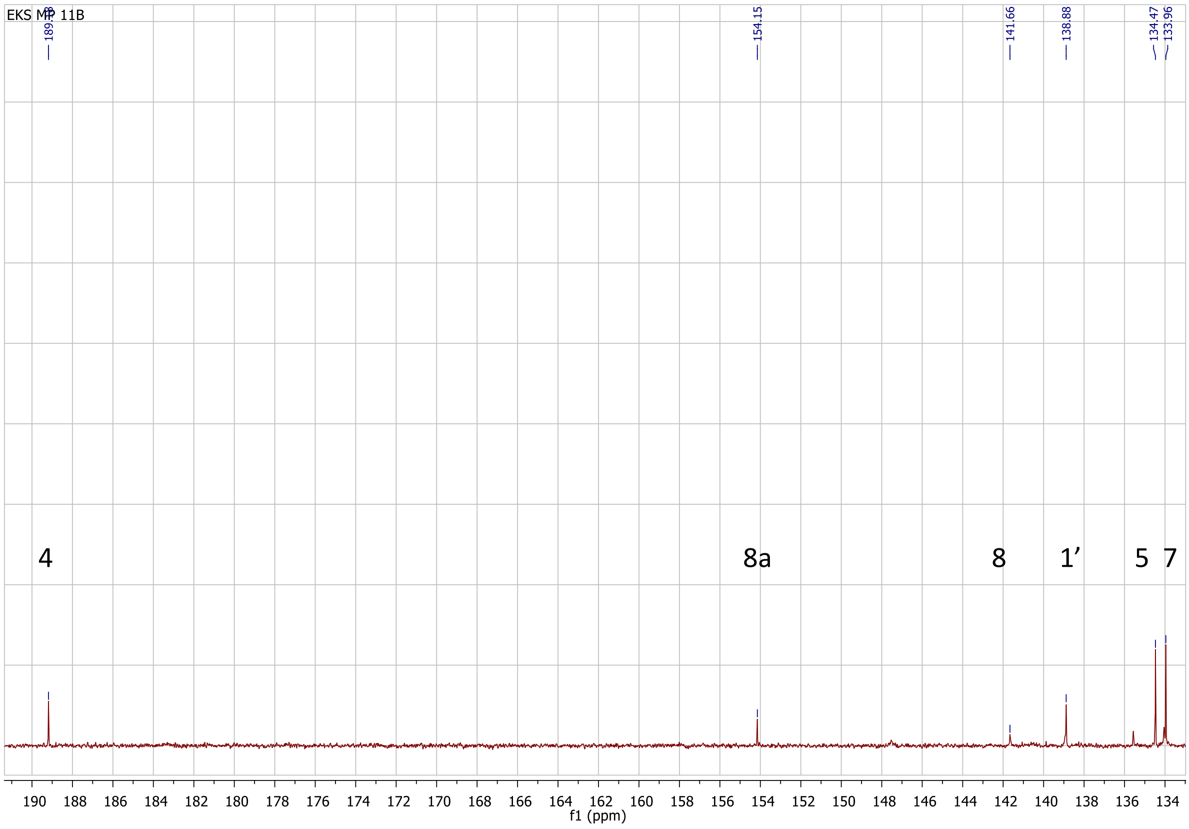

Figure S73. ^{31}C NMR spectrum expansion of 6-bromo-8-nitroflavanone.

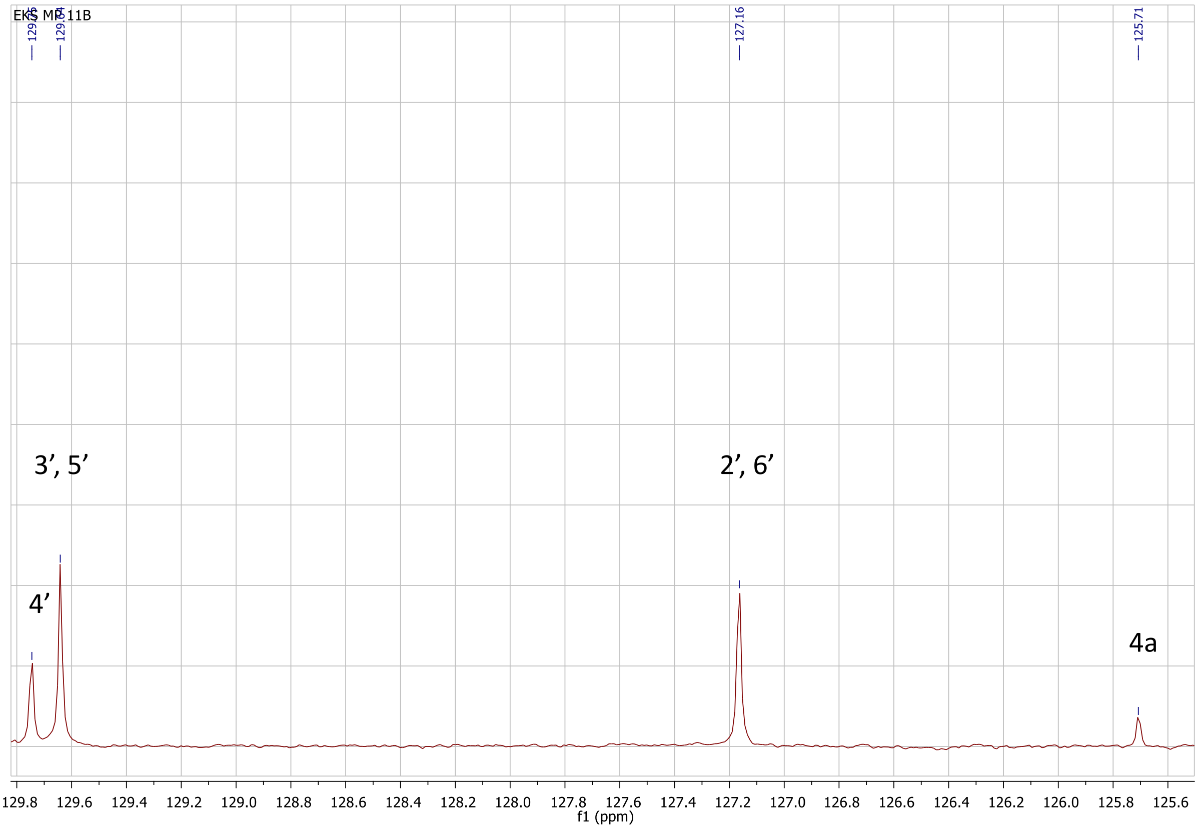

Figure S74. ^{31}C NMR spectrum expansion of 6-bromo-8-nitroflavanone.

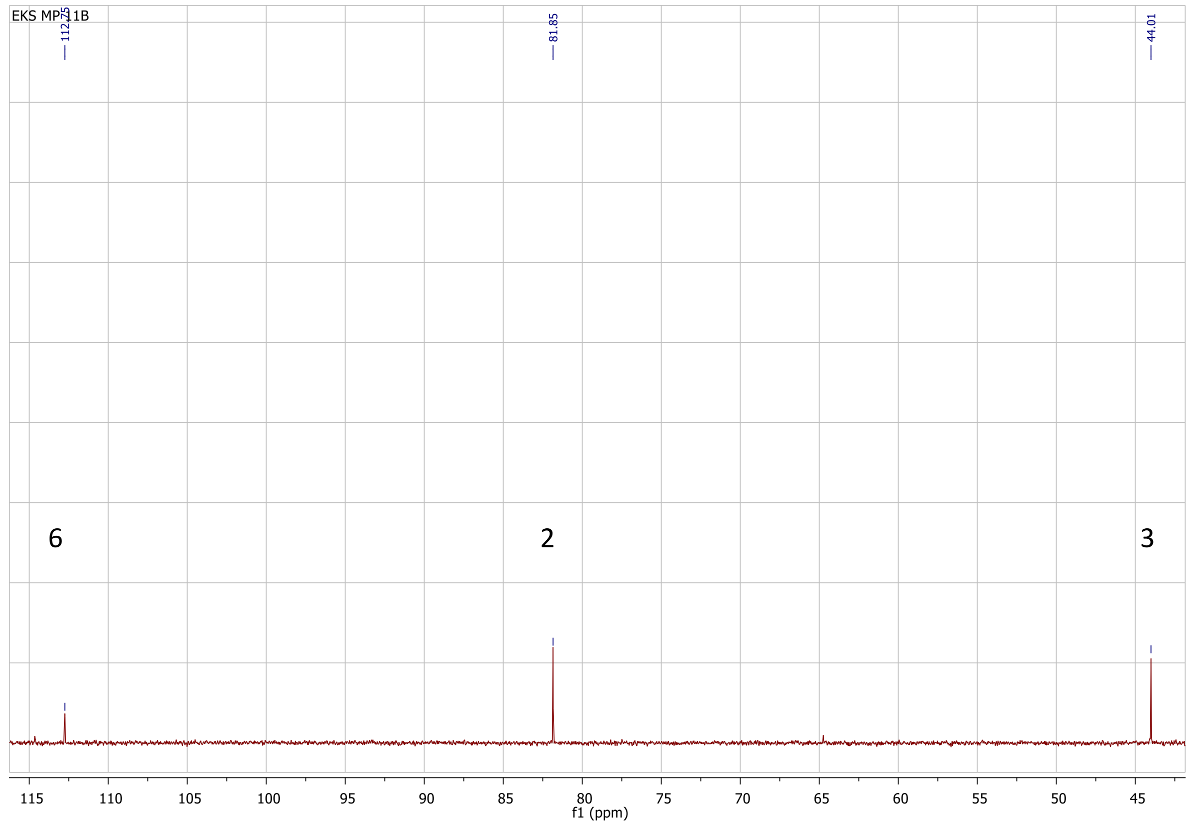

Figure S75. ^{31}C NMR spectrum expansion of 6-bromo-8-nitroflavanone.

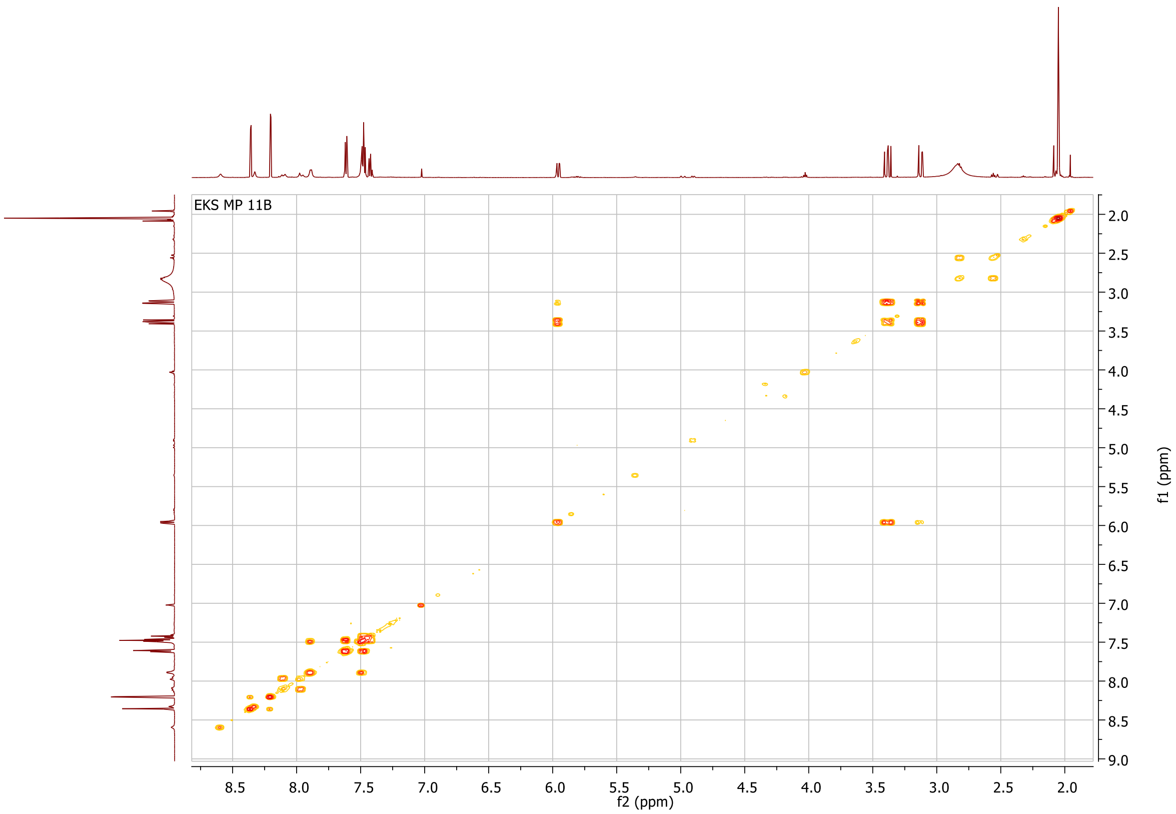

Figure S76. COSY contour map – ^{1}H x ^{1}H of 6-bromo-8-nitroflavanone.

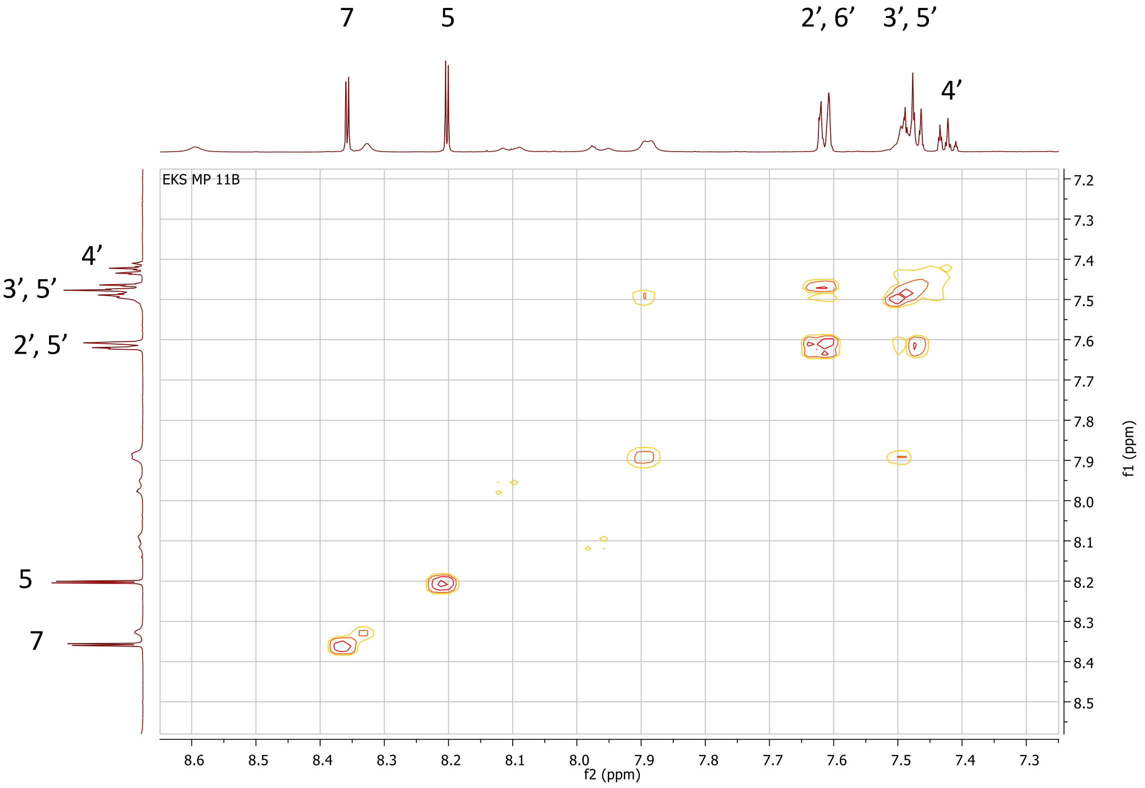

Figure S77. COSY contour map – ^{1}H x ^{1}H expansion of 6-bromo-8-nitroflavanone.

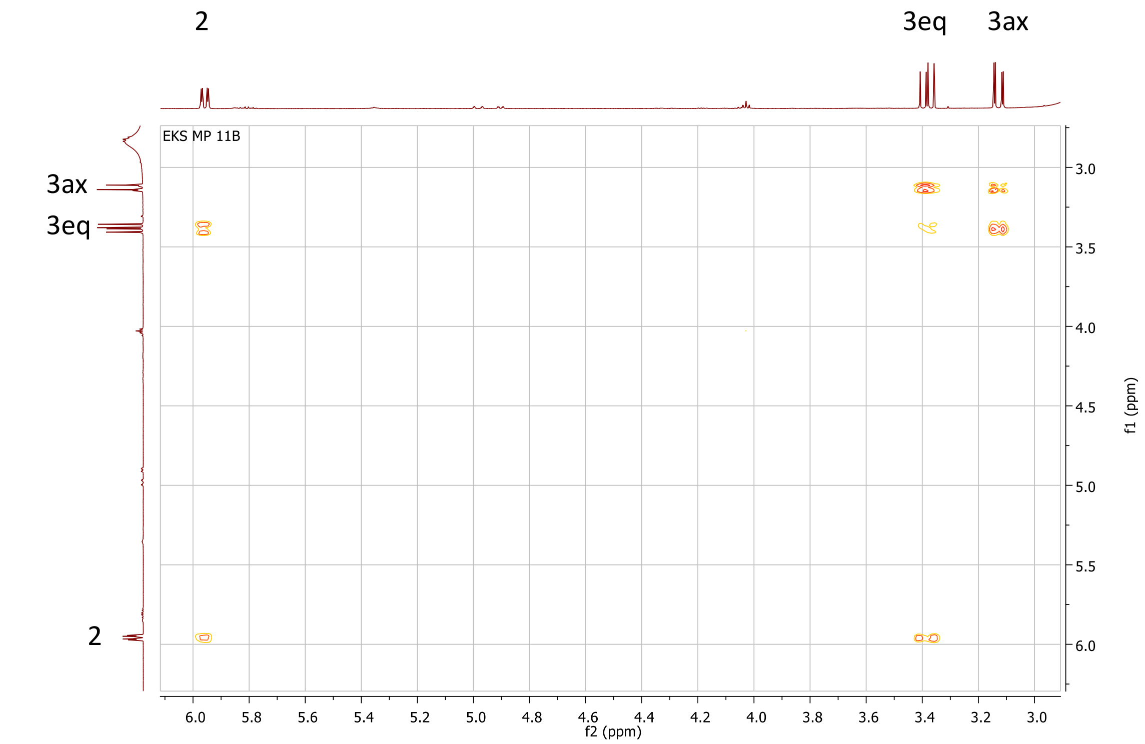

Figure S78. COSY contour map – ^1H x ^1H expansion of 6-bromo-8-nitroflavanone.

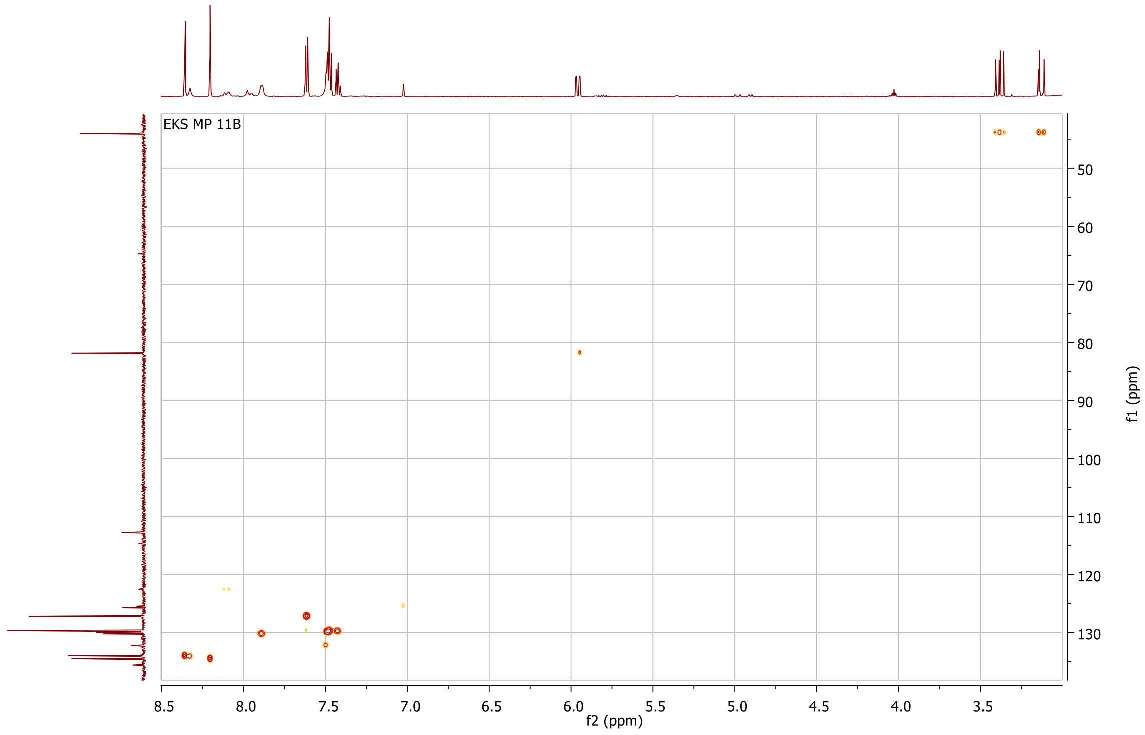

Figure S79. HSQC contour map – ^1H x ^{13}C of 6-bromo-8-nitroflavanone.

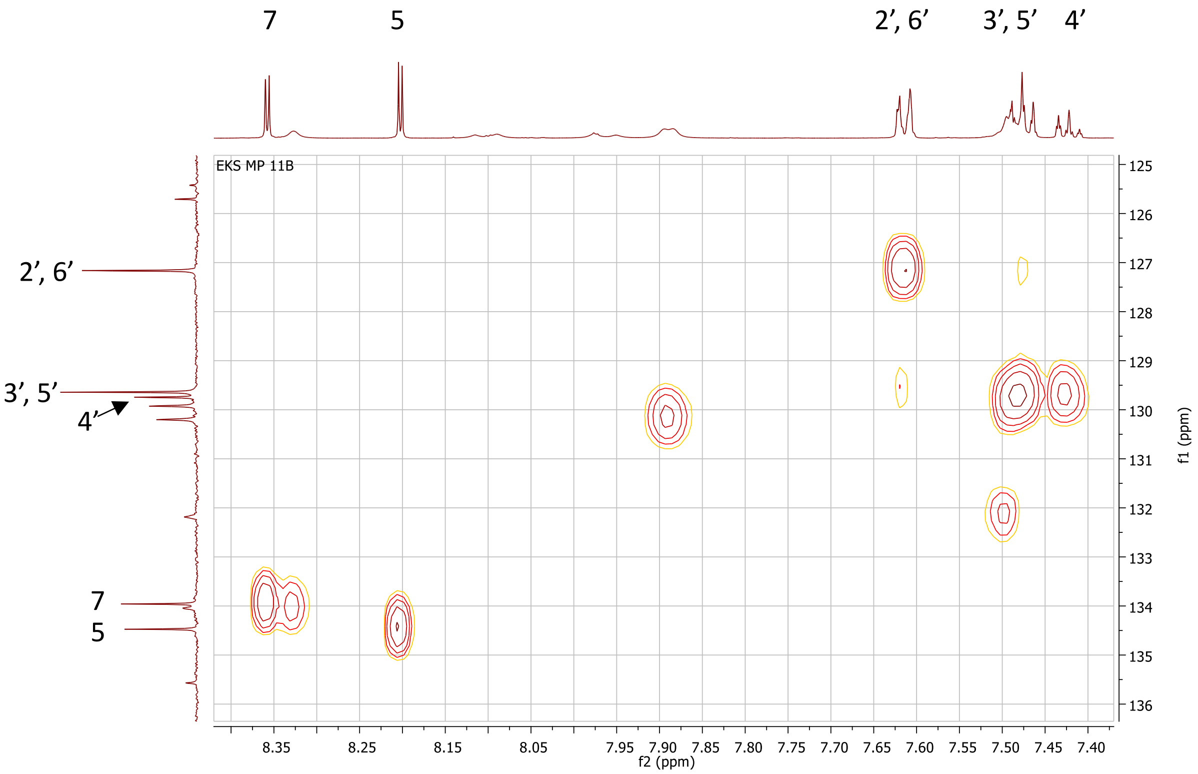

Figure S80. HSQC contour map – ^{1}H x ^{13}C expansion of 6-bromo-8-nitroflavanone.

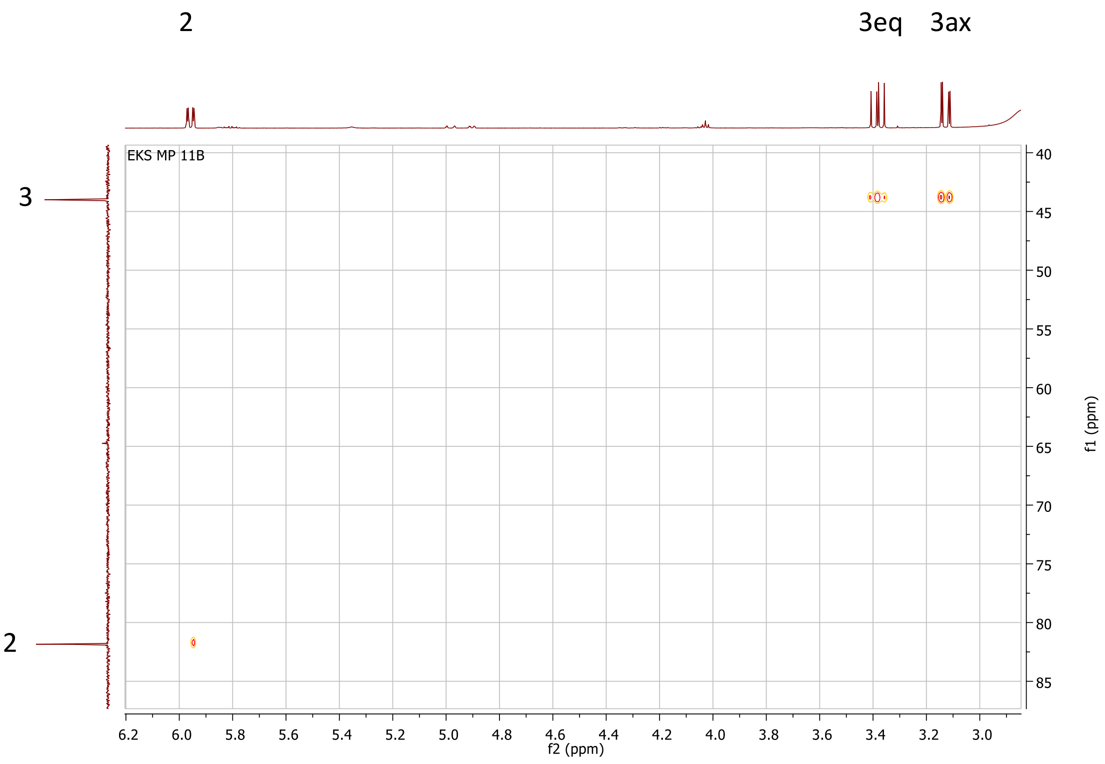

Figure S81. HSQC contour map – ^{1}H x ^{13}C expansion of 6-bromo-8-nitroflavanone.

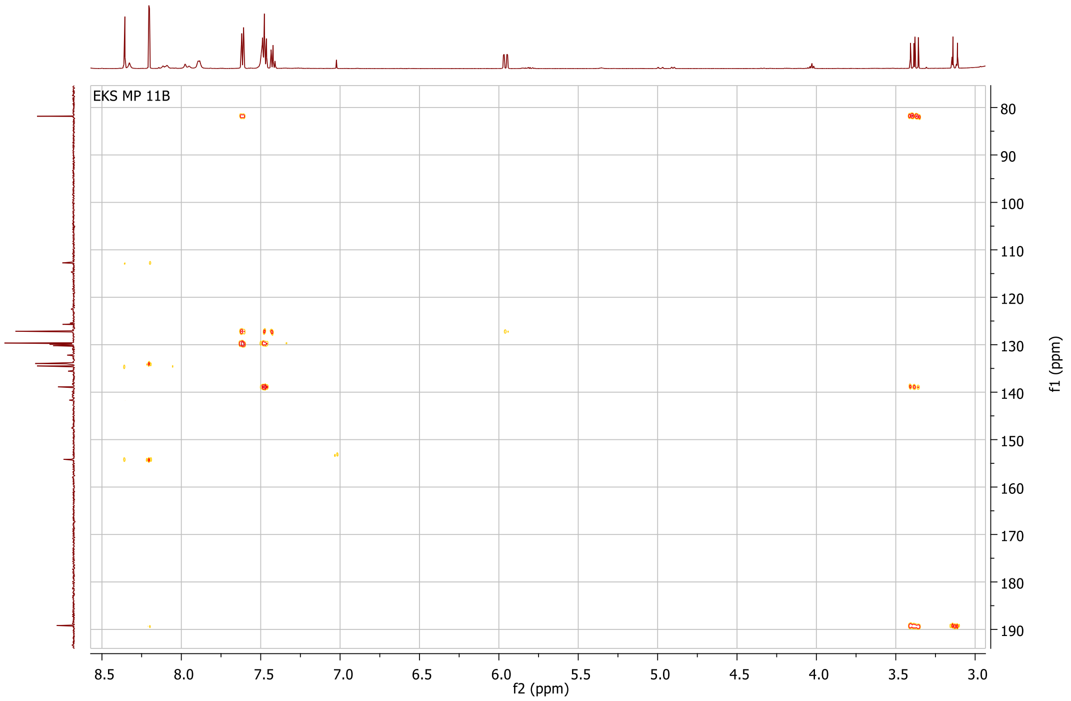

Figure S82. HMBC contour map – ¹H x ¹³C of 6-bromo-8-nitroflavanone.

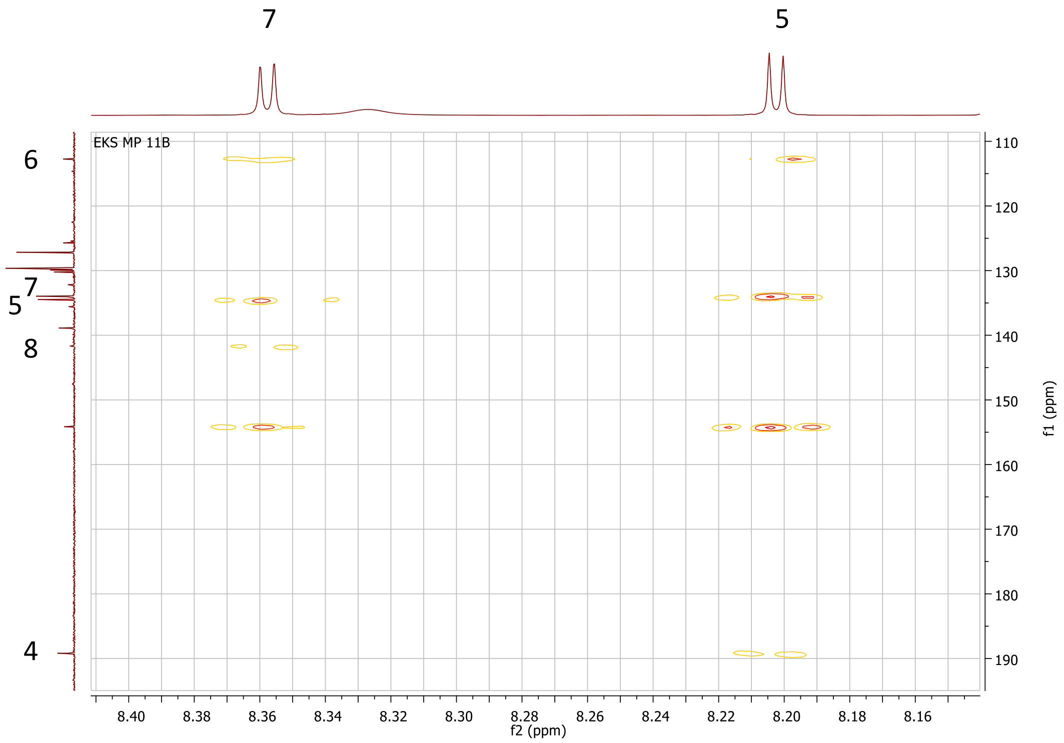

Figure S83. HMBC contour map – ¹H x ¹³C expansion of 6-bromo-8-nitroflavanone.

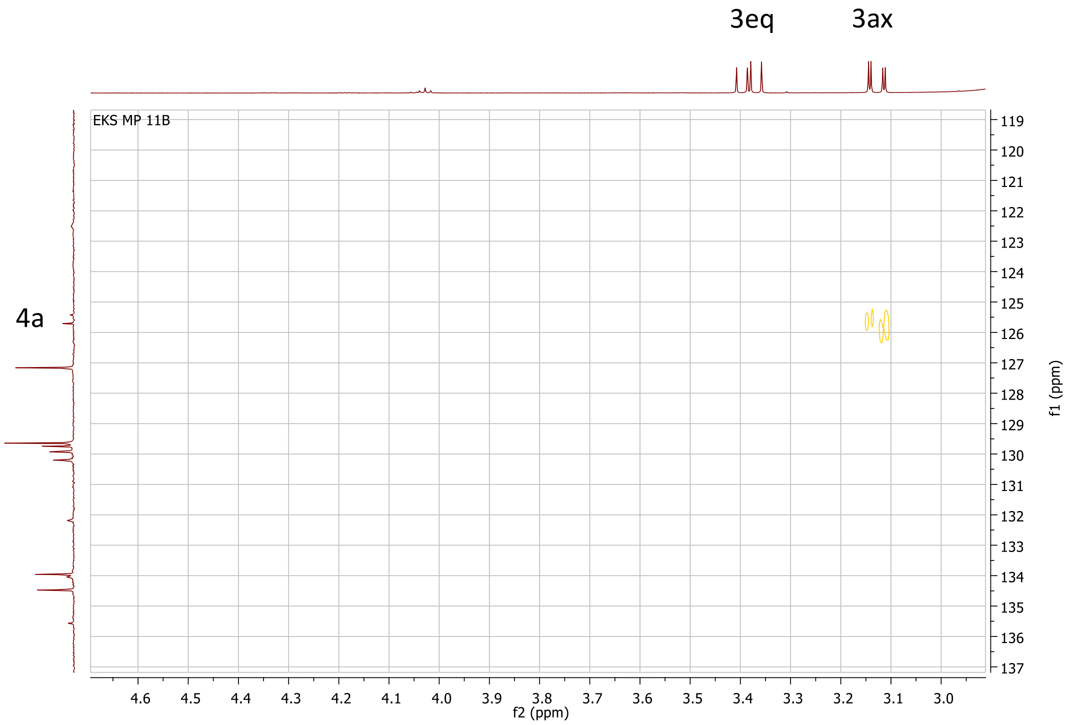

Figure S84. HMBC contour map – ^{1}H x ^{13}C expansion of 6-bromo-8-nitroflavanone.

Compound name: 6-bromo-8-nitroflavone
Molecular Formula: $C_{15}H_8BrNO_4$
Formula Weight: 346.132
Ionization mode: positive
Precursor $[M + H]^+$: 345.964
Monoisotopic Mass: 344.964
Collision energy (CE): -25.0

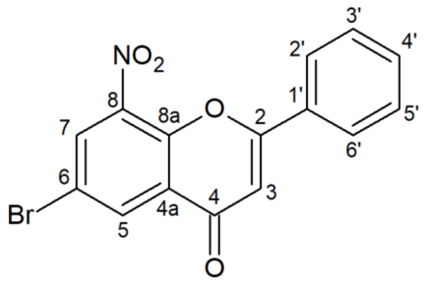

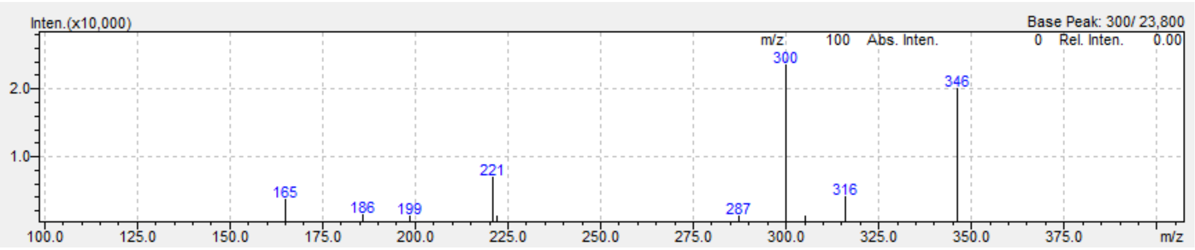

Figure S85. LC-MS spectrum of 6-bromo-8-nitroflavone.

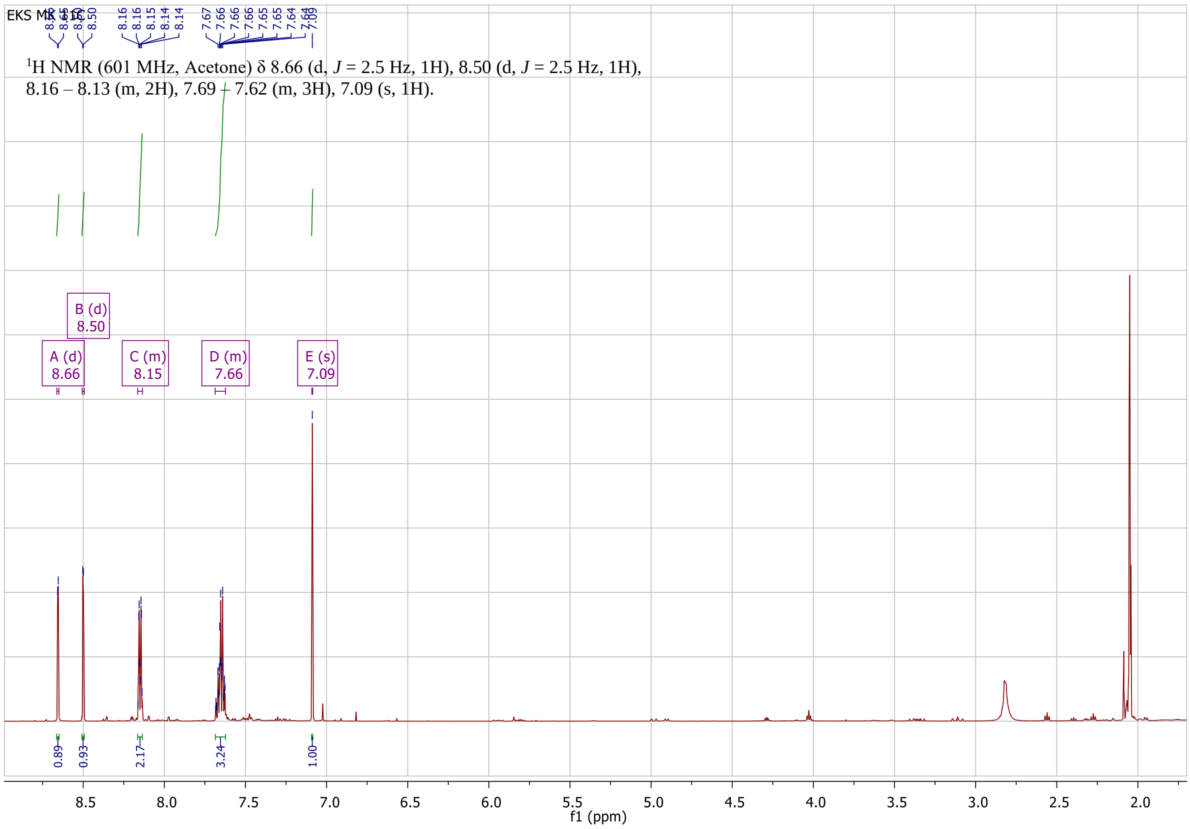

Figure S86. ¹H NMR spectrum of 6-bromo-8-nitroflavone.

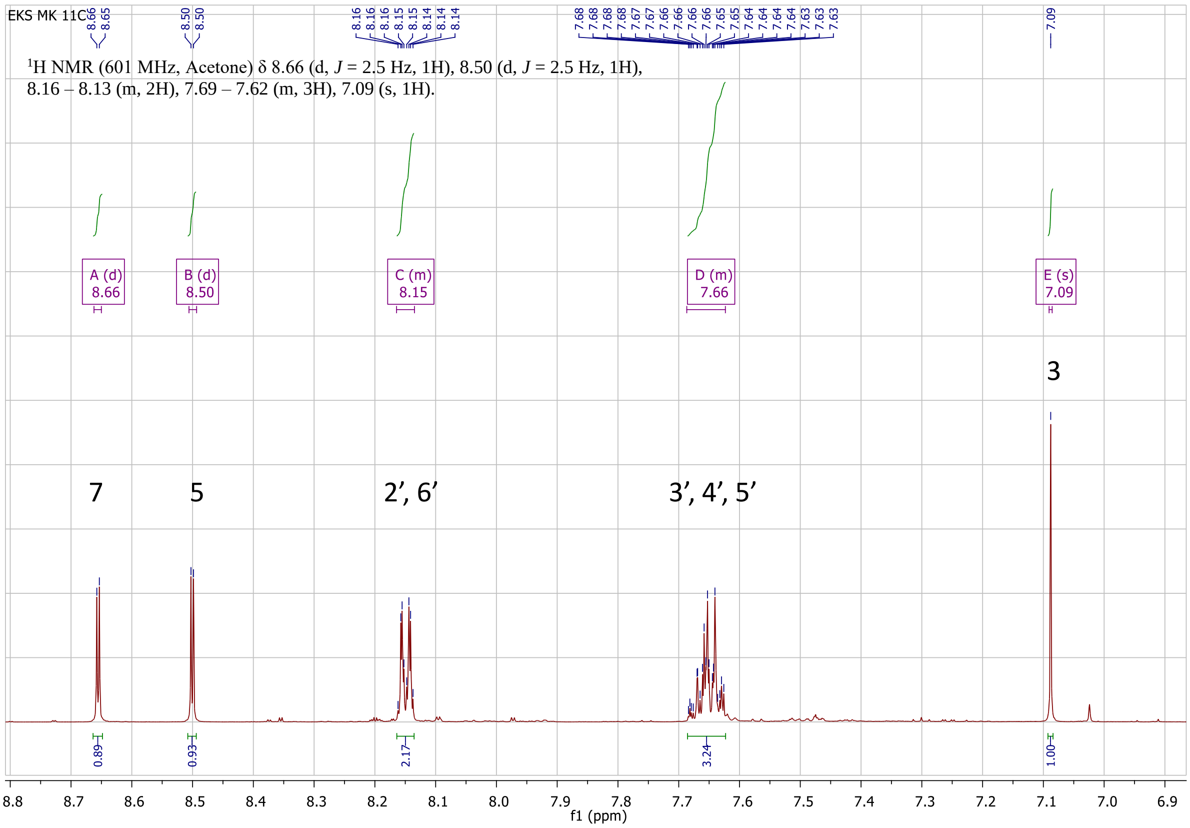

Figure S87. ¹H NMR spectrum expansion of 6-bromo-8-nitroflavone.

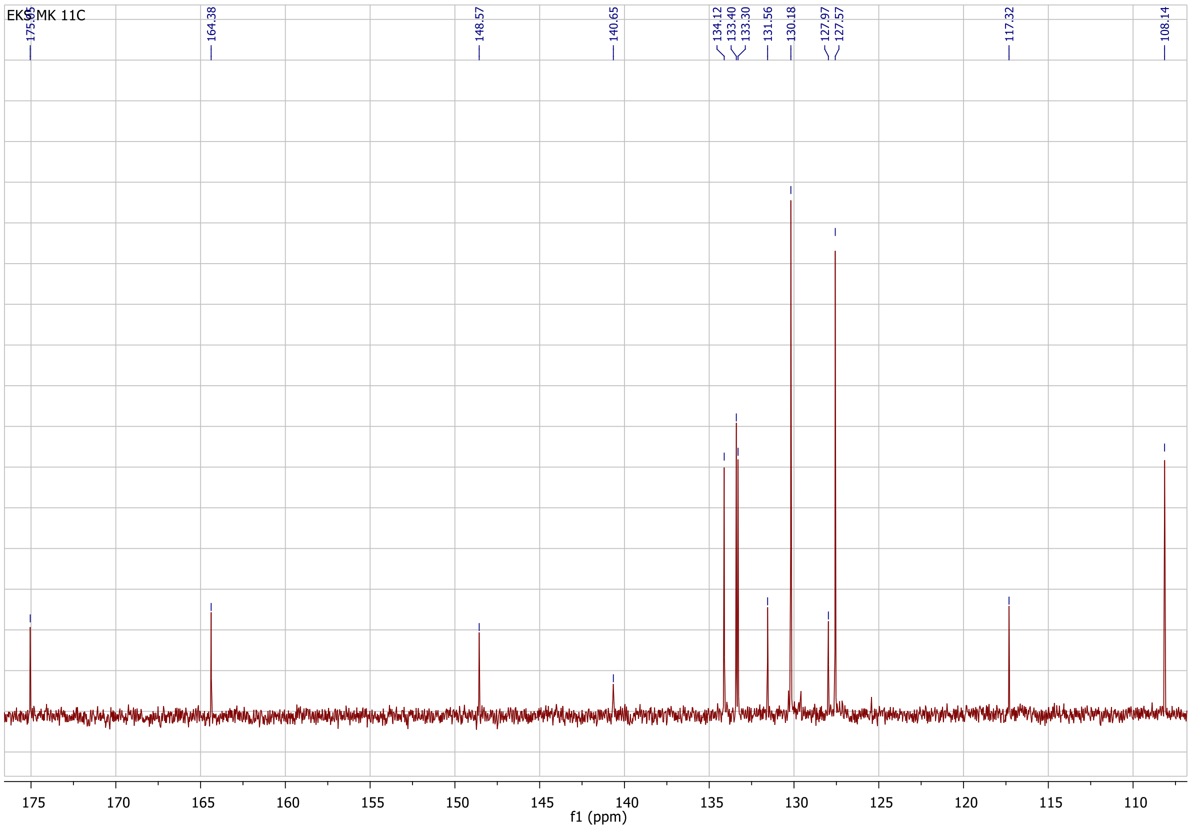

Figure S88. ^{13}C NMR spectrum of 6-bromo-8-nitroflavone.

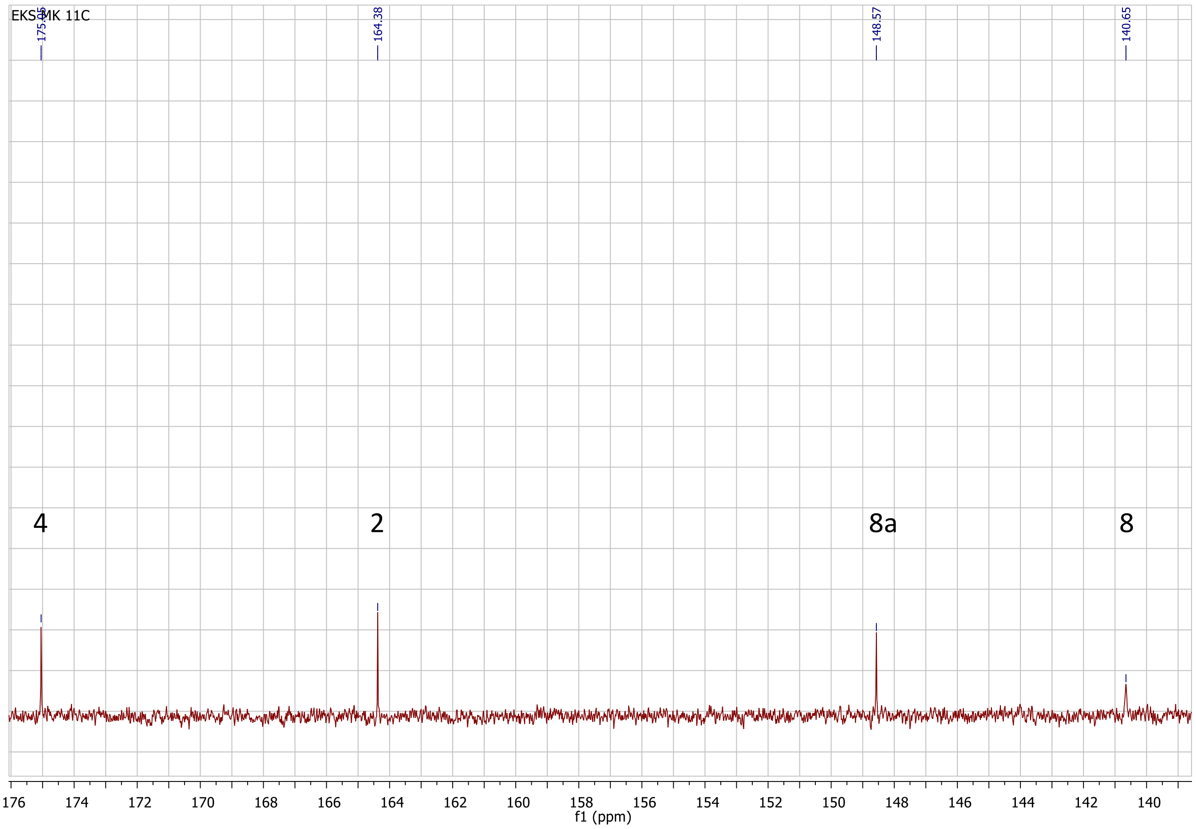

Figure S89. ^{13}C NMR spectrum expansion of 6-bromo-8-nitroflavone.

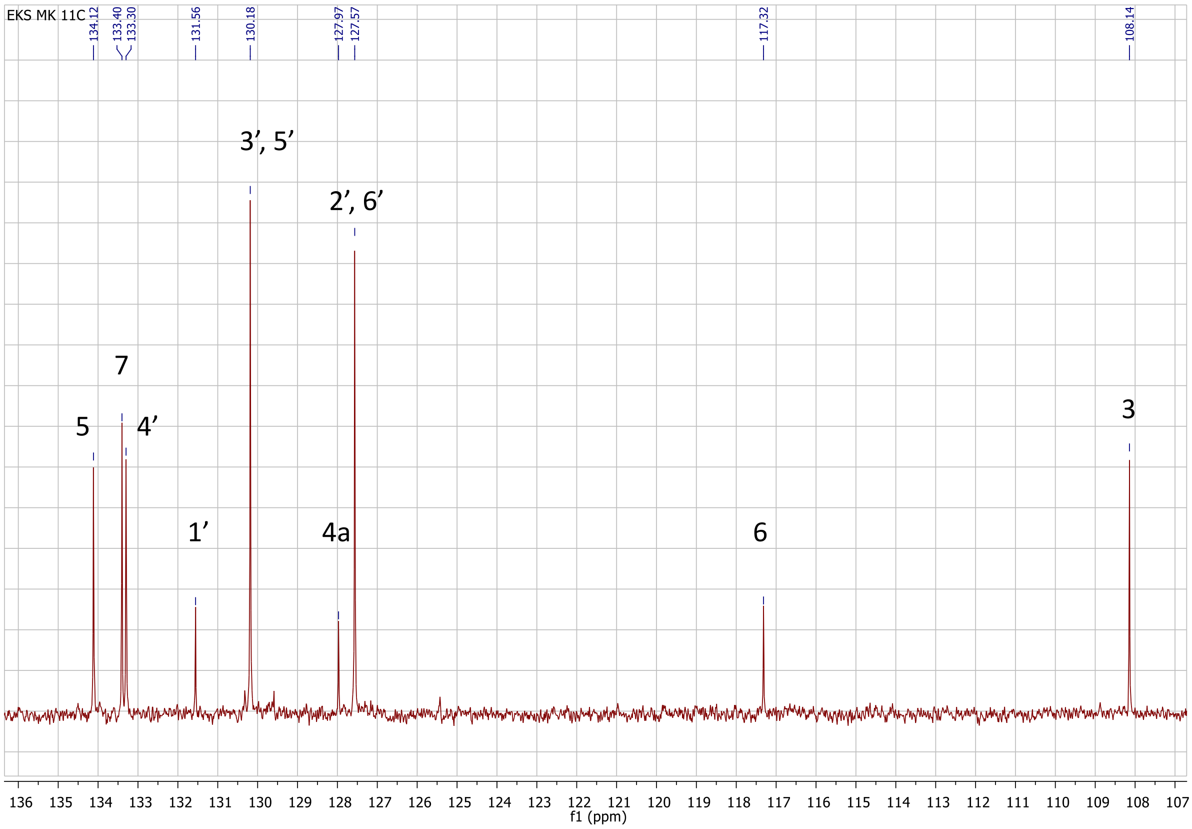

Figure S90. ^{13}C NMR spectrum expansion of 6-bromo-8-nitroflavone.

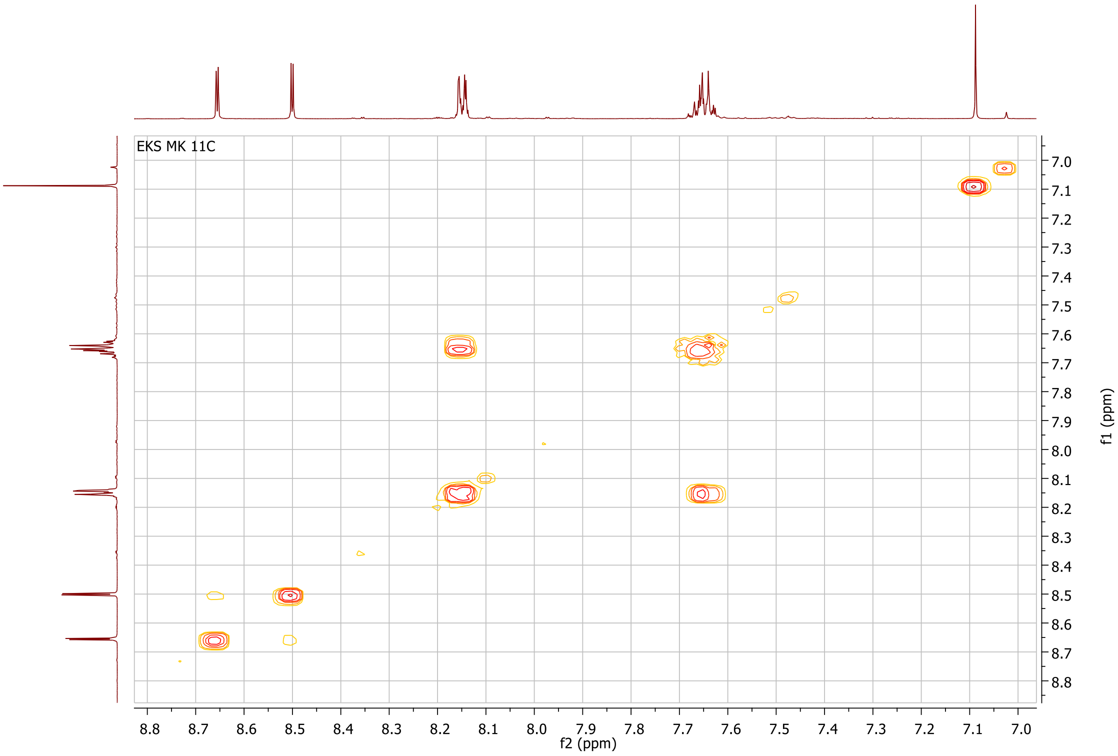

Figure S91. COSY contour map – ^{1}H x ^{1}H of 6-bromo-8-nitroflavone.

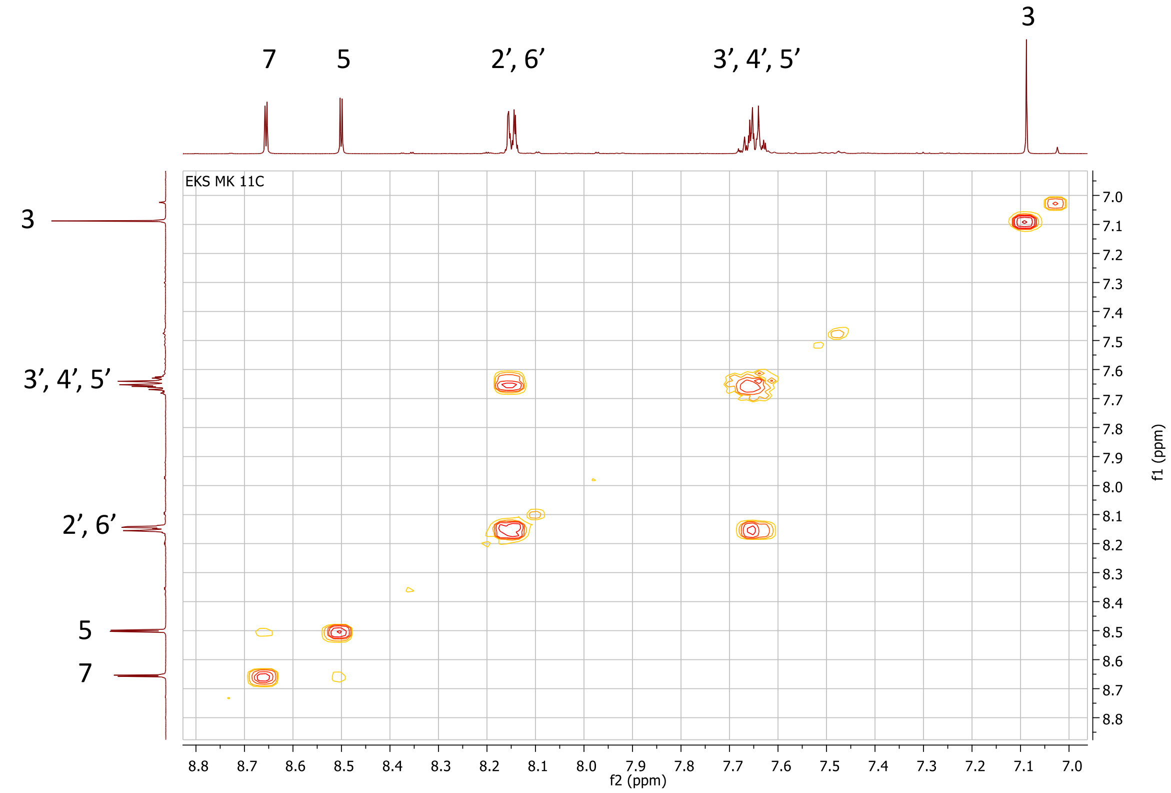

Figure S92. COSY contour map – ^1H x ^1H expansion of 6-bromo-8-nitroflavone.

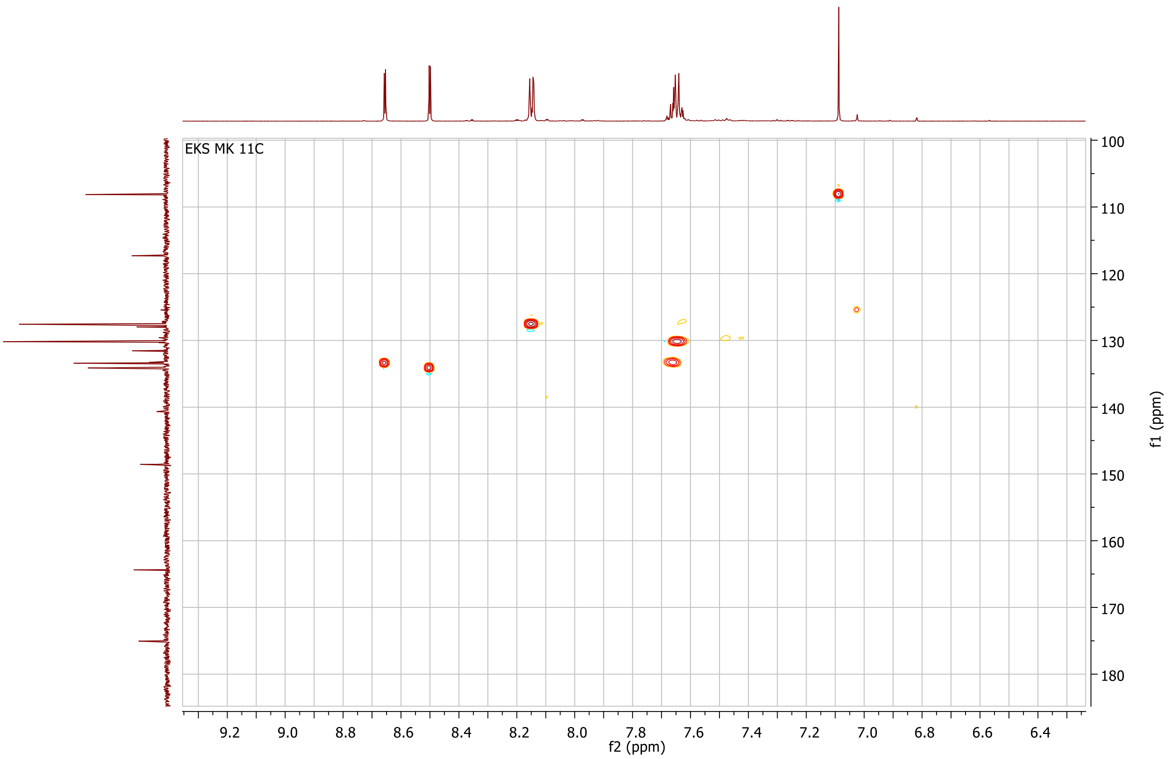

Figure S93. HSQC contour map – ^1H x ^{13}C of 6-bromo-8-nitroflavone.

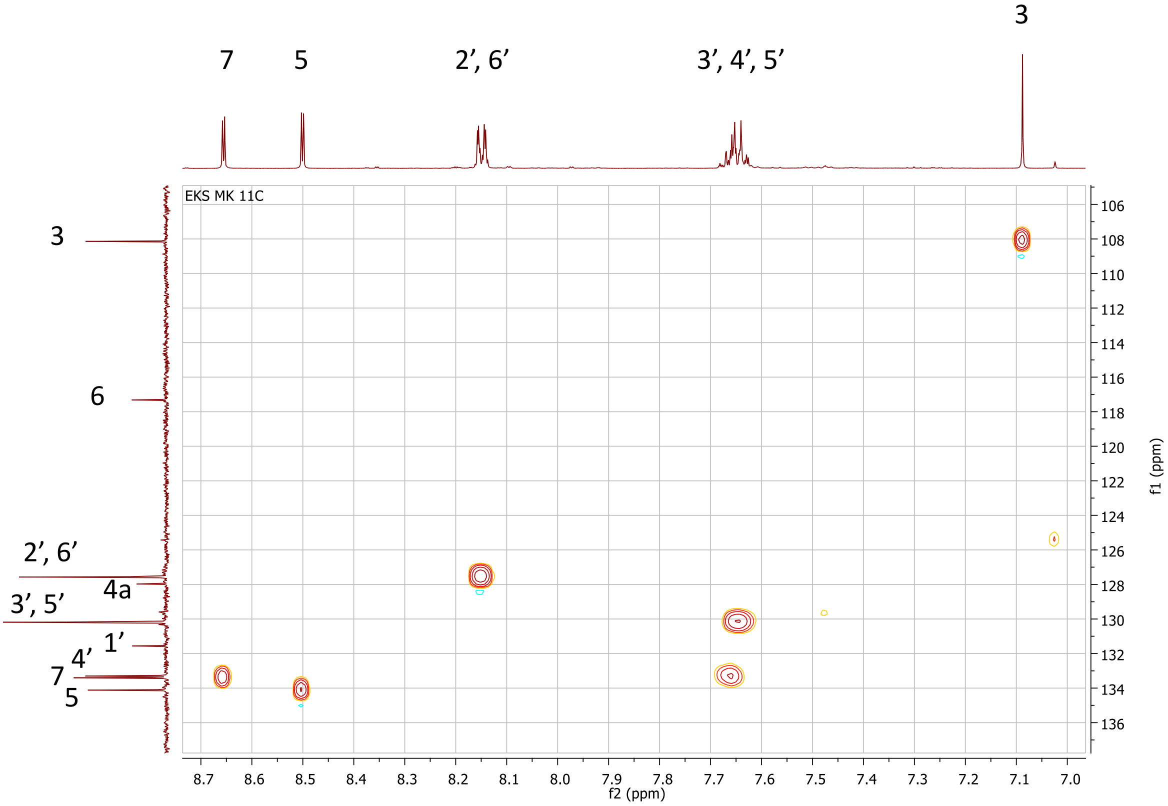

Figure S94. HSQC contour map – ¹H x ¹³C expansion of 6-bromo-8-nitroflavone.

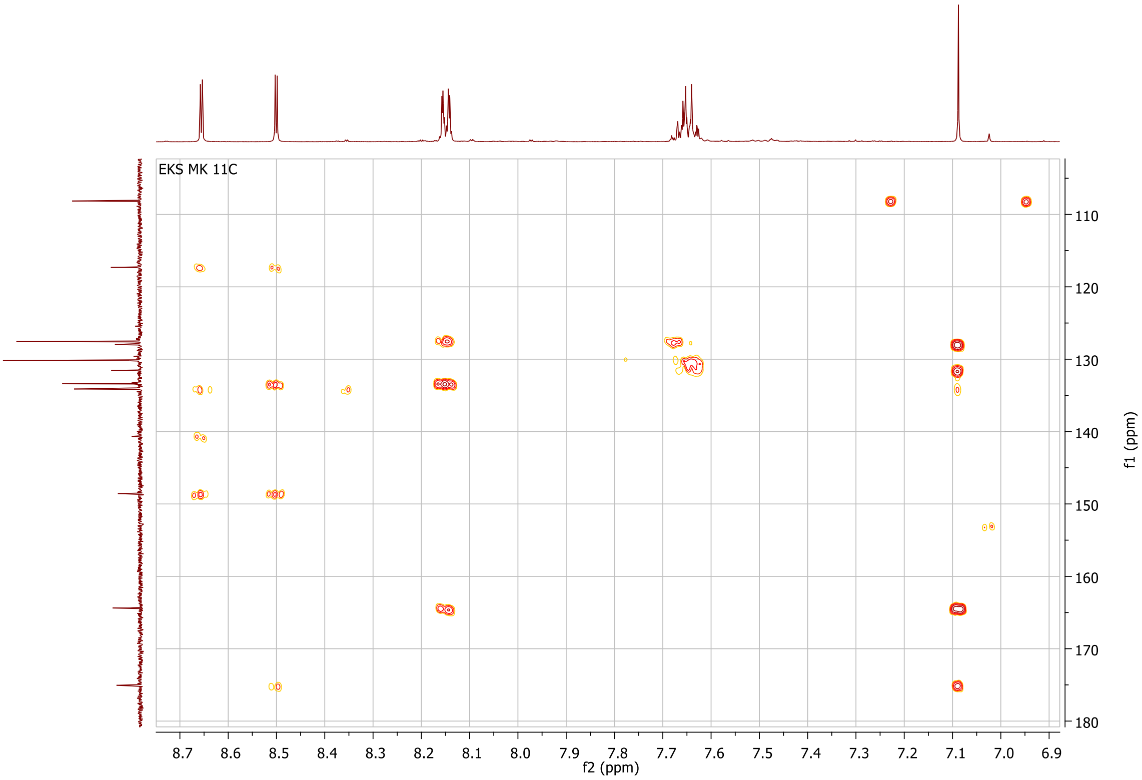

Figure S95. HMBC contour map – ¹H x ¹³C of 6-bromo-8-nitroflavone.

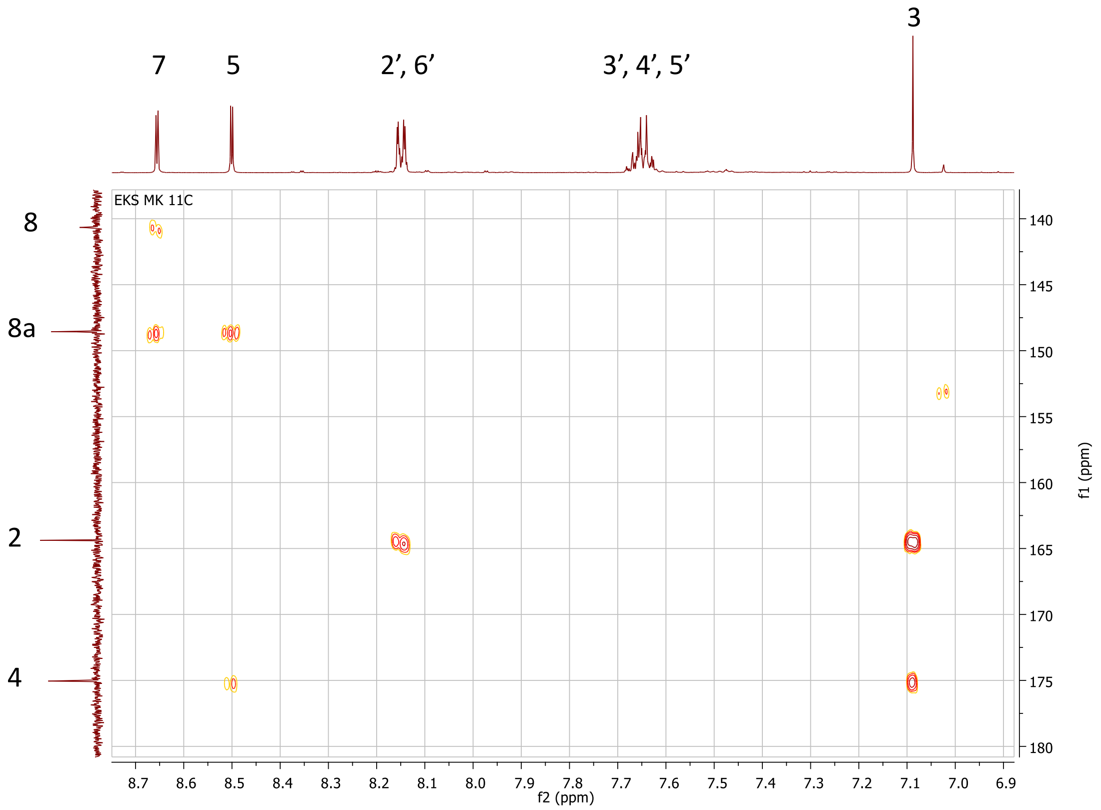

Figure S96. HMBC contour map – ^{1}H x ^{13}C expansion of 6-bromo-8-nitroflavone.

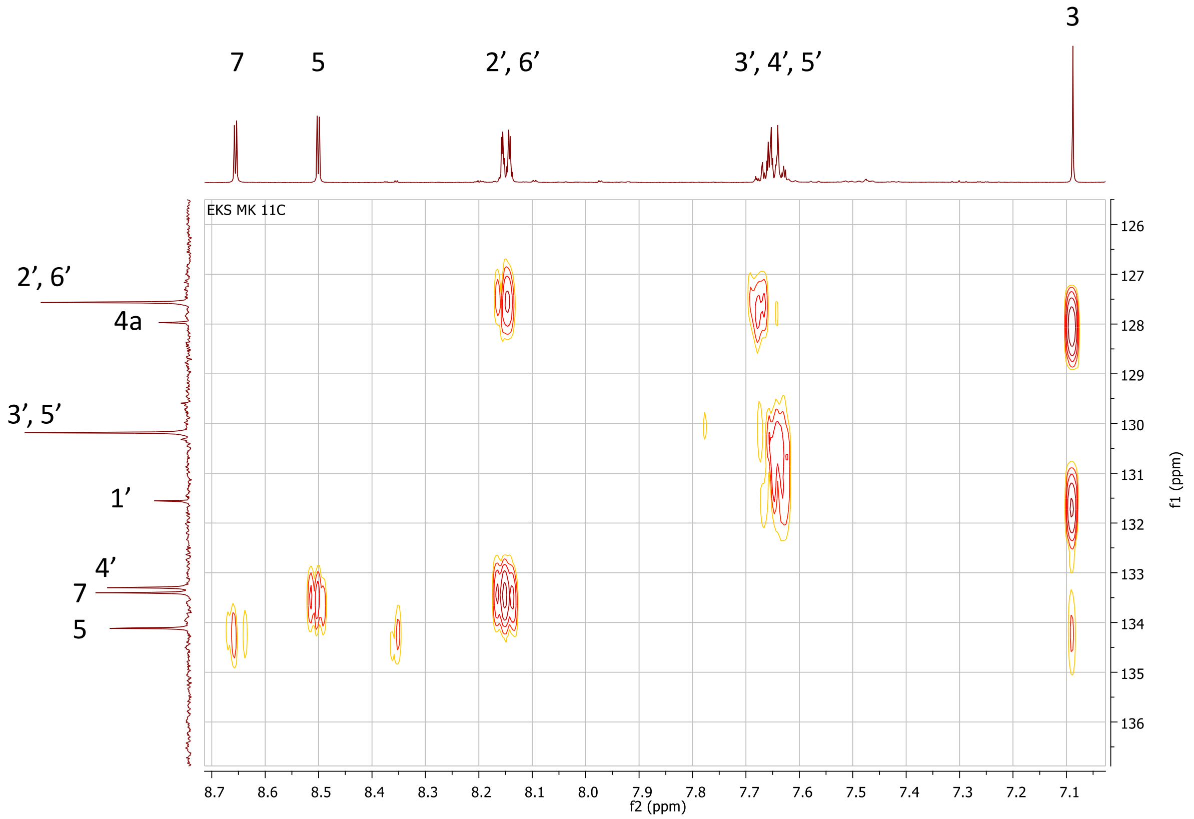

Figure S97. HMBC contour map – ^{1}H x ^{13}C expansion of 6-bromo-8-nitroflavone.

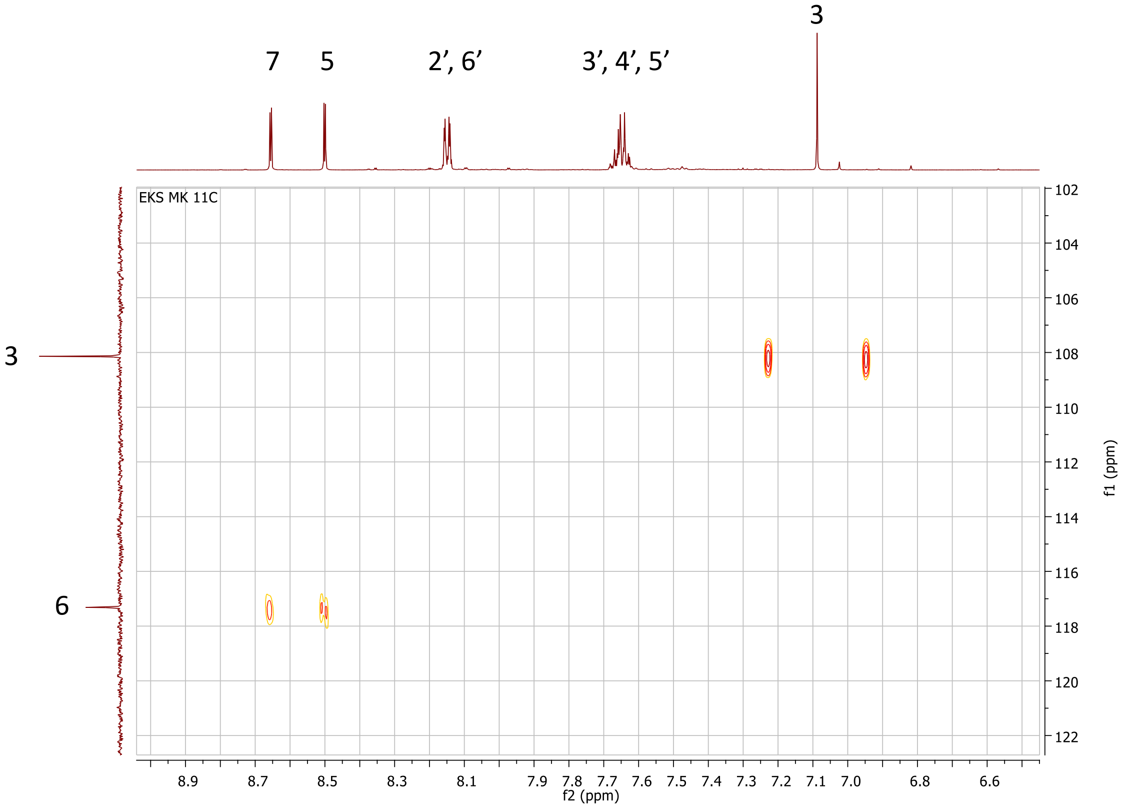

Figure S98. HMBC contour map – ^{1}H x ^{13}C expansion of 6-bromo-8-nitroflavone.

Compound name: 6-Bromo-8-nitroflavone 4′-*O*-*β*-D-(4″-O-methyl)-glucopyranoside
Molecular Formula: $C_{22}H_{20}BrNO_{10}$
Formula Weight: 538.299
Ionization mode: positive
Precursor $[M + H]^+$: 539.030
Monoisotopic Mass: 537.027
Collision energy (CE): -25.0

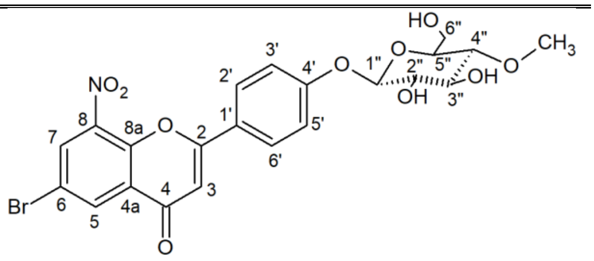

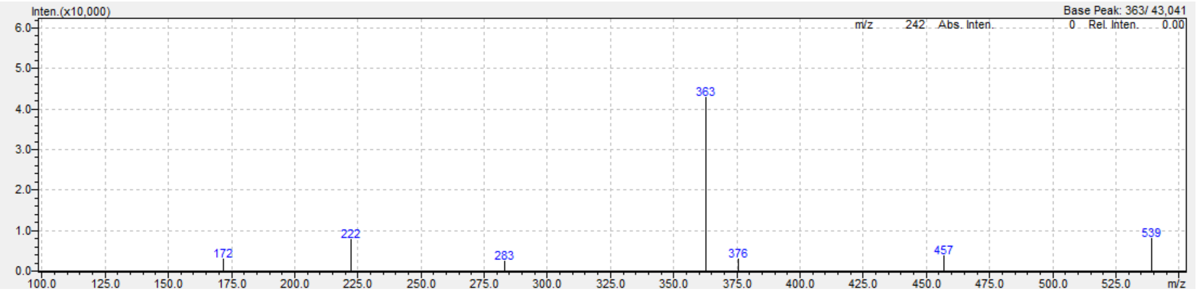

Figure S99. LC-MS spectrum of 6-bromo-8-nitroflavone 4′-O-β-D-(4″-O-methyl)-glucopyranoside.

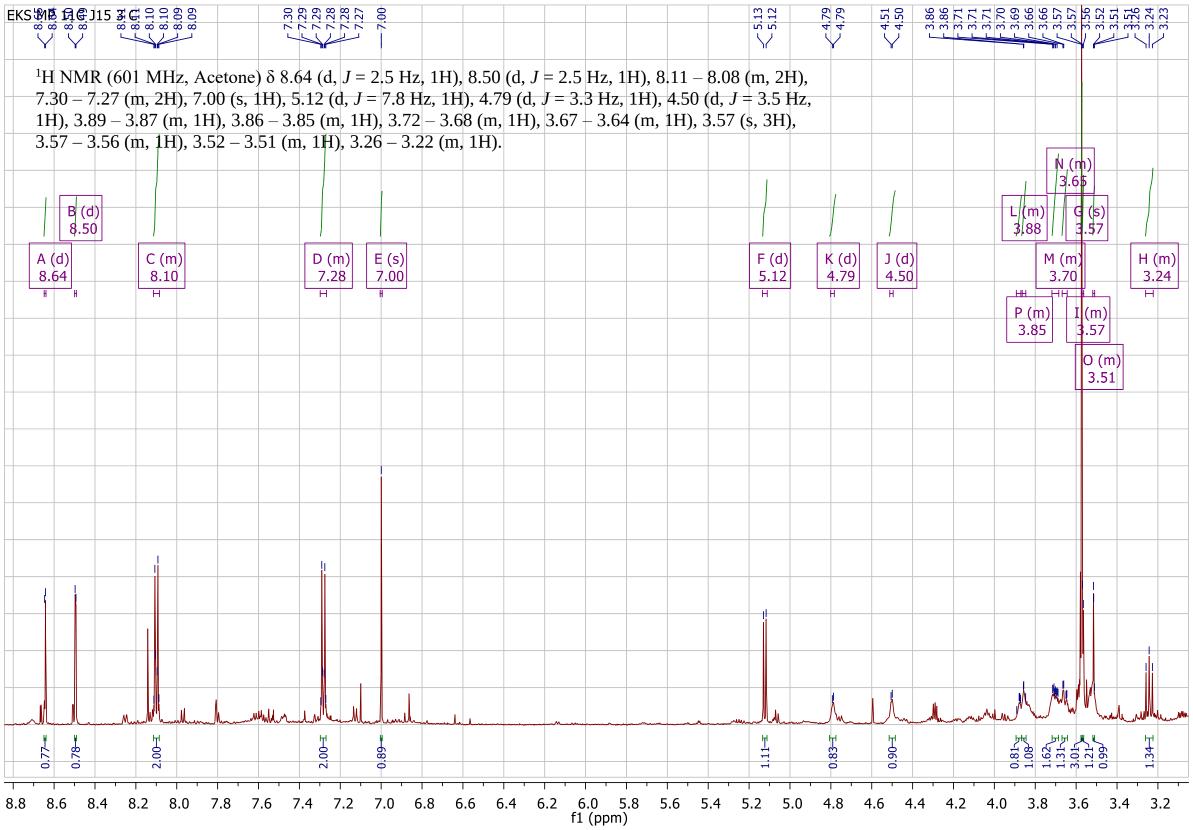

Figure S100. ¹H NMR spectrum of 6-bromo-8-nitroflavone 4′-O-β-D-(4″-O-methyl)-glucopyranoside.

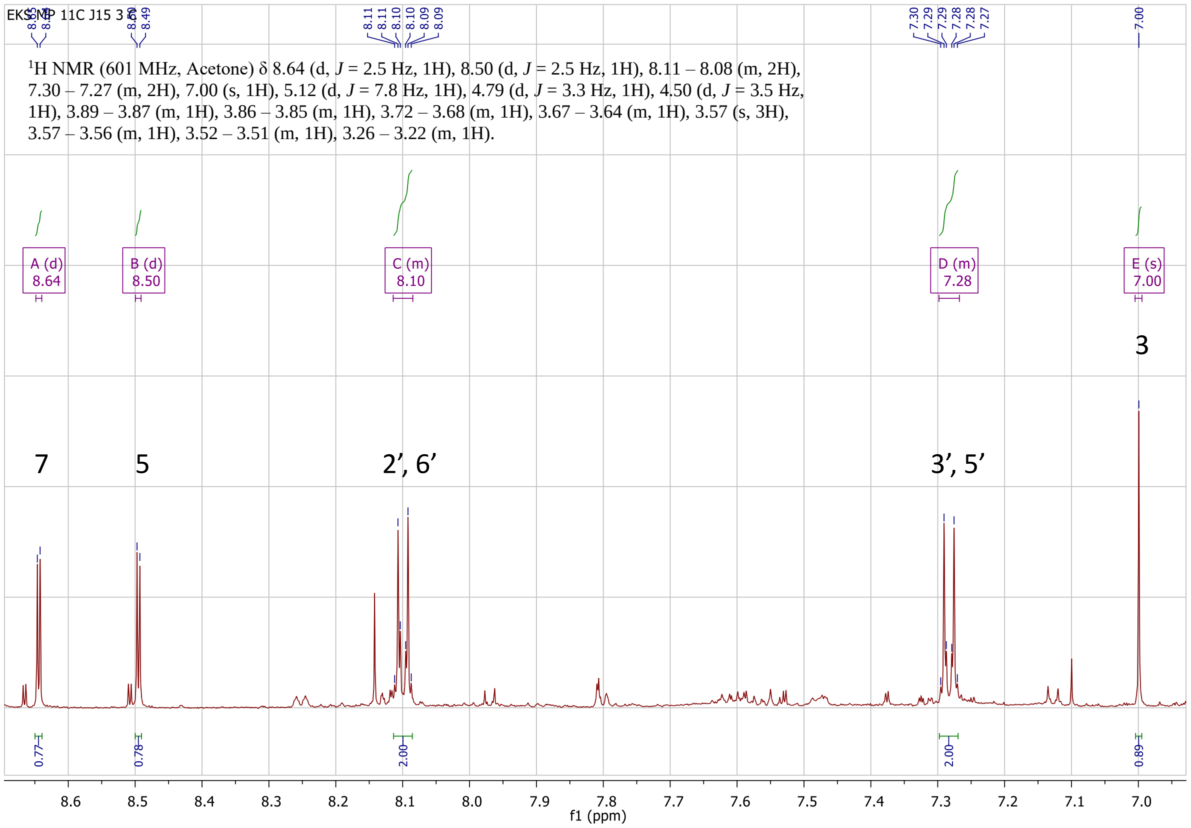

Figure S101. ¹H NMR spectrum expansion of 6-bromo-8-nitroflavone 4′-O-β-D-(4″-O-methyl)-glucopyranoside.

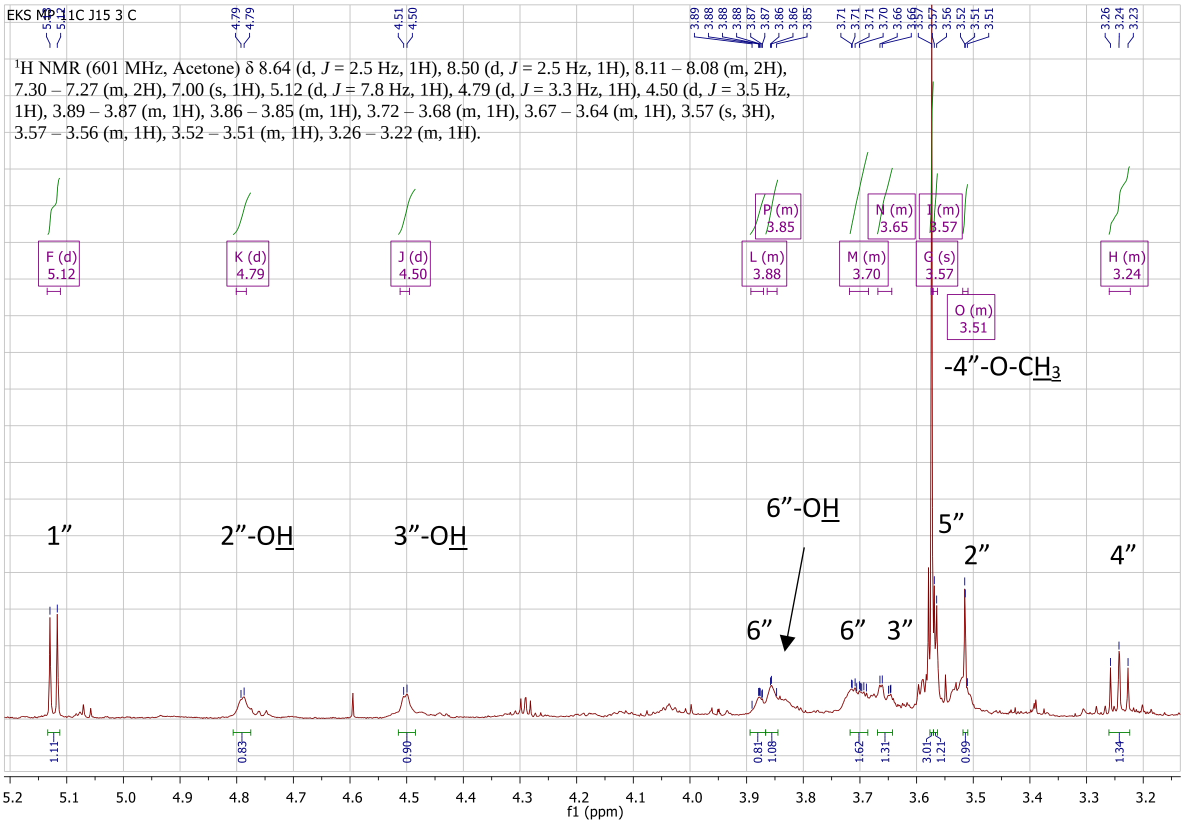

Figure S102. ¹H NMR spectrum expansion of 6-bromo-8-nitroflavone 4′-O-β-D-(4″-O-methyl)-glucopyranoside.

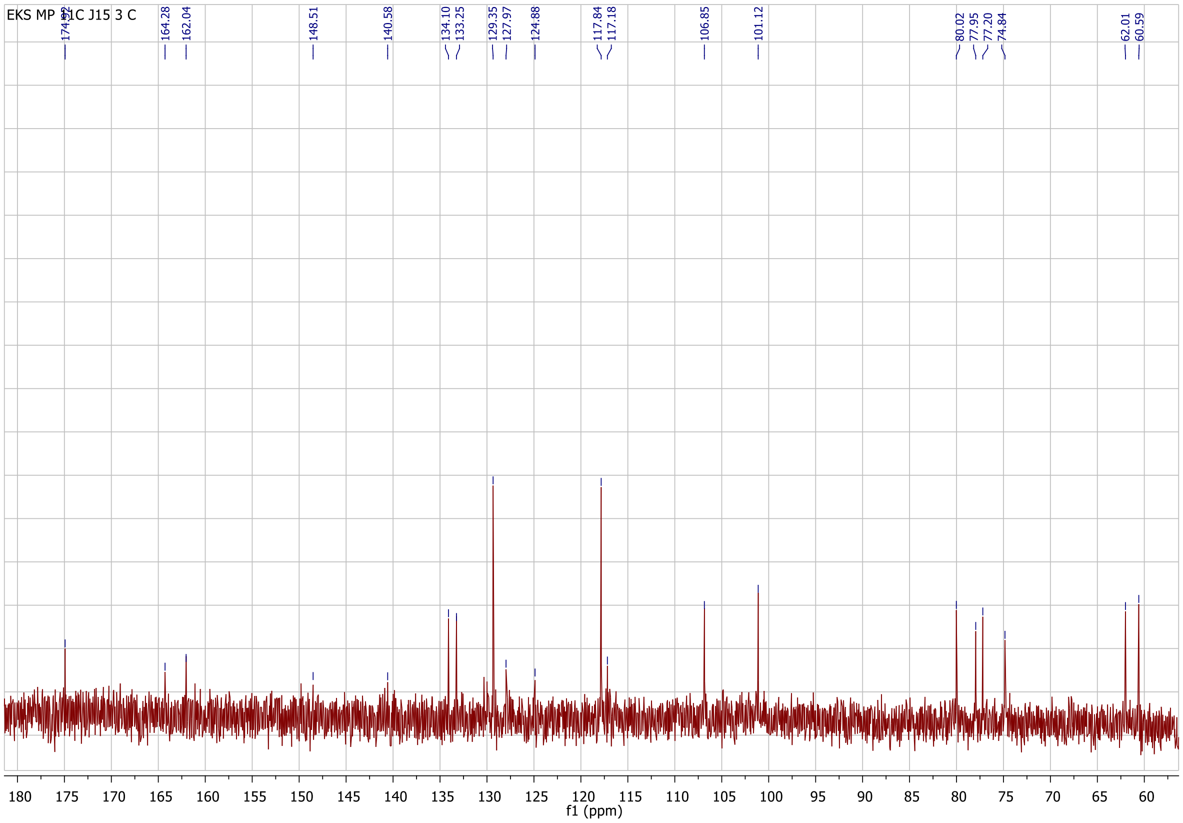

Figure S103. ¹³C NMR spectrum of 6-bromo-8-nitroflavone 4′-O-β-D-(4″-O-methyl)-glucopyranoside.

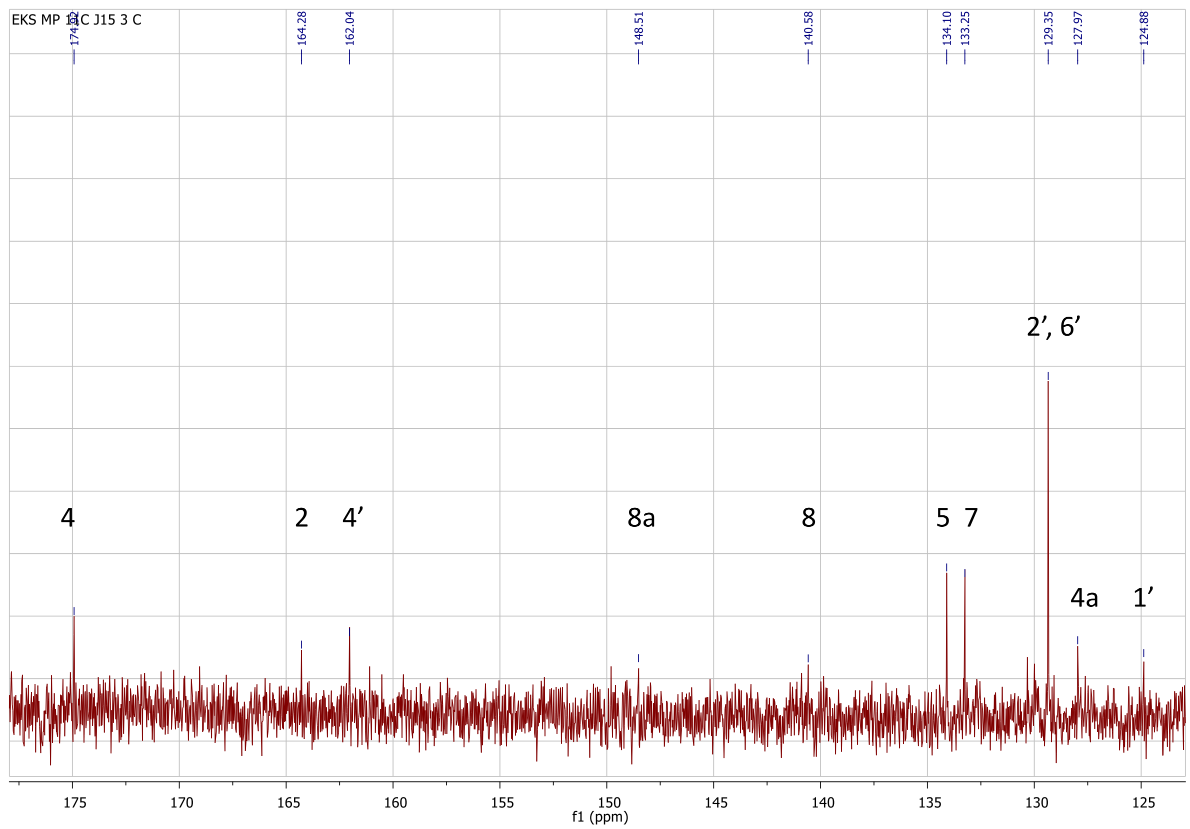

Figure S104. ^{13}C NMR spectrum expansion of 6-bromo-8-nitroflavone 4′-O-β-D-(4″-O-methyl)-glucopyranoside.

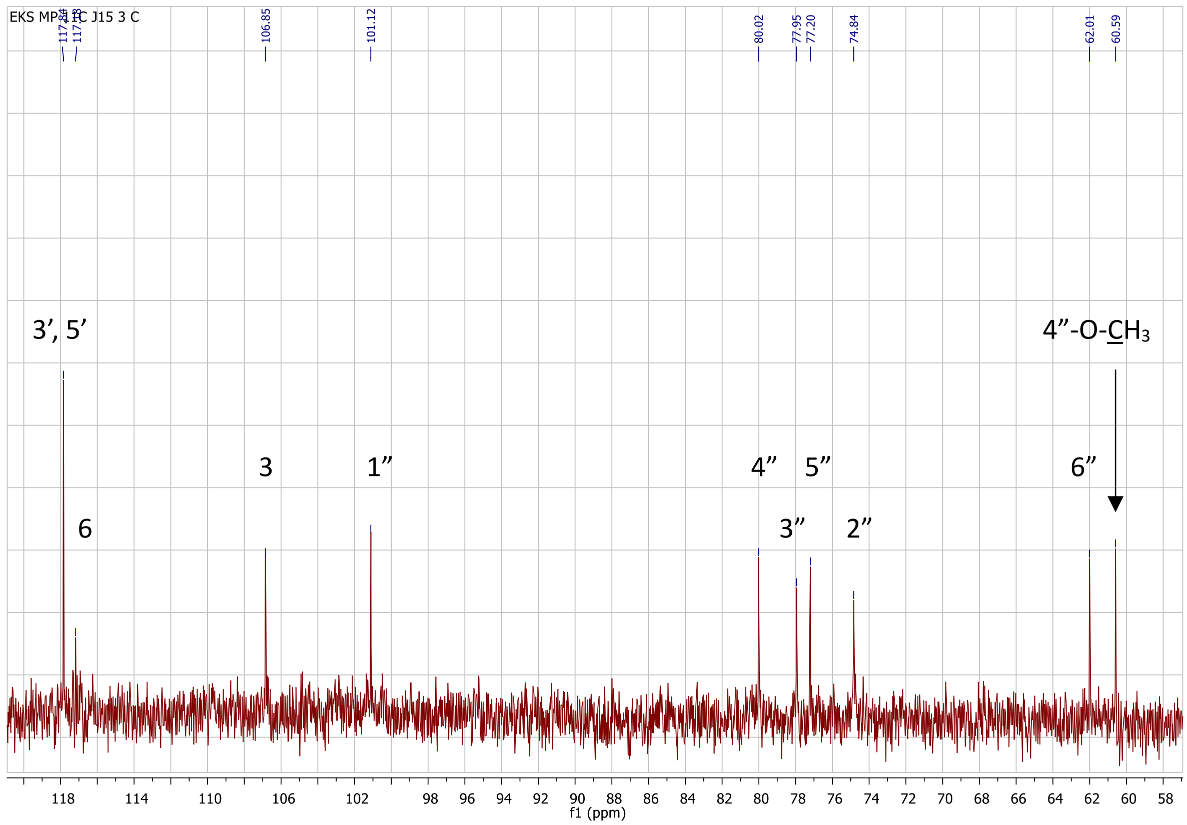

Figure S105. ^{13}C NMR spectrum expansion of 6-bromo-8-nitroflavone 4′-O-β-D-(4″-O-methyl)-glucopyranoside.

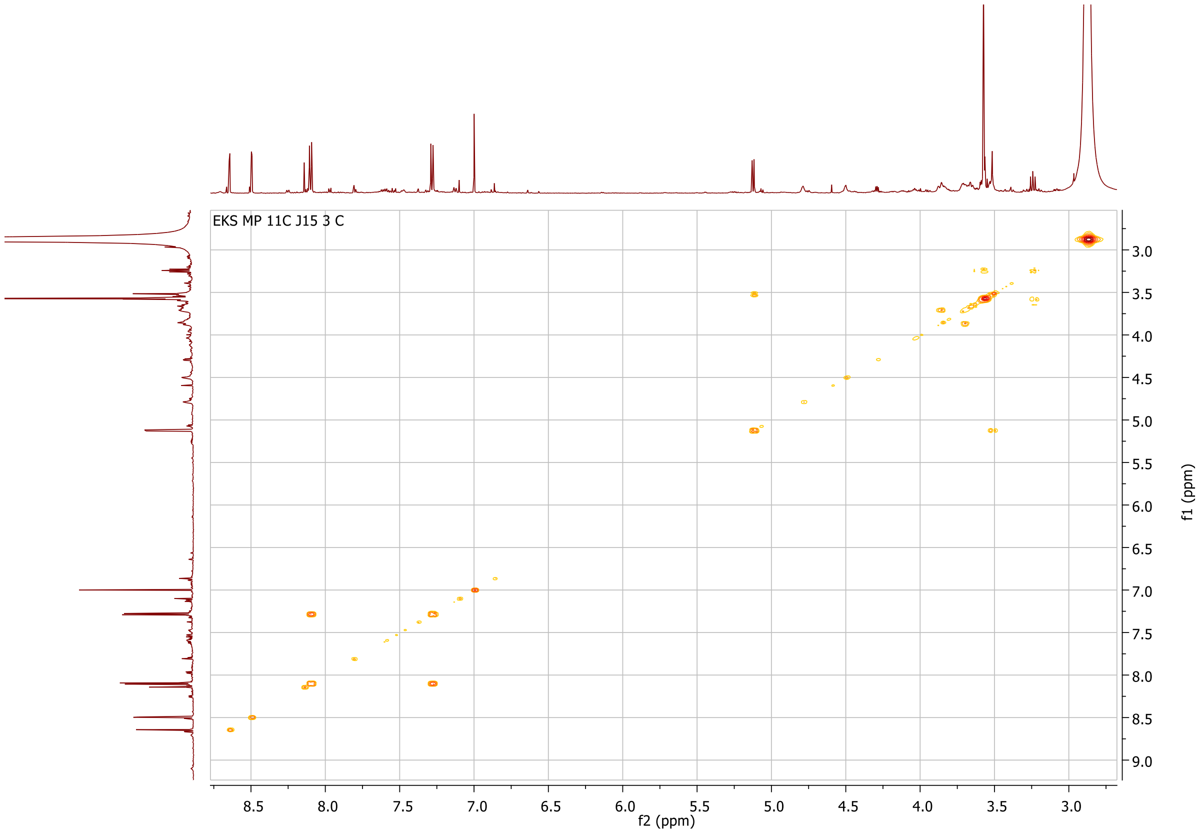

Figure S106. COSY contour map – ¹H x ¹H of 6-bromo-8-nitroflavone 4′-O-β-D-(4″-O-methyl)-glucopyranoside.

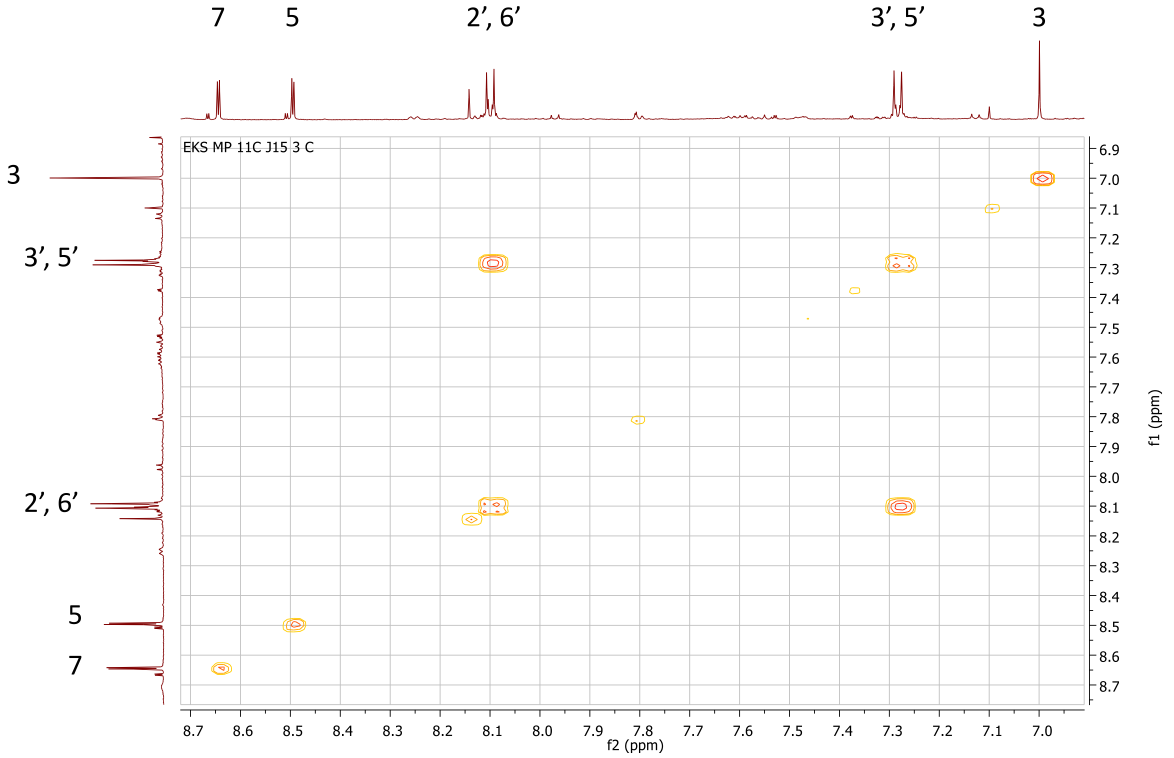

Figure S107. COSY contour map – ¹H x ¹H expansion of 6-bromo-8-nitroflavone 4′-O-β-D-(4″-O-methyl)-glucopyranoside.

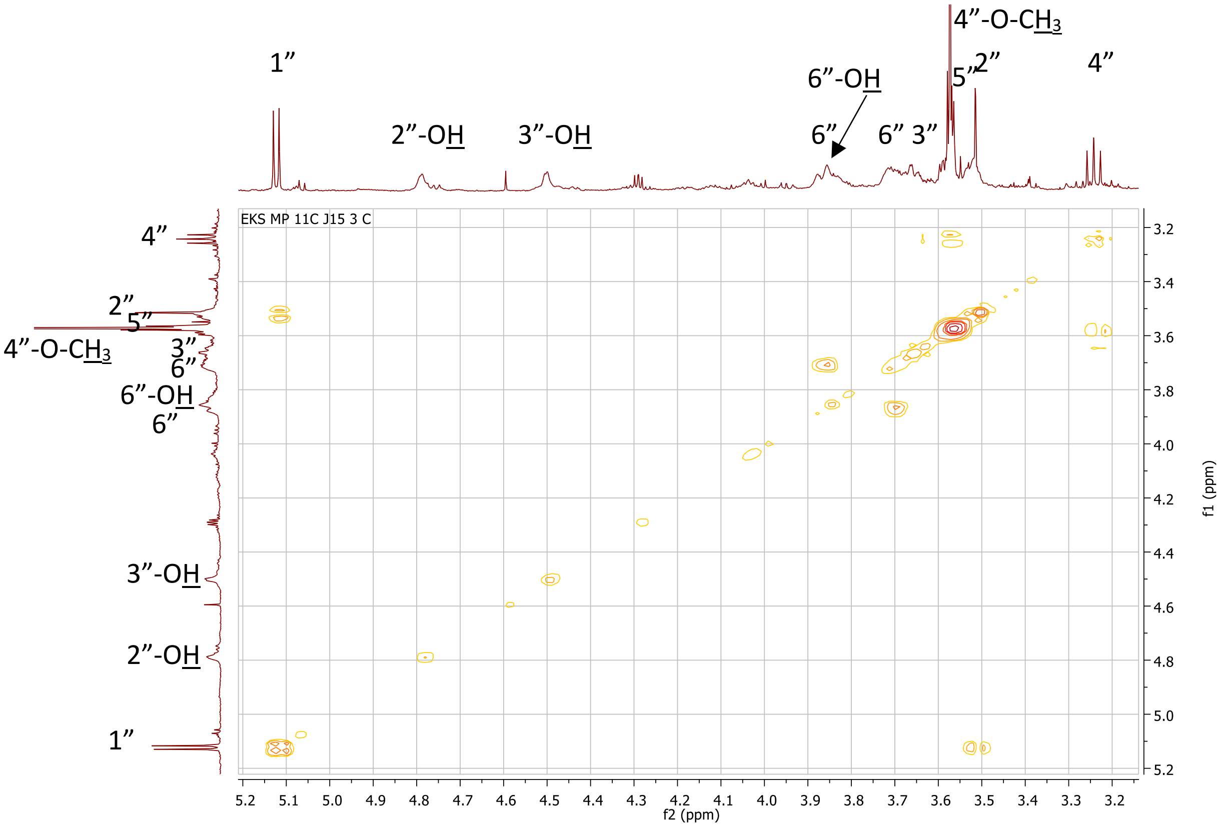

Figure S108. COSY contour map – ^1H x ^1H expansion of 6-bromo-8-nitroflavone 4′-O-β-D-(4″-O-methyl)-glucopyranoside.

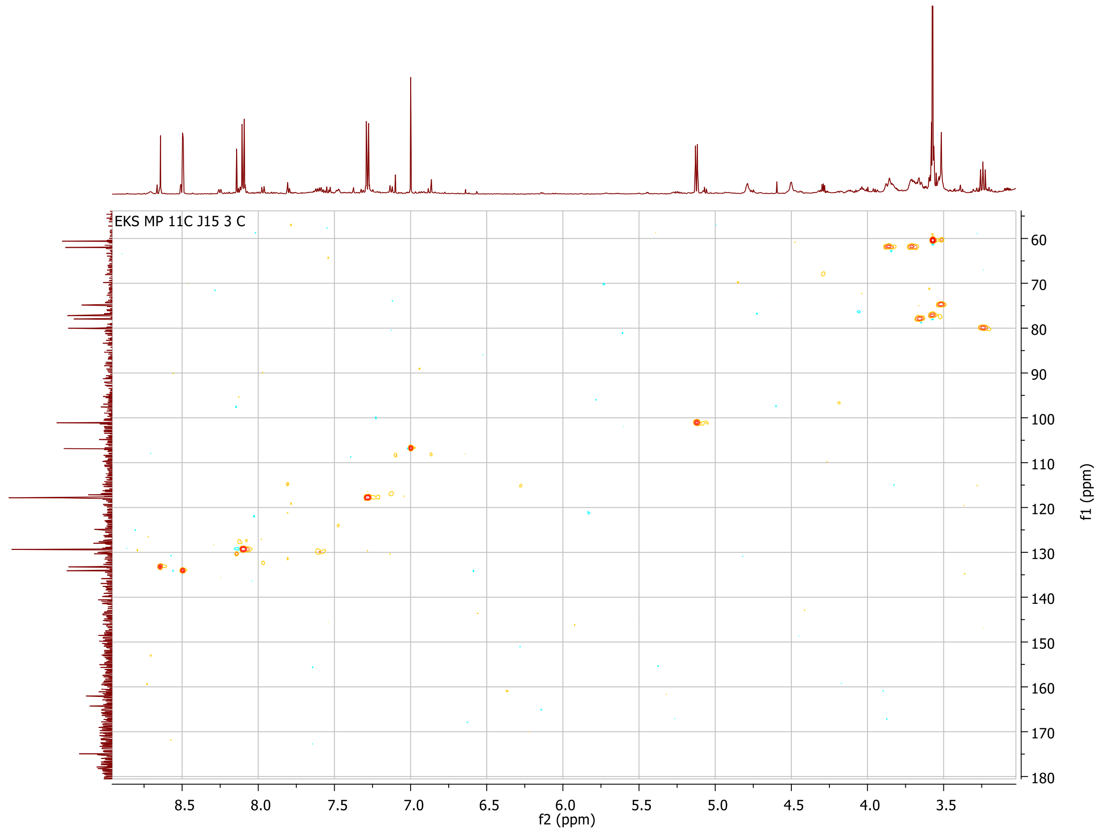

Figure S109. HSQC contour map – ^1H x ^{13}C of 6-bromo-8-nitroflavone 4′-O-β-D-(4″-O-methyl)-glucopyranoside.

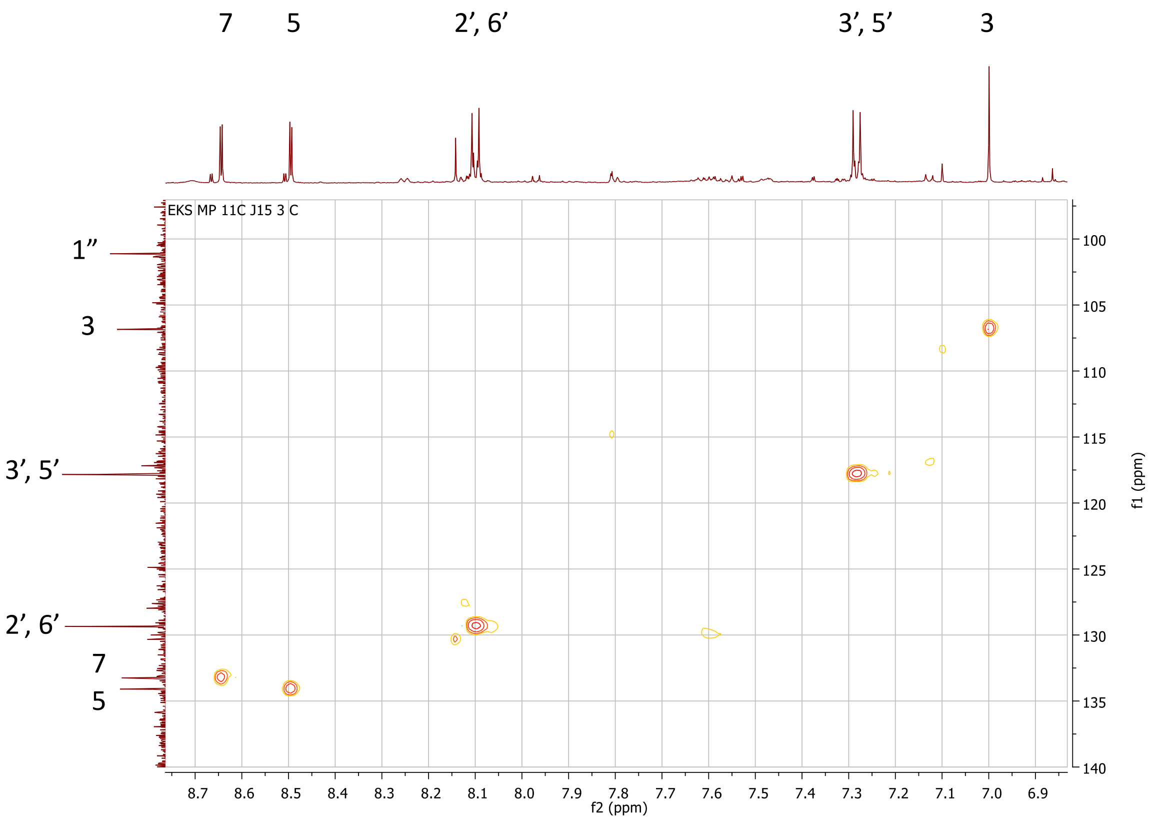

Figure S110. HSQC contour map – ¹H x ¹³C expansion of 6-bromo-8-nitroflavone 4′-O-β-D-(4″-O-methyl)-glucopyranoside.

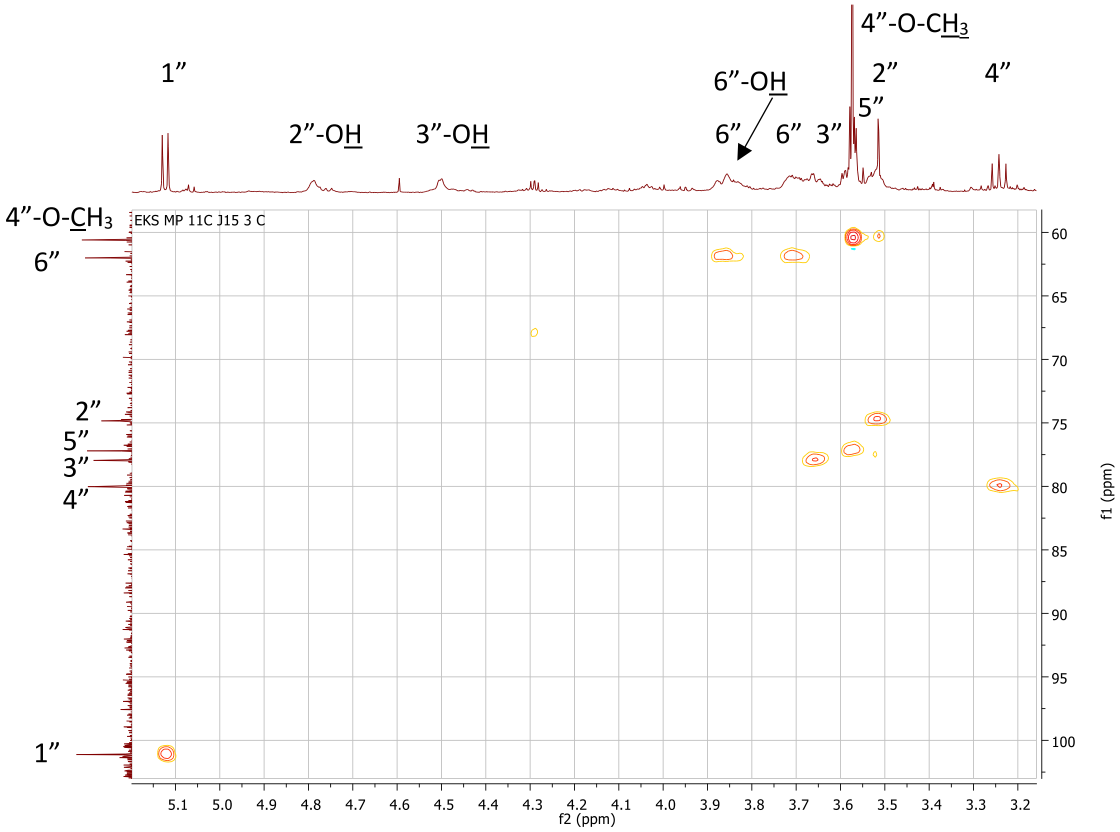

Figure S111. HSQC contour map – ¹H x ¹³C expansion of 6-bromo-8-nitroflavone 4′-O-β-D-(4″-O-methyl)-glucopyranoside.

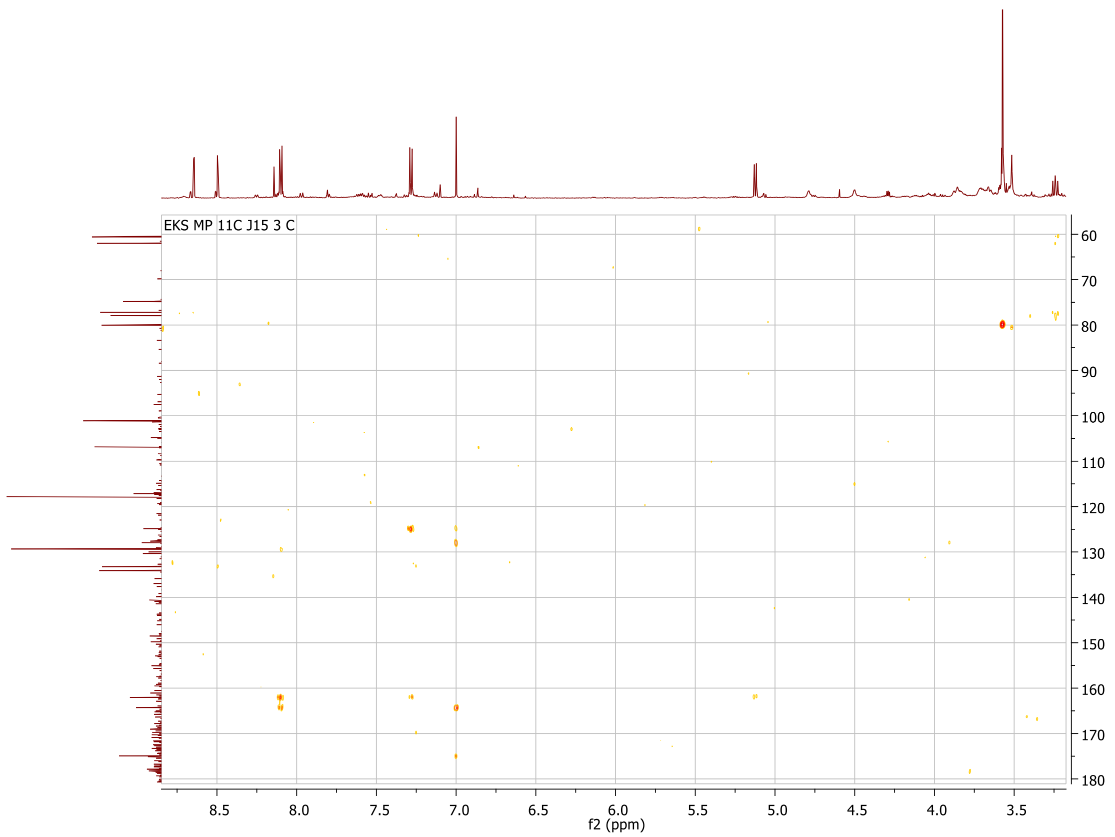

Figure S112. HMBC contour map – ¹H x ¹³C of 6-bromo-8-nitroflavone 4′-O-β-D-(4″-O-methyl)-glucopyranoside.

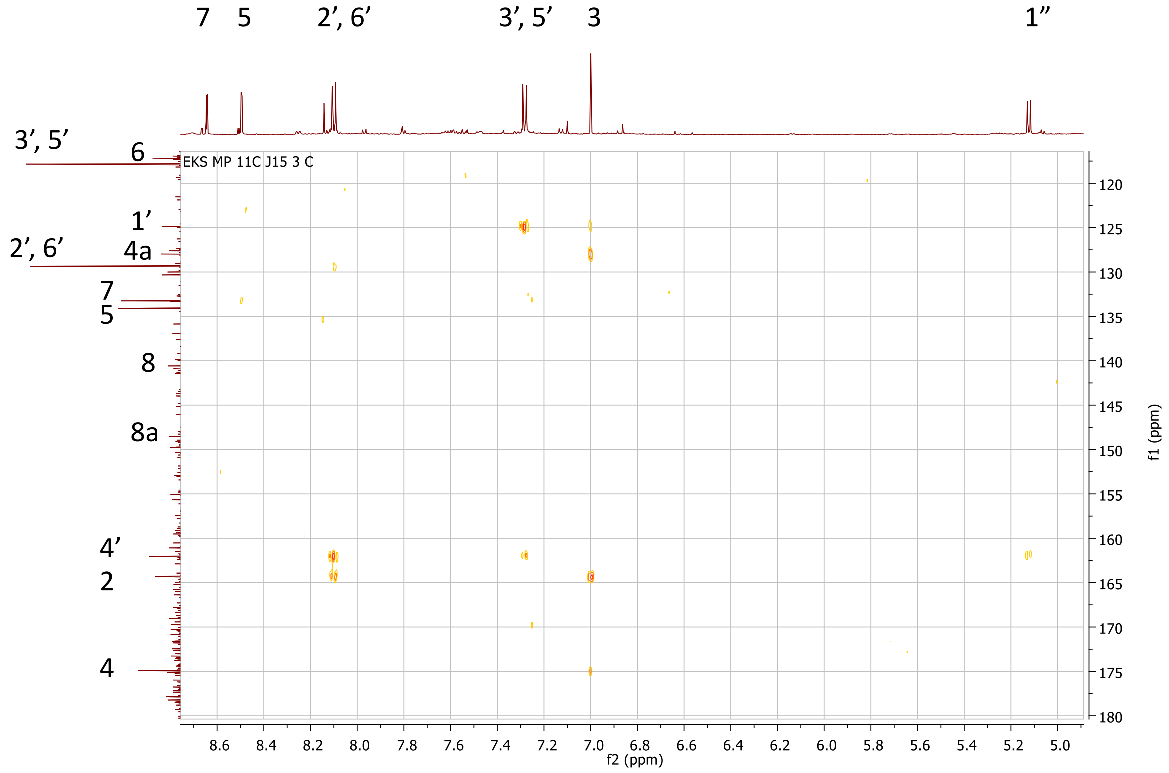

Figure S113. HMBC contour map – ¹H x ¹³C expansion of 6-bromo-8-nitroflavone 4′-O-β-D-(4″-O-methyl)-glucopyranoside.

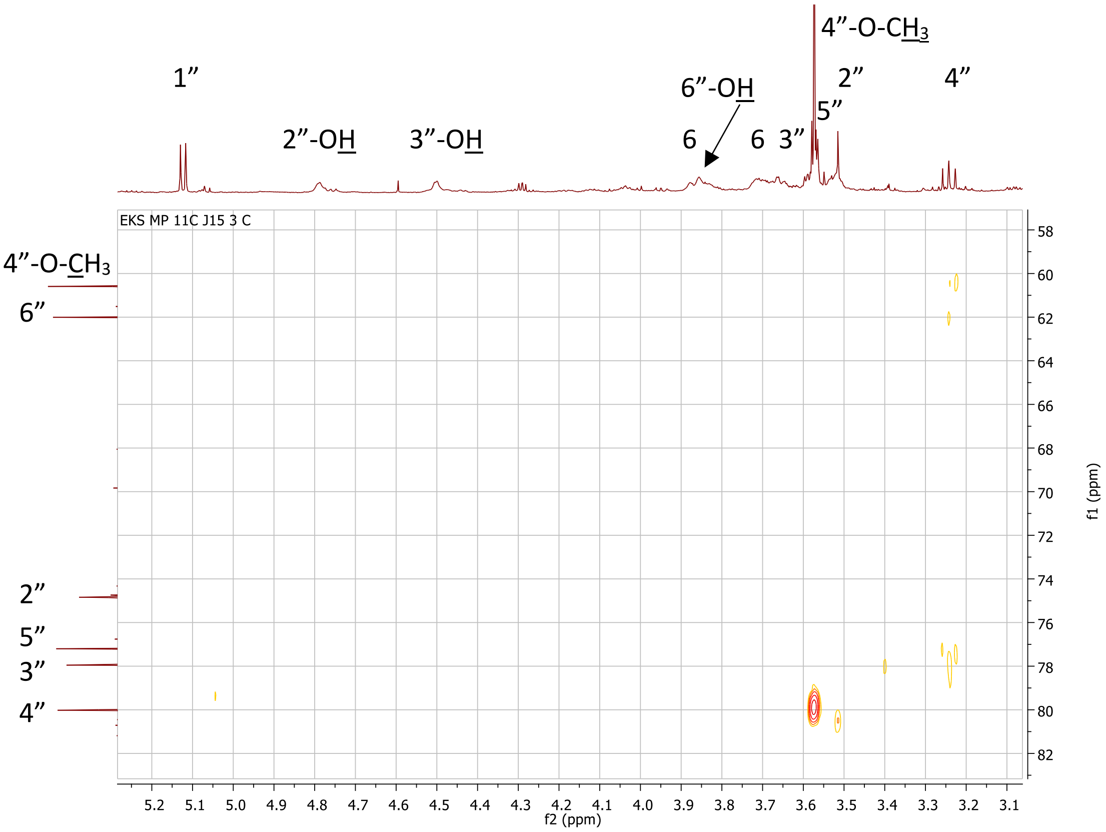

Figure S114. HMBC contour map – ^{1}H x ^{13}C expansion of 6-bromo-8-nitroflavone 4′-O-β-D-(4″-O-methyl)-glucopyranoside.
